# Oncogenic enhancers prime quiescent metastatic cells to escape NK immune surveillance by eliciting transcriptional memory

**DOI:** 10.1038/s41467-024-46524-0

**Published:** 2024-03-19

**Authors:** Daniela Michelatti, Sven Beyes, Chiara Bernardis, Maria Luce Negri, Leonardo Morelli, Naiara Garcia Bediaga, Vittoria Poli, Luca Fagnocchi, Sara Lago, Sarah D’Annunzio, Nicole Cona, Ilaria Gaspardo, Aurora Bianchi, Jovana Jovetic, Matteo Gianesello, Alice Turdo, Caterina D’Accardo, Miriam Gaggianesi, Martina Dori, Mattia Forcato, Giuliano Crispatzu, Alvaro Rada-Iglesias, Maria Soledad Sosa, H. T. Marc Timmers, Silvio Bicciato, Matilde Todaro, Luca Tiberi, Alessio Zippo

**Affiliations:** 1 Department of Cellular, Computational and Integrative Biology (CIBIO), University of Trento, 38123 Trento, Italy; 2 The South Australian Immunogenomics Cancer Institute, Faculty of Medicine Nursing and Medical Sciences, The University of Adelaide, Adelaide, Australia; 3 Istituto Italiano di Tecnologia IIT, Milan, Italy; 4 Department of Epigenetics Van Andel Institute, Grand Rapids, MI USA; 5 Department of Health Promotion, Mother and Child Care, Internal Medicine and Medical Specialties (PROMISE), University of Palermo, Palermo, Italy; 6 Department of Surgical, Oncological and Stomatological Sciences (DICHIRONS), University of Palermo, Palermo, Italy; 7 Department of Life Sciences, University of Modena and Reggio Emilia, Modena, Italy; 8 Center for Molecular Medicine Cologne (CMMC), University of Cologne, Cologne, Germany; 9 Institute of Biomedicine and Biotechnology of Cantabria (IBBTEC), CSIC/Universidad de Cantabria, Santander, Spain; 10 Department of Pharmacological Sciences, Icahn School of Medicine at Mount Sinai, New York, NY USA; 11 Department of Urology, Medical Center-University of Freiburg, Freiburg, Germany; 12 German Cancer Consortium (DKTK) partner site Freiburg, German Cancer Research Center (DKFZ), 69120 Heidelberg, Germany

**Keywords:** Metastasis, Chromatin, Cell-cycle exit, Transcriptional regulatory elements

## Abstract

Metastasis arises from disseminated tumour cells (DTCs) that are characterized by intrinsic phenotypic plasticity and the capability of seeding to secondary organs. DTCs can remain latent for years before giving rise to symptomatic overt metastasis. In this context, DTCs fluctuate between a quiescent and proliferative state in response to systemic and microenvironmental signals including immune-mediated surveillance. Despite its relevance, how intrinsic mechanisms sustain DTCs plasticity has not been addressed. By interrogating the epigenetic state of metastatic cells, we find that tumour progression is coupled with the activation of oncogenic enhancers that are organized in variable interconnected chromatin domains. This spatial chromatin context leads to the activation of a robust transcriptional response upon repeated exposure to retinoic acid (RA). We show that this adaptive mechanism sustains the quiescence of DTCs through the activation of the master regulator SOX9. Finally, we determine that RA-stimulated transcriptional memory increases the fitness of metastatic cells by supporting the escape of quiescent DTCs from NK-mediated immune surveillance. Overall, these findings highlight the contribution of oncogenic enhancers in establishing transcriptional memories as an adaptive mechanism to reinforce cancer dormancy and immune escape, thus amenable for therapeutic intervention.

## Abstract (web summary)

Metastasis arises from disseminated tumour cells (DTCs) while the underlying mechanism of DTCs plasticity remains underexplored. Here, the authors show that spatially organized oncogenic enhancers on chromatin sustain the establishment of retinoic acid (RA)-stimulated transcriptional memory through activation of SOX9, supporting the escape of quiescent DTCs from NK-mediated immune surveillance.

## Introduction

Cancer is both a genetic and an epigenetic disease whose outcome is influenced by systemic and microenvironmental signal^1^. These determinants represent the major driving forces of tumorigenesis, causing the functional heterogeneity that supports the adaptation of disseminated tumor cells (DTCs) to the foreign environments that they encounter during the invasion-metastasis cascade^2–4^. Recent findings suggested that epigenomic reprogramming plays a central role in cancer progression and metastasis formation, supporting the cell plasticity that, for example, enables DTCs to enter dormancy and escape immune surveillance^5–10^. Indeed, DTCs can enter a reversible state of dormancy as the result of a dynamic balancing between quiescent and proliferative signals provided by the microenvironment, immune, and resident cell^11–15^. Besides responding to acute exposure to this complex milieu of cues, DTCs could be able to adapt to recurrent exposures to the same stimulus, similarly to long-term stem cells which activate transcriptional memories in response to inflammation during wound healing^16–19^. Whether such an adaptive strategy fosters dormancy and which cell-autonomous mechanisms it supports is far from being understood. Cancer dormancy can last for years without any clinical manifestations^20^ and seemingly favors immune evasion from NK-mediated cytotoxicity^20–23^. It has been shown that DTCs escape immune surveillance by downregulating the cell surface ligands that permit NK cells to recognize and engage stressed, unhealthy cells^21,22^. Albeit this evidence, the molecular mechanisms controlling the maintenance of dormancy and how this cellular state favors the escape from immune surveillance are poorly defined.

Considering that the cellular response to both mechanical and biochemical stimuli is influenced by the chromatin state of *cis*-regulatory elements (CREs) such as promoters, enhancers, and insulators, it is not surprising that many of the epigenetic alterations affecting cancer cells have been associated with these genomic elements^23–27^. Enhancers are distal regulatory units whose activity depends on the binding of multiple transcription factors (TFs) including both lineage-determining and signal-dependent transcription factors (LDTFs and SDTFs, respectively), thus ensuring integration of intrinsic and extrinsic signals^8^. Occupancy of TFs at enhancers is associated with an open chromatin state, marked by specific histone modifications such as H3K4me1 and H3K27ac^28^. Enhancers work as multiple modules acting in an additive or synergic fashion to provide robustness and the spatiotemporal control of gene expression^28,29^. Indeed, cell identity and developmental genes are regulated by the concomitant action of multiple enhancers that act redundantly to provide phenotypic robustness^30–32^. It has been shown that the co-occurrence of long-range chromatin looping between promoters and multiple enhancers favors the segregation of CREs into transcriptional condensates, providing a possible mechanism by which transcription fidelity is maintained^33–37^. Whether a similar mechanism is involved in establishing a pro-metastatic transcriptional program has not yet been addressed. Indeed, although it has been shown that metastatic cells are characterized by the activation of de novo enhancers, there is scant evidence supporting their role in tumorigenesis, thereby acting as oncogenic enhancers^24–27^. Moreover, it is still poorly defined at which stage of the metastatic cascade the rewiring of the epigenetic state of enhancers contributes most.

To address these questions, we develop an in vivo model recapitulating the onset and progression of breast cancer, leading to the dissemination of metastatic cells into secondary organs. We find that tumor progression is coupled with epigenetic reprogramming at CREs that defines the activation of metastatic-specific enhancers. By combining 3D chromatin conformation mapping with graph-based network analysis, we define interconnected chromatin domains (iCDs) characterized by multiple, yet low frequent, CREs interactions. Of importance, we establish that while iCDs per se do not predict the average expression level of the linked genes, they support the establishment of transcriptional memory. Single-cell analyses combined with epigenome editing of active enhancers show that enhancer redundancy specifies transcriptional memory in metastatic cells that have been previously exposed to retinoic acid (RA). We find that RA-dependent transcriptional memory sustains quiescence in metastatic tumoroids, which is mediated by the transcriptional modulation of SOX9. This adaptive mechanism increases the fitness of DTCs by supporting the evasion from NK-mediated immune surveillance.

## Results

### Metastasis-derived tumoroids preserve phenotypic features of metastases

**Fig. 1:**
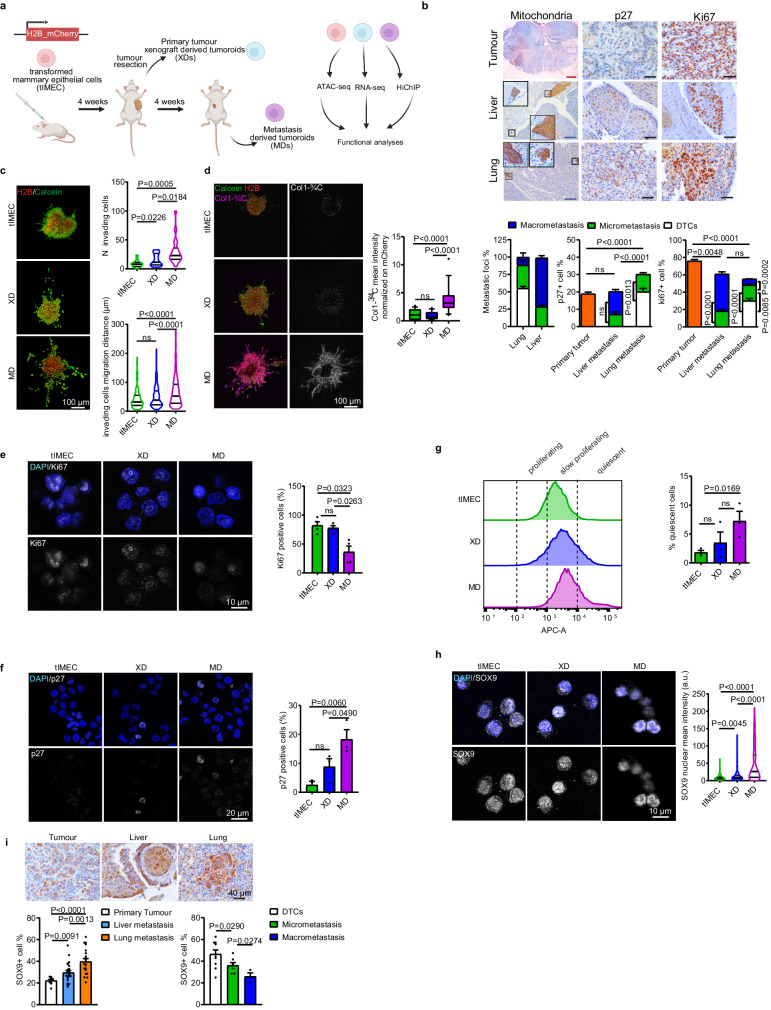
Recapitulating metastatic progression of TNBC ex-vivo. **a** Schematic representation of model derivation and analysis approach (created with BioRender.com). **b** Representative images of IHC and quantification of metastatic size (DTCs: *n* < 10 cells; micro-metastasis: 10 <  *n* < 100 cells; macro-metastasis: *n* > 100 cells), p27 and Ki67 positive cells in lung and liver metastases, and primary tumor. Quantification was performed on *n* = 4–16 Field of Views (FOVs), merged from three independent experiments. For liver and lung tissues, a representative image showing both micro- and macro-metastases is shown, with corresponding zoomed-in crops (black: micro-metastasis; gray: macro-metastasis). Red scale bar = 1 mm; blue scale bar = 100 µm; black scale bar = 40 µm. **c** Representative images of tIMEC, XD, and MD tumoroids embedded in collagen and quantification of seeding cells and distance from the spheroid. Calcein AM (green); H2B-mCherry (red); scale bar: 100 µm. **d** Representative images of tIMEC, XD, and MD tumoroids embedded in collagen and quantification of Col-¾ mean intensity. Calcein AM (green); H2B-mCherry (red); Col-¾ (purple) scale bar: 100 µm. **e** Representative images and quantification of Ki67 positive cells in tIMEC, XD, and MD cells. Ki67, white; DAPI, blue; scale bar = 10 µm. **f** Representative images and quantification of p27 positive cells in tIMEC, XD, and MD cells. p27, white; DAPI, blue; scale bar = 10 µm. **g** Representative histograms of FACS analysis on dye-retaining (APC-A) cells and quantification of APC-A+ cells. **h** Representative images and quantification of SOX9 nuclear mean intensity in tIMEC, XD, and MD cells. SOX9, white; DAPI, blue; scale bar = 10 µm. **i** Representative images of IHC and quantification of SOX9 positive cells in primary tumor, lung, and liver metastases and in subgroups (DTCs, micro- and macro-metastasis) of lung metastasis. Quantification was performed on *n* = 4–24 Field of Views (FOVs), merged from three independent experiments. The barplots in (**b**), (**f**–**i**) are means of three independent biological replicates ± S.E.M. The barplots in (**e**) are means of four independent biological replicates ± S.E.M. The box plots in (**d**) indicate median values (middle lines), first and third quartiles (box edges), and 10th and 90th percentiles (error bars) retrieved from three independent biological replicates. The violin plots in (**c**) and (**h**) indicate median values (middle lines), first and third quartiles (dashed lines) retrieved from three independent biological replicates. Statistical significance was determined by one-tailed unpaired student’s *t*-test.

To address the contribution of epigenome reprogramming to breast cancer progression and metastasis formation, we setup an orthotopic xenograft assay that recapitulates the onset and the metastatic relapse of Triple-negative breast cancer (TNBC)^27^. After implanting transformed mammary epithelial cells (tIMEC) into the mammary gland of NOD-Prkdc^scid^ (NOD-SCID) immunocompromised mice, we surgically resected the grown primary tumors and monitored the animals to detect tumor relapse locally and at distal sites (Fig. 1a). Although bioluminescent imaging did not reveal the presence of metastatic lesions, IHC analyses of the lung revealed the presence of scattered DTCs and micrometastases, with few macro-metastasis that were most abundant in the liver (Fig. 1b). We observed that the metastatic lesions were composed of a substantial fraction of cycling (Ki67+) cells. Of importance, we also detected quiescent (p27+) cells, which were enriched in the lung with respect to lung metastatic lesions (Fig. 1b). By setting up 3D culture conditions, we retrieved tumoroids from both primary tumors and metastatic lesions (Fig. 1a and Supplementary Fig. 1a). Xenograft- and metastatic-derived tumoroids (hereafter named XD and MD, respectively) showed a comparable sphere-forming efficacy and expression of the stem cell markers ALDH1 and BMI-1 (Supplementary Fig. 1b–d), thus preserving the self-renewal capability retrieved from the parental transformed cells (tIMEC). By performing a three-dimensional tumor spheroid invasion assay, we quantified the capability of cancer cells to degrade the surrounding extracellular matrix (ECM) and to seed at distant sites (Fig. 1c, d). The obtained results showed that MD cells were able to invade the surrounding microenvironment by degrading the ECM protein Collagen I (Fig. 1c, d). These findings were further corroborated by measuring the mobility of cancer cells in response to a chemo-attractor, showing that MD possessed a considerably higher migration capacity with respect to tIMEC and XD cells (Supplementary Fig. 1e). We further evaluated whether the MD tumoroids maintained cellular plasticity, similarly to the quiescent pattern retrieved in the lung metastatic lesions. By performing immunofluorescence analyses, we found that the fraction of cycling (Ki67+) cells was decreased in MD tumoroids, with respect to both XD and tIMEC (Fig. 1e). Conversely, in the same experimental setting, we measured an increased number of quiescent (p27+) cells in the MD tumoroids (Fig. 1f and Supplementary Fig. 1f). A dye retention assay confirmed that within MD tumoroids a fraction of cells was not actively dividing, irrespective of the growth stimulating conditions, favored by optimal mammalian cell culture conditions (Fig. 1g). Of note, MD tumoroids were characterized by an increased, yet heterogeneous expression of SOX9, a pro-metastatic TF involved in cancer cell dormancy (Fig. 1h)^11,22^. Remarkably, a similar pattern was also depicted in metastatic lesions, which showed an increased level of SOX9 abundance with respect to the primary tumor (Fig. 1i). In addition, the fraction of SOX9 positive cells was higher among the DTCs and micro-metastasis, with respect to macrometastatic lesions. In summary, these data indicated that the metastatic-derived tumoroids preserved the phenotypic features that characterize metastatic cells, including invasion capability and quiescence.

### Metastatic cells activate an alternative epigenetic program

**Fig. 2:**
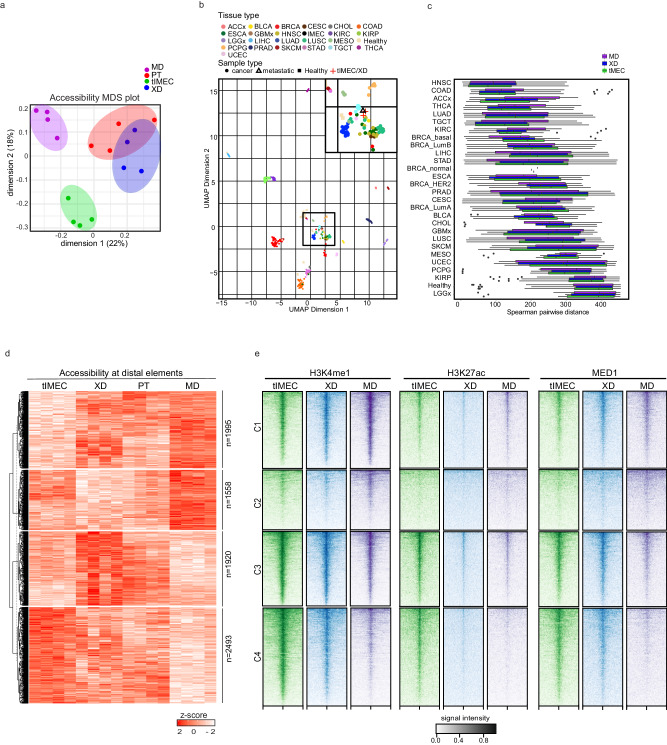
Metastatic onset is associated with epigenomic rewiring. **a** Multidimensional scaling (MDS) plots of batch-corrected logCPM values with samples (tIMEC, XD, PT, and MD) colored by cell type. Distances on the plot correspond to the leading fold change, which is the average (root-mean-square) log2fold change for the 5000 most divergent peaks between each pair of samples. **b** Uniform Manifold Approximation and Projection (UMAP) projections of tIMEC, XD, and MD accessibility data along with publicly available accessibility datasets representing human primary cancer samples from different locations, human primary non-cancerous tissues from different locations. A zoomed-in crop highlighting tIMEC, XD, and MD samples is shown. **c** Boxplot showing Spearman pairwise distance between tIMEC, XD, MD, IMEC samples, and the human primary tumor tissues in the TCGA dataset ordered by similarity (*n* = 4). **d** Heatmap showing chromatin accessibility of the 7966 differentially accessible regions for the tIMEC, XD, PT, and MD samples (z-score of the log2-CPM is presented, cut-off log2fold change > 1 and FDR < 0.1) in the four biological replicates. **e** Density plots of H3K4me1, H3K27ac, and MED1 CUT&RUN signals in the four differential clusters identified by ATAC-seq in tIMEC, XD, and MD cells. Signal distribution is centered on the TSS peak and ranges from −5kB to 5kB. Greyscale signal intensity legend is valid for the three cell lines. The box plots in (**c**) indicate median values (middle lines), first and third quartiles (box edges), and 10th and 90th percentiles (error bars).

Considering the phenotypic diversity retrieved by comparing transformed cells with XD and MD tumoroids, we tested whether a distinct chromatin state characterized the metastatic cells. We focused on determining the contribution of CREs in defining the cell-specific epigenetic state by profiling their chromatin accessibility landscape. We performed ATAC-seq on tIMEC, primary tumors (PT) samples retrieved directly from orthotopically transplanted mice, and the corresponding XD and MD tumoroids. Although the genomic distribution and the chromatin state of the mapped CREs did not differ among the analyzed samples (Supplementary Fig. 2a, b), Multidimensional scaling (MDS) plot of most variable ATAC-seq regions showed that MD tumoroids were characterized by a distinct chromatin accessibility landscape with respect to the other samples (Fig. 2a). Of note, XD, and PT clustered closely, indicating that the tumoroids preserved the chromatin state of the parental tumors. We further assessed whether the epigenetic landscape of tIMEC, PT, XD, and MD would reflect the shared traits of human cancers. Uniform Manifold Approximation and Projection (UMAP) plot of ATAC-seq profiles from 410 tumor samples spanning 23 cancer types^38^ and non-cancerous tissues retrieved from different locations^39^ showed that tIMEC, PT, XD, and MD clustered along with human cancers and separated from healthy tissues (Fig. 2b and Supplementary Fig. 2c). Spearman pairwise distance showed that the chromatin landscape of tIMEC, XD, and MD was closer to multiple carcinoma types, including breast cancer, while healthy tissues resulted among the most distant samples together with brain tumors (Fig. 2c and Supplementary Fig. 2c).

To investigate the contribution of putative enhancers to establish the phenotypic features of metastatic cells, we performed cluster analyses on the differentially enriched distal CREs, defined by chromatin accessibility profiling. We found that MD cells were characterized by the most distinctive accessibility pattern, with 3553 differentially enriched distal sites (Fig. 2d, Supplementary Data 1). We identified clusters of MD-enriched CREs, which showed a marked increase in chromatin accessibility (C1 and C2), and MD-depleted CREs that were specifying the transformed cancer state (C3 and C4). To further evaluate whether the putative CREs harbored a chromatin pattern that distinguishes enhancers, we performed calibrated CUT&RUN for the histone marks H3K4me1, H3K27ac, and the transcription cofactor MED1 (Fig. 2e). We found that the differentially enriched CREs represented putative enhancers, as they were marked by H3K4me1 (Supplementary Fig. 2d). Comparative analyses showed that the MD-enriched CREs (C1-C2) were characterized by an increment of H3K4me1 levels in metastatic cells, with respect to tIMEC and XD. However, while the C1 showed a relative enrichment for the active marks H3K27ac and MED1 in MD cells, the CREs belonging to C2 did not show the same increment suggesting that these CREs may represent poised enhancers (Fig. 2e and Supplementary Fig. 2d). The MD-depleted CREs (C3-C4) were characterized by a broad distribution of H3K4me1 in tIMEC, whose level decreased in the XD and MD cells. This pattern was mirrored by a concomitant reduction of both H3K27ac and MED1 levels, indicating enhancer decommissioning (Fig. 2e and Supplementary Fig. 2d). Of importance, profiling of chromatin accessibility and histone marks patterns at promoters did not show the same level of reshaping, with a minority of proximal CREs gaining H3K4me3 in the MD (Supplementary Fig. 2e, f, Supplementary Data 2). In sum, these results indicated that tumor progression is characterized by rewiring of the chromatin state, specifically at enhancers.

### MD-enriched CREs are spatially organized in interconnected chromatin domains

**Fig. 3:**
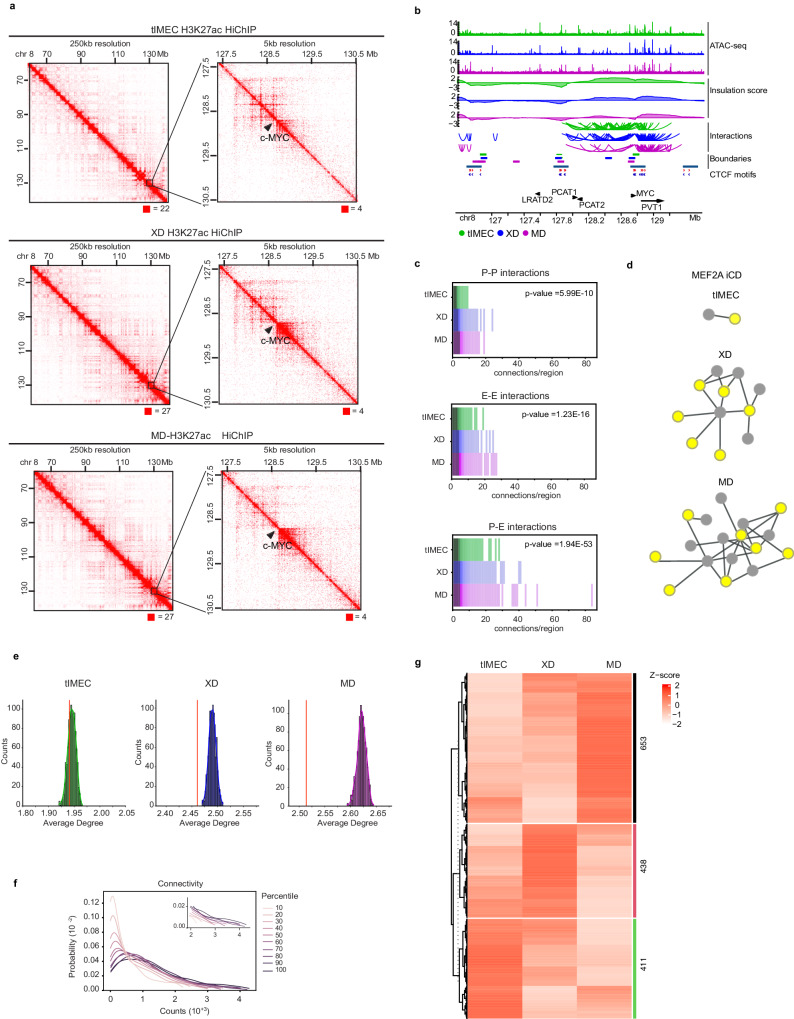
Long-range interactions affect chromatin looping during tumor progression. **a** H3K27ac HiChIP raw interaction maps of the *MYC* locus in tIMEC, XD, and MD cells (from top to bottom). 250 kb to 5 kb resolution (left to right). *MYC* locus (window = 80 Mb left, 3 Mb right). Numbers below the interaction maps correspond to maximum signal in the matrix. **b** Snapshot of the genomic locus surrounding the *MYC* locus (hg19, chr8:126,557,447-129,597,447), showing the ATAC-seq signal, insulation scores, H3K27ac interactions defined by HiChIP and identified TAD boundaries for tIMEC, XD, and MD. In addition, boundaries for TNBC cell lines (HCC1599 and MB157) and CTCF sites within these boundaries are shown. **c** Comparison of degree distributions (connection/region), according to type of connection: Promoter-Promoter (P-P), Enhancer-Enhancer (E-E), and Promoter-Enhancer (P-E). *p*-values have been computed, using the Kruskal–Wallis *H*-test. **d** Representation of the iCD of MEF2A Promoter (yellow) - Enhancer (gray) interactome in tIMEC (top), XD (middle), and MD (bottom). **e** Network average degree after MD-specific CREs removal (red line) *versus* network average degree after the removal of the same number of random regions (distribution) in tIMEC (left), XD (center), and MD (right) cells. **f** Kde plot, showing the relationship between the abundance of expressed genes (y-axis) and their level of expression (x-axis). Genes are clustered in 10 percentiles, according to their level of HiChIP connectivity. **g** Heatmap of differential RNA-seq transcripts z-scores. Genes are divided in three clusters, whose count is expressed on the right.

To determine the contribution of enhancers in modulating the gene expression pattern of the metastatic cells, we mapped the genome-wide chromatin interactions occurring among the CREs. Given that both active promoters and enhancers are characterized by the deposition of H3K27ac, we captured chromatin domains enriched for this histone mark by HiChIP^40^. Comparative analyses of the contact domains showed that the retrieved 3D chromatin organization was comparable among tIMEC, XD, and MD cells, and similar to what has been defined by in situ HiC in IMR90 (Fig. 3a and Supplementary Fig. 3a)^41^. Furthermore, the contact domain boundaries identified by H3K27ac HiChIP were concordant with those identified in independent TNBC cell lines using SMC1 HiChIP datasets (Fig. 3b and Supplementary Fig. 3b, c)^42^. These results indicated that the HiChIP dataset was reliable for the correct identification of chromatin interactions occurring among active CREs. After filtering for highly significant interactions (*q* < 0.01), we identified a total of 160.000 chromatin loops, capturing Enhancer-Promoter (E-P), Promoter-Promoter (P-P) and Enhancer-Enhancer (E-E) contacts with a similar distribution among the analyzed samples (Supplementary Fig. 3d). Of note, we retrieved that most of the chromatin connections (60%) occurred among E-P regions, with a consistent fraction of them forming multiple interactions, similarly to highly connected CREs (Supplementary Fig. 3d)^35^. To gain biological insights into the relevance of these regulatory interactions, we analyzed the topological organization of CREs by using graph-based network analysis (Supplementary Fig. 3e). Visualization of the whole spatial chromatin organization showed that the interconnected chromatin domains (iCDs) of metastatic cells were larger, as resulting from multiple interactions with a mean degree of 2.73, with respect to 1.98 and 2.56 for tIMEC and XD, respectively (Fig. 3c–e). Given that the HiChIP dataset results from bulk analyses of the interactome, we can not exclude that the depicted iCDs are the result of multiple single-cell chromatin states^43^. Nevertheless, the high frequency of measured connections within the same iCDs can be interpreted as the frequency of interactions between the paired regions. Indeed, by comparing the graphs of the three biological systems, we found that on average metastatic tumoroids exhibited a higher degree of CREs along the promoter-enhancers (P-E) interactome (Fig. 3c, d). The iCDs featured by the highest degree in MD cells showed a reduced frequency of interactions in XD and tIMEC (Fig. 3c, d), suggesting that the epigenome rewiring occurring during tumor progression may increase interconnectivity among the active CREs. Of note, by measuring the impact of the MD-enriched CREs to the degree of the interactome, we found that they specifically contributed to the degree of iCDs in metastatic tumoroids (Fig. 3e). To gain biological insights, we investigated whether the features characterizing the P-E interactions could predict the pattern of gene expression depicted by RNA-seq in MD tumoroids (Supplementary Fig. 3f). Plotting the gene expression level as a function of P-E connectivity showed a nonlinear relationship, with the lowest expressed genes being characterized by the smallest frequency of interactions (Fig. 3f). In parallel, we could predict that iCDs characterized by a higher connectivity harbored the most expressed genes (Fig. 3f and Supplementary Fig. 3g). This nonlinearity is further exemplified by plotting the relative changes in the expression level of the genes linked to MD-enriched CREs (Fig. 3g). We found that a large fraction of the linked genes showed an increment of the transcript level in the MD tumoroids with respect to XD and tIMEC. At the same time, other transcripts showed a different pattern, being unchanged or even decreased in the MD, irrespective of their interactions with distal enhancers that were specifically activated in metastatic cells (Fig. 3g). These results suggest that tumor progression is coupled with a reorganization of the genome topology of CREs, which in itself did not predict the gene expression pattern of the linked genes.

### TFs delineate the activity of metastatic-specific enhancers

**Fig. 4:**
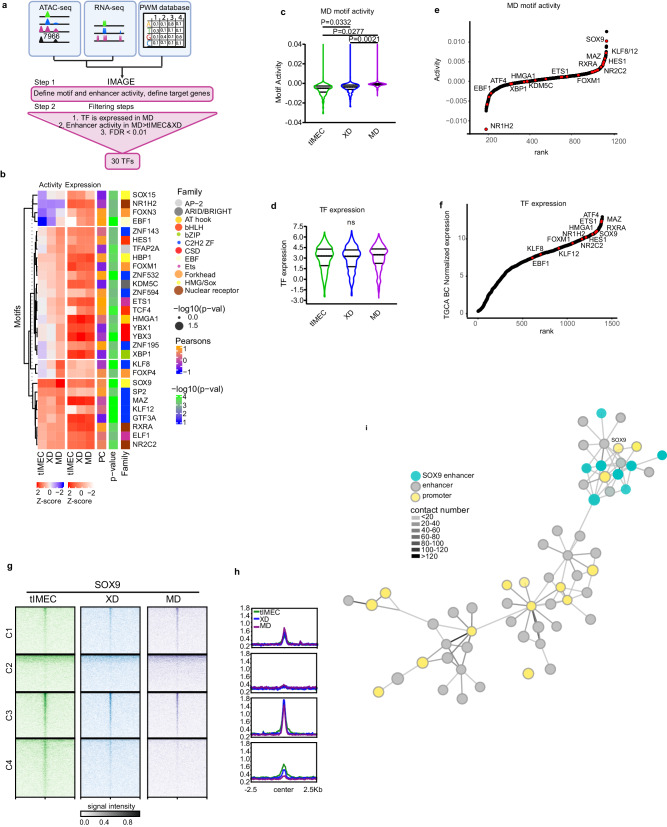
TFs contributes to the activation of Metastatic-specific enhancer. **a** Scheme of the ATAC-seq and RNA-seq data integration workflow using the IMAGE machine learning approach (created with BioRender.com). **b** Clustering of top MD-associated motifs with a motif activity MD > tIMEC and XD cells and defined as causal for gene regulation with an FDR < 0.01. Additional columns show z-score normalized expression of motif-associated TF, PCC between motif activity, and gene expression of associated TF and the associated TF family. Dot size for the TF family indicates enrichment in the unfiltered list by hypergeometric *t*-test. **c** Violin plots showing activity of MD-enriched motifs in tIMEC, XD, and MD cells, with an activity MD > tIMEC/XD. **d** Violin plots showing normalized expression values of motif-associated TFs showing a higher motif activity in MD compared to tIMEC or XD. **e**, **f** Distribution plot showing the rank of the TFs identified as drivers of MD enhancer activity among all TFs considered for the analysis (**e**) or expressed in the TCGA BC dataset (**f**). **g** Density plots showing the SOX9 CUT&RUN signal in the four differential ATAC-seq clusters identified. Signal distribution is centered on the TSS peak and ranges from −5kB to 5kB. Greyscale signal intensity legend is valid for the three cell lines. **h** Cumulative plots showing SOX9 CUT&RUN signal at peaks identified by ATAC-seq for the four defined clusters. **i** Representation of SOX9 iCD in MD cells. Vertices colors represent SOX9*-*linked enhancers (light blue), enhancers (gray), and promoters (yellow). The grayscale of edges is proportional to the number of connections between vertices. The violin plots in (**c**) and (**d**) indicate median values (middle lines), first and third quartiles (dashed lines). Statistical significance was determined by one-tailed unpaired student’s *t*-test.

To determine whether the MD-enriched CREs are functional to tumor progression, we profiled enhancer activity by means of determining the contribution of TFs binding to gene expression changes (Fig. 4a). To this end, we integrated CREs mapping and interaction with gene expression profiling and searching for TF motifs using an extended position weight matrices (PWM) database to identify TFs causal for gene expression changes by IMAGE (Fig. 4a)^44^. By inferring the contribution of 896 expressed TFs to the activity of the MD-enriched CREs, we identified many TFs motifs that have been previously described to play a role in breast cancer progression and metastasis (Fig. 4b, Supplementary Data 3), including EBF1, HE1, HMGA1, XBP1 and SOX9. Within this group, we found that TFs belonging to the nuclear receptor family resulted in being overrepresented, primarily enriched for the retinoic acid receptors, which are activated in human metastasis^45,46^ (Fig. 4b). Of note, the transcription factors that contributed most to MD enhancer activity showed a low level of activity in tIMEC, which was further augmented in XD and MD (Fig. 4c), despite their expression level being almost unchanged (Fig. 4d). By ranking them by their activity, we found that SOX9 was among the most enriched TFs in the metastatic tumoroids (Fig. 4e and Supplementary Fig. 4a, b) and was expressed in breast cancer (Fig. 4f and Supplementary Fig. 4c). Of note, the same enrichment was not retrieved when we performed the IMAGE analysis considering all the distal CREs, suggesting that the identified TFs specifically contribute to the activity of the MD-enriched enhancers (Supplementary Fig. 4d and Supplementary Data 4). To exclude that the approach based on the differential chromatin accessibility to identify MD-regulated CREs could influence the outcome of these analyses, we retrieved the same information by using as input for IMAGE the compendium of distal CREs that are part of the iCDs. We found that many of the key motifs of TFs were shared among the two datasets, including XBP1, GTF3A, and SOX9 (Supplementary Fig. 4e and Supplementary Data 5), corroborating the relevance of this approach. To experimentally validate these results, we profiled the chromatin binding of SOX9 by CUT&RUN and found that it was enriched at the distal CREs (Fig. 4g, h). In the MD tumoroids SOX9 binding was augmented at the MD-enriched enhancers (C1) that were activated during tumor progression (Fig. 4g, h). In the same setting, we also measured a decrease of SOX9 binding at enhancers that were decommissioned in MD cells (C3-C4), suggesting that this TF plays a modulatory role in enhancer activity, depending on the cellular state or its responsiveness to environmental stimuli. Indeed, by characterizing the SOX9 iCD in metastatic cells, we found that its promoter is engaged in multiple, low frequent interactions with CREs (Fig. 4i), suggesting that the fine tuning of its expression may depend on the integration of the activity of both LDTFs and SDTFs.

### Enhancer connectivity specifies RA-mediated transcriptional memory

**Fig. 5:**
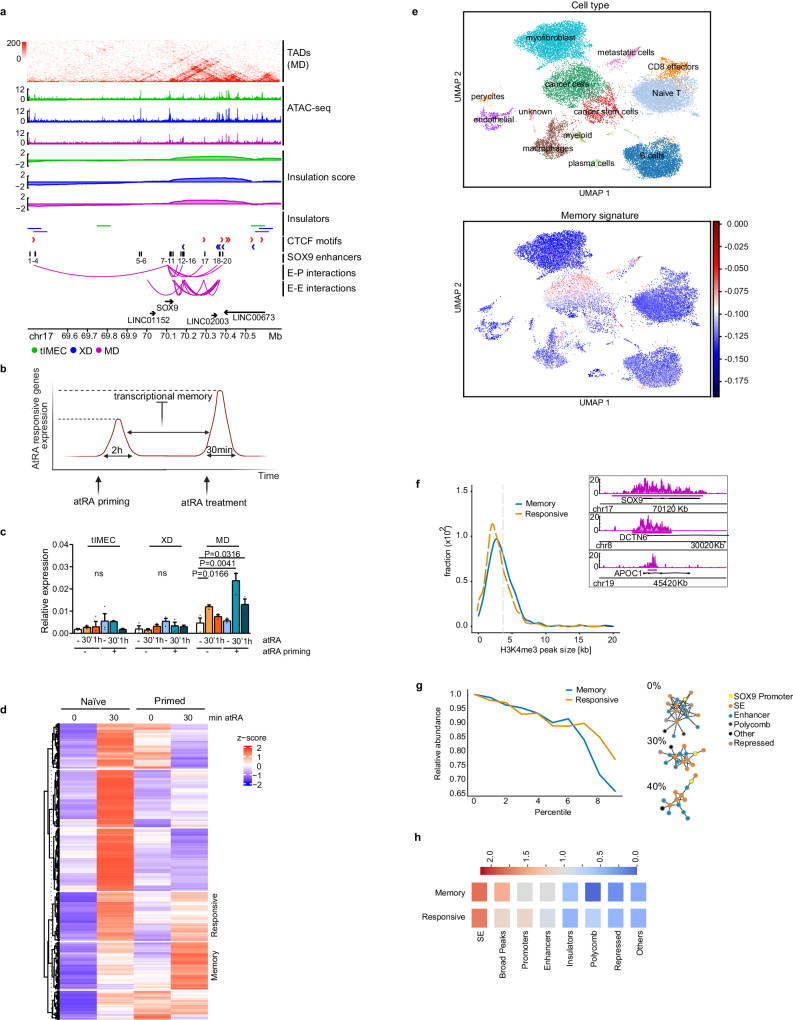
Enhancer redundancy specifies SOX9 transcriptional memory. **a** Snapshot of the genomic locus surrounding the SOX9 (hg19, chr17:69400000-70700000), showing the ATAC-seq signal, the insulation scores, insulator boundaries for tIMEC, XD, and MD. In addition, CTCF sites for TNBC cell lines (HCC1599 and MB157), the 20 enhancers identified by ATAC-seq around the SOX9 locus and H3K27ac HiChIP enhancer-promoter (E-P) and enhancer-enhancer (E-E) interactions in MD. **b** Schematic representation of transcriptional memory in response to atRA. Cells are primed with a 2 h 1 µM atRA pulse, followed by a 36 h chase phase, and then treated again with 1 µM atRA or vehicle for 30 min or 1 h (created with BioRender.com). **c** Barplots of SOX9 relative expression in naïve and primed tIMEC, XD, and MD cells, untreated or stimulated with atRA for 30 or 60 min. Data are means of three independent biological replicates ± S.E.M. **d** Heatmap showing the changes in expression level (z-score of spike-in normalized FPKM values) identified by nascent RNA-seq in three biological replicates. **e** Uniform manifold approximation and projection (UMAP) embedding showing individual cells labeled according to their cell type (left), highlighting the enrichment of memory signature (right); number of analyzed cells: 22158. **f** Breadth distributions of H3K4me3 CUT&RUN signals in MD cells at the TSS of either all analyzed genes (dashed line) or memory genes cluster (blue line). On the top right, three genome browser snapshots are shown for H3K4me3 domains of different sizes from a memory (top), responsive (middle) or a non-responsive (bottom) gene. Window size for all three plots is 10 kb. **g** (Left) Loss of HiChIP regions associated with memory (blue) and responsive (yellow) promoters, upon filtering according to increasing thresholds of connectivity. (Right) Visualization of SOX9 clusters without connectivity filters (top), using a connectivity threshold equal to the 30th percentile of connectivity (middle) and using a connectivity threshold equal to the 40th percentile of connectivity (bottom). **h** Strength of chromatin annotations associated with memory (top) and responsive (bottom) clusters. The value of strength is obtained through the ratio between the fraction of HiChIP regions annotated with a particular chromatin state in non-filtered clusters and in clusters filtered with a threshold corresponding to the 90th percentile of connectivity. Statistical significance was determined by one-tailed unpaired student’s *t*-test.

To gain biological insights into the possible regulatory network eliciting the activity of enhancers in modulating the expression of key pro-metastatic genes, we focused on SOX9, whose deregulation favors cancer dormancy and metastasis formation (Fig. 1h)^11,22,47,48^. SOX9 is located in a gene-poor region at the edge of a chromatin domain in which we detected multiple chromatin loops between its promoter and distal enhancers, whose frequency was augmented in the MD tumoroids (Fig. 5a and Supplementary Fig. 5a). Among the distal CREs, we identified a group of enhancers which were predicted to contain multiple binding sites for retinoic acid receptors (RXR/RAR) and SOX9 itself, suggesting an integrated regulatory loop (Fig. 5a and Supplementary Fig. 5b). To verify the responsiveness of SOX9 to retinoic acid signaling, we treated tIMEC, XD and MD cells with all-trans retinoic acid (atRA) and found that only metastatic cells were responsive, showing an increased SOX9 expression after short-term treatment (Supplementary Fig. 5c). Of importance, the atRA-mediated increase of SOX9 gene expression was not limited to the MD tumoroids, as it was also measured in the D-HEp3 cancer cells, an independent model of quiescence (Supplementary Fig. 5d)^11^. By knocking down RARα, we confirmed that the SOX9 expression is depending on RA-signaling (Supplementary Fig. 5e and Supplementary Data 6).

Based on the observation that treatment with atRA did not maintain long-term over-expression of SOX9 (Supplementary Fig. 5c), we postulated that the epigenetic rewiring of the 3D enhancer network could support the adaptation of MD tumoroids to recurrent RA stimuli, driving an enhanced transcriptional bursting through the establishment of transcriptional memory (Fig. 5b)^16,18,19,49,50^. To test this possibility, we primed MD tumoroids with 2 h treatment of atRA followed by a chasing phase, to then re-stimulate cells before gene expression analyses. We found that tIMEC and XD were not responsive to atRA priming, whereas metastatic tumoroids showed an increased response with an augmented rate of transcription (Fig. 5c). Of importance, the measured transcriptional response resulted independent from the extension of the chasing phase and the cellular model system of quiescence, considering that D-HEp3 showed a similar pattern of SOX9 reactivation upon re-exposure to atRA (Supplementary Fig. 5f, g). This was further confirmed in SUM159PT cells, a pro-metastatic basal cell line, and the poorly metastatic ER+ breast cancer cell line T47D (Supplementary Fig. 5g). Furthermore, other genes involved in the retinoic acid pathway showed RA-mediated transcriptional memory suggesting a shared epigenetic mechanism, possibly involving the CREs (Supplementary Fig. 5h). To verify this hypothesis, we profiled the RA-responsive genes in naïve and primed metastatic tumoroids by measuring nascent transcripts through EU-RNA-seq (Fig. 5d, Supplementary Data 6). Clustering analysis identified 369 genes showing an enhanced response upon treatment with atRA in primed, with respect to naïve MD cells (named thereafter as memory genes) (Fig. 5d). Beside SOX9 and the RA biosynthesis genes, this cluster was enriched for stimuli-responsive genes, including pro-dormancy signaling such as TGFβ and p38^51^, as well as wound healing and cell migration (Supplementary Fig. 5i, Supplementary Data 7). EU-RNA-seq highlighted a group of atRA-responsive genes, which did not show an increased transcriptional rate in the primed MD cells (termed responsive genes). Notably, by analyzing scRNA-seq datasets obtained from breast cancer patients^52^, we found that the signature of memory genes was enriched in the DTCs retrieved from lymph nodes metastasis (Fig. 5e and Supplementary Fig. 5j). Of relevance, the same signature was also depicted in the primary tumors, suggesting that this group of genes were activated in cancer cells before their dissemination to distal sites (Fig. 5e). In addition, we found that cancer cells activating the memory genes were also enriched for the dormancy transcriptional program (Supplementary Fig. 5k), which characterizes early DTCs in breast cancer^15^. We then determined the possible genetic and epigenetic contribution of the CREs to the adaptive mechanisms of transcriptional memory. Genome-wide association studies (GWAS) have associated inherited risk loci with cancer susceptibility, which are enriched at CREs^38^. We found that the distal CREs-linked to the memory genes were enriched for breast cancer susceptibility sites (Supplementary Fig. 5l) and not for pan-cancer SNPs, suggesting a pathological link with breast cancer progression. By analyzing the associated epigenetic state, we noticed that promoters of memory genes were enriched for broad H3K4me3 peaks, which is a chromatin feature characterizing cell identity and cell function genes (Fig. 5f)^53^. By performing cluster analyses on the 3D genome topology of the memory genes, we found that they were organized into iCDs embracing multiple, yet low frequent interconnections, when compared to the responsive genes (Fig. 5g). By querying the robustness of the interactions through sequential removal of the connections based on their relative strength, we measured a sharp decrease of the connections of the memory gene iCDs, with respect to the responsive group (Fig. 5g). By analyzing the chromatin state of the gene clusters (Supplementary Fig. 5m), we found that super-enhancers (SE) and promoters characterized by broad H3K4me3 peaks were enriched among the memory gene iCDs (Fig. 5h). These results suggested that multiple iCD compositions coexist within the MD tumoroids, giving rise to epigenetic plasticity that overall increases the possibility to establish transcriptional memory.

### RA-mediated transcriptional memory relies on enhancer activity

**Fig. 6:**
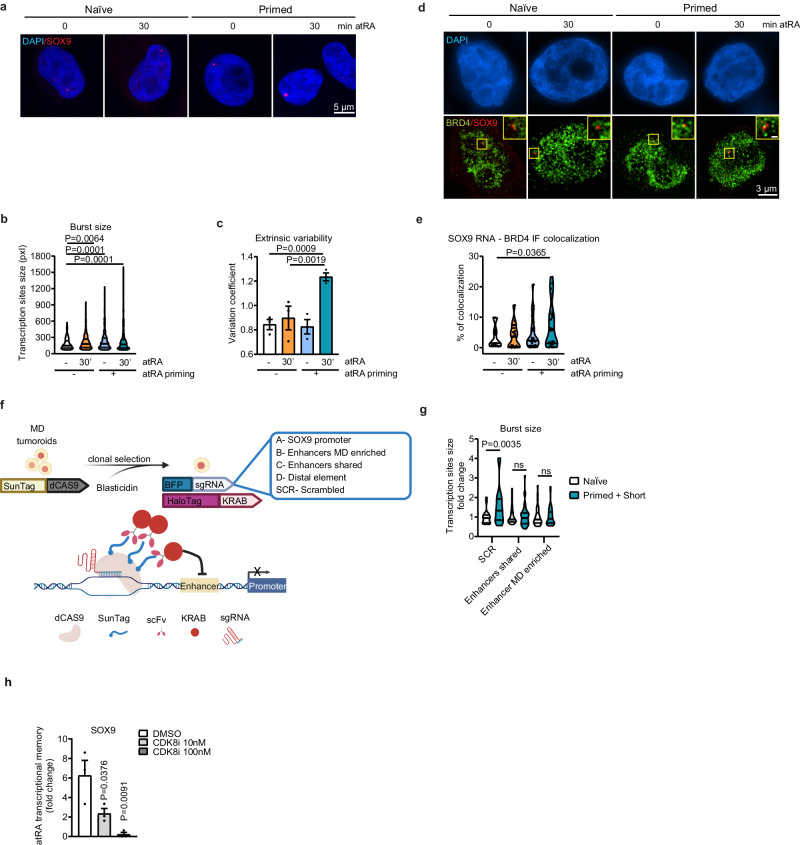
RA-mediated transcriptional memory relies on enhancer activity. **a** Representative images of SOX9 nascent RNA detected by smRNA-FISH in naïve and primed MD cells, after 30 min treatment with vehicle or atRA. Nascent SOX9 RNA, red; DAPI, blue; scale bar = 5 µm. **b** Violin plots showing SOX9 nascent RNA foci area (pxl) in naïve and primed MD cells after treatment with vehicle or atRA. **c** Barplots of variation coefficient (CV) of SOX9 nascent RNA foci size. **d** Representative images of SOX9 nascent RNA detected by smRNA-FISH coupled with BRD4 immunostaining in naïve and primed MD cells after 30 min treatment with vehicle or atRA. DAPI, blue; BRD4, green; nascent SOX9 RNA, red; scale bar = 3 µm; smaller crop scale bar = 0.5 µm. **e** Violin plots showing percentage of colocalization between SOX9 nascent RNA foci and BRD4 immunostaining in naïve or primed MD cells, after treatment with vehicle or atRA. **f** Schematic representation of CRISPRi experimental setup obtained by combining the SunTag-dCAS9 with scFV-KRAB and BFP-sgRNAs targeting MD-enriched enhancers (#13, 14, 15 and 16), enhancers shared among tIMEC, XD and MD cells (#6 and 17), scramble (SCR), SOX9 promoter or a distal unrelated element (created with BioRender.com). **g** Violin plots showing the fold change of SOX9 nascent RNA foci area, between primed cells treated with atRA in respect to naïve condition, upon epigenome silencing of control (SCR), MD-enriched (**b**) or shared Enhancers (**c**). **h** Barplots of SOX9 relative expression levels in MD cells treated with vehicle or CDK8i at 10 nM or 100 nM. Comparison of vehicle and treatments is represented as fold change of expression of the target amplicon in primed cells after 30 min atRA treatment with respect to vehicle-treated MD cells. The barplots in (**c**) and (**h**) are means of three independent biological replicates ± S.E.M. The violin plots in (**b**), (**e**), and (**g**) indicate median values (middle lines), first and third quartiles (dashed lines) retrieved from three (**b**, **e**) or two (**g**) independent biological replicates. Statistical significance was determined by one-tailed unpaired student’s *t*-test (**b**, **c**, **g**, **h**) or one-tailed Mann–Whitney test (**e**).

To experimentally verify this hypothesis, we analyzed the response to atRA treatment in naïve and primed MD cells at single-cell level, by performing smRNA-FISH on the nascent transcript of SOX9 (Fig. 6a, b). We found that while the fraction of SOX9-expressing cells did not change (Supplementary Fig. 6a), the transcription burst size increased in the primed cells after restimulation with atRA (Fig. 6a, b). Of note, we observed cell-to-cell variability in the response of primed MD cells to atRA treatment, as only a fraction of cells showed a remarkable increase in burst kinetics (Fig. 6c and Supplementary Fig. 6b). To further support these results, we measured by 3D SIM microscopy the proximity of the transcriptionally active SOX9 locus with the transcriptional condensates, detected by immunofluorescence of BRD4 (Fig. 6d, e). We found that the augmented transcriptional kinetics in primed MD cells correlated with an increase in colocalization with BRD4 clusters (Fig. 6d, e). These measurements showed that within the MD tumoroids, only certain cells showed augmented proximity of SOX9 transcripts to condensates. To determine the contribution of the enhancer activity in supporting transcriptional memory, we simultaneously silenced some of the MD-enriched CREs by CRISPRi (Fig. 6f). Indeed, given that multiple SOX9 enhancers showed enrichment for RAR/RXR binding motifs (Supplementary Fig. 5a), we targeted at once either two shared or four MD-enriched CREs (Fig. 6f). By analyzing the SOX9 transcript abundance by PrimeFlow, we found that the silencing of the enhancer was sufficient to reduce *SOX9* expression level in primed MD cells after treatment with atRA (Supplementary Fig. 6c). To define whether the enhancer activity was also contributing to transcriptional bursting, we measured the relative changes of nascent SOX9 transcript by smRNA-FISH. We found that while the frequency of transcriptionally proficient primed cells did not change substantially, the epigenetic silencing of CREs affected the fraction of atRA-responsive cells (Fig. 6g). Indeed, in this condition we observed a decrease in the frequency of p27 positive cells upon re-exposure of primed tumoroids to RA (Supplementary Fig. 6d).

To rule out the mechanism involved in transcriptional memory, we investigated key chromatin factors that may contribute to the increased response in the primed cells^50^. We found that the Mediator kinase module (MKM) sustained atRA-dependent transcriptional memory, as the CDK8/CDK19 inhibition halted the sustained expression of memory genes, including SOX9 (Fig. 6h and Supplementary Fig. 6e). To investigate the direct contribution of MKM in supporting transcriptional memory, we measured the chromatin binding of MED12 on the SOX9 CREs. ChIP assays showed that while the core Mediator component MED1 was similarly bound to the enhancers and promoter, irrespective of the cellular conditions or the atRA treatment, at certain enhancers MED12 binding was increased in primed cells upon atRA restimulation (Supplementary Fig. 6f). These results indicated that MKM is maintained associated to some of the metastatic-enriched enhancers after atRA priming, suggesting that their activity is necessary to support SOX9 transcriptional memory. Indeed, we found that epigenetic silencing by CRISPRi of individual enhancers bound by MED12 halted the atRA-driven transcriptional memory (Supplementary Fig. 6g). To determine the biological relevance of the CDK8/19-dependent transcriptional memory, we quantified whether its inhibition could affect the memory-associated increment in quiescent cells. The obtained data showed that the inhibition of CDK8/19 kinase activity halted the atRA-dependent quiescence in metastatic tumoroids (Supplementary Fig. 6g). In sum, these results suggest that SOX9 distal CREs participate in sustaining RA-mediated transcriptional memory.

### SOX9-dependent quiescence favors the escape of MD from the NK immune surveillance

**Fig. 7:**
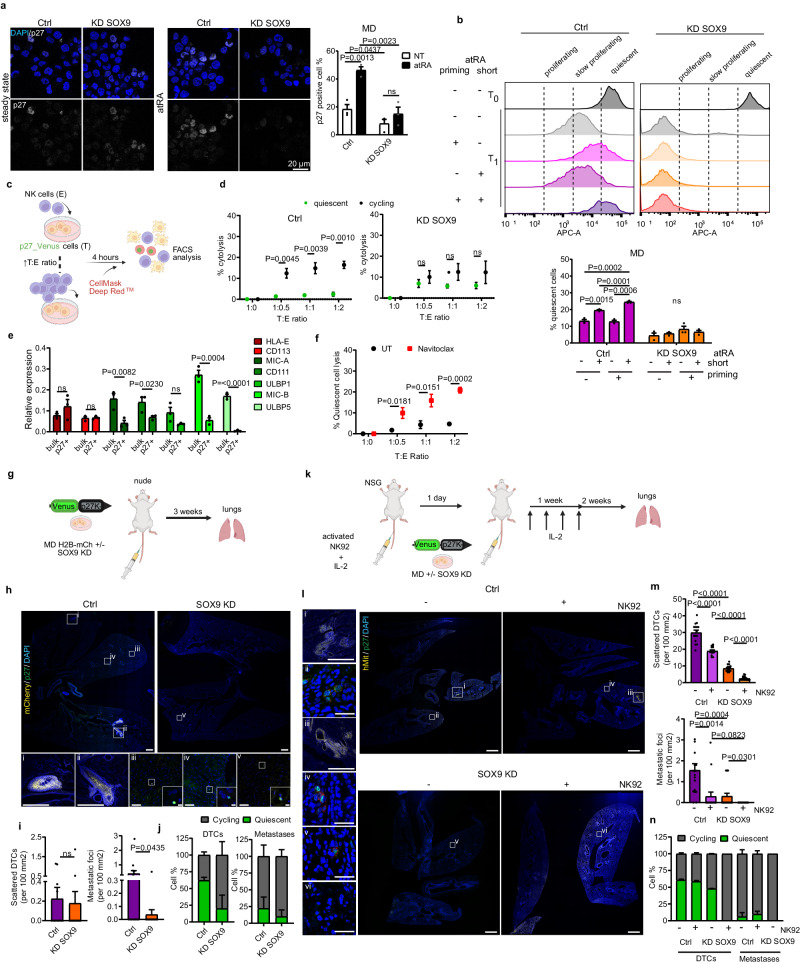
SOX9 drives metastatic-specific cell dormancy through RA-response. **a** Representative images and quantification of p27 positive cells in MD expressing shGFP (Ctrl) or shSOX9 (KD SOX9) in steady state or after 72 h treatment with atRA. p27, white; DAPI, blue; scale bar = 20 µm. **b** Representative histograms of FACS analysis of Ctrl MD and KD SOX9 MD cells showing CellVue Maroon staining intensity (APC-A) 8 days (T1) or just after staining (T0), in the indicated conditions; barplot of the percentage of quiescent cell is shown. **c** Schematic representation of the experimental setup for NK cytotoxicity assays. (created with BioRender.com). **d** Average percentage of NK-mediated cytotoxicity of cycling and quiescent MD cells expressing either shGFP (Ctrl) or shSOX9 (KD SOX9), for different effector:target (E:T) ratios. **e** Barplots of relative gene expression levels of NK activating (green) and inhibitory (red) ligands in bulk and quiescent (p27 + ) MD cells. **f** Average percentage of NK-mediated cytotoxicity of quiescent MD cells for different effector:target (E:T) ratios, after vehicle or Navitoclax treatments. **g** Schematic representation of the experimental setup for metastatic colonization assay (created with BioRender.com). **h** DTCs and metastatic foci distribution within the lungs of nude mice injected with Ctrl or KD SOX9 MD cells. Top, tile scans of entire lung sections; scale bar: 1 mm. Bottom, representative images of metastatic foci (corresponding to i and ii from top image; scale bar = 1 mm) and quiescent DTCs (corresponding to iii-v from top image; scale bar = 50 µm; further zoom scale bar = 10 µm). mCherry: yellow; p27_Venus: green; DAPI: blue. **i** Barplots of scattered DTCs and metastatic foci in lungs of mice injected with Ctrl or KD SOX9, normalized to the lung area analyzed (*n* = 16; data combined from two experimental groups). **j** Barplots of the percentages of cycling or quiescent cells retrieved as scattered DTCs or lung metastases in nude mice (*n* = 16; data combined from two experimental groups). **k** Schematic representation of the experimental setup for metastatic colonization assay in humanized mouse models. (created with BioRender.com) **l** DTCs and metastatic foci distribution within the lungs of NSG mice injected with Ctrl or KD SOX9 MD cells and with or without NK-92 cells. Right, tile scans of entire lung sections; scale bar = 1 mm. Left, representative images of metastatic foci (corresponding to i and iii from top image; scale bar = 1 mm) and quiescent DTCs (corresponding to ii, iv, v, and v from top image; scale bar = 50 µm). Human mitochondria: yellow; p27_Venus: green; DAPI: blue. **m** Barplot of scattered DTCs and metastatic foci normalized to the lung lobe area analyzed in lungs of NSG mice injected with Ctrl or KD SOX9 MD cells, and with or without NK-92 cells, (*n* = 15; data combine from three experimental groups). **n** Barplots of the percentages of cycling or quiescent cells retrieved as scattered DTCs and metastatic foci in the lungs of NGS mice (*n* = 15; data combined from three independent experimental groups). The barplots in (**a**, **b**, **e**) are means of three independent biological replicates ± S.E.M. The mean values ± S.E.M in (**d**) and (**f**) were retrieved from three independent biological replicates. Statistical significance was determined by one-tailed unpaired student’s *t*-test.

To determine whether the sustained expression of SOX9 could increase the fitness of metastatic cells to overall metastatic burden, we analyzed the effects of its downregulation by knocking down its transcript level (Supplementary Fig. 7a). We found that although treatment with atRA reduced the frequency of Ki67 positive cells, SOX9 knockdown rescued their proliferative capacity (Supplementary Fig. 7b). A specular pattern was measured in tIMEC in which the over-expression of SOX9 decreased the fraction of Ki67 positive cells, which was further diminished upon atRA treatment (Supplementary Fig. 7c). Of note, the diminished proliferative capacity in metastatic tumoroids was mirrored by an increment of quiescence, as determined by the increase of p27 positive cells^54^ (Fig. 7a). This finding was corroborated by the concomitant increase of active p38 signaling, a key regulatory pathway supporting cancer cell dormancy^11^ (Supplementary Fig. 7d). Thereafter, we investigated whether RA-signaling could prime metastatic cells toward quiescence, depending on SOX9 expression. Quantification of p27 positive cells and dye retention assay showed that re-exposure to atRA increased the frequency of quiescence in primed cells in a SOX9-dependent manner (Fig. 7a, b and Supplementary Fig. 7e, f). Of note, colocalization analyses showed that the atRA-responsive cells (p27 positive) expressed high level of SOX9 (Supplementary Fig. 7g).

Based on the finding that RA-mediated transcriptional memory modulated metastatic cell quiescence, we re-analyzed the transcriptional response of metastatic tumoroids to atRA stimulation. Clustering analysis of EU-RNA-seq datasets classified a group of genes whose transcript levels resulted to be further diminished in primed cells upon re-exposure to atRA (Supplementary Fig. 7h and Supplementary Data 8). GO ontology indicated that this cluster resulted to be enriched for NK cell receptors such as NKG2D ligands and HLA class I genes, as well as positive regulators of the Wnt signaling pathway (Supplementary Fig. 7i, j, and Supplementary Data 9). As the modulation of these signaling cascades were previously shown to support an immune evasive state^22,48^, we tested whether quiescent MD cells were resistant to NK-mediated cytotoxicity. To this end, we employed the use of the p27 biosensor (mVenus-p27K-) for the identification of quiescent cells (Supplementary Fig. 8a–d)^54^ and measured the frequency of cytolysis upon co-culture with IL-2-activated NK cells (Fig. 7c, d). We found that while cycling metastatic cells underwent cytolysis in a NK concentration-dependent manner, quiescent cells were resistant to NK-mediated cytotoxicity. In addition, gene expression analyses showed that quiescent metastatic cells expressed lower levels of NK activating ligands, suggesting a reduced engagement with NK cells (Fig. 7e). Of importance, we showed that the knockdown of SOX9 increased the responsiveness of quiescent cells to NK killing (Fig. 7d). A similar response was retrieved upon the epigenetic silencing of SOX9 enhancers by CRISPRi, and by the inhibition of the MKM (Supplementary Fig. 8e, f). On the basis of these results, we investigated whether RA-mediated priming of metastatic cells could further elicit their resistance to NK cytotoxicity. We found that re-exposure to RA stimulation reduced the frequency of cytolysis of MD, D-Hep3, T47D or SUM159PT cells upon contacting NK cells (Supplementary Fig. 8g, h). Considering that cancer cell susceptibility to NK killing depends on the mitochondrial priming state to efficiently trigger apoptosis^55^, we investigated whether we could elicit the vulnerability of the quiescent metastatic cells by treating them with the BH3 mimetic Navitoclax. Indeed, gene expression analyses showed that negative regulators of apoptosis were enriched among the atRA-dependent memory genes, irrespective of the metastatic model system used (Supplementary Fig. 5i and Supplementary Fig. 9a, b). Furthermore, we found that quiescent Venus-P27K- positive metastatic cells expressed higher levels of the anti-apoptotic genes BCL-2, and BCLX_l_, which are specifically inhibited by Navitoclax (Supplementary Fig. 9c–e). After determining the critical concentration to prime MD cells (Supplementary Fig. 9f) and confirming the lower mitochondrial priming in quiescent cells upon engaging with NK cells (Supplementary Fig. 9g), we found that Navitoclax treatment specifically increased the responsiveness of quiescent cells to NK-mediated cytotoxicity (Fig. 7f and Supplementary Fig. 9h, i).

We finally tested the contribution of SOX9 to support the escape from NK-mediated immune surveillance in vivo by eliciting dormancy of DTCs. By exploiting an experimental model of metastasis, we intravenously injected MD cells expressing the Venus-p27K- biosensor and shRNA vectors into adult *FOXN1*^*nu/nu*^ mice (Fig. 7g). By analyzing the distribution of MD cells in the lung 21 days post-injection, we found that they colonized distal organs as single and small clusters (<10 cells) of DTCs or gave rise to micro-metastasis, indicating the coexistence of different disease states (Fig. 7h, i). Of note, we found that most of the scattered DTCs were quiescent, as determined based on the frequency of Venus-positive cells (Fig. 7j). However, at the time of the analyses we retrieved that micro-metastasis were characterized by actively proliferating cells, thus indicating that the combination of cell-autonomous and environmental signals defines the balancing between dormant and proliferative states of metastatic cells. We found that the knockdown of SOX9 decreased the frequency of metastatic foci concomitantly to a reduction of the quiescent DTCs (Fig. 7i, j). To investigate the contribution of the enhancer-mediated transcriptional memory of SOX9 in this setting, we silenced the MD-enriched CREs by CRISPRi. We showed that their activation was necessary to maintain the pool of quiescent DTCs capable of evading NK surveillance (Supplementary Fig. 9j–l). These findings suggested that the SOX9-mediated unbalancing of the quiescent state exposed pro-metastatic cells to NK cytotoxicity. To rule out this possibility, we adopted a humanized mouse model by reconstituting the immune-deficient NOD-*scid*IL2Rg^null^ (NSG) mice with human NK cells (Fig. 7k), which were stimulated by IL-2 inoculation every other day for a week. Injection of MD^Venus-p27K-^ cells via an intravenous route resulted in lung micro-metastasis, whose relative abundance was quenched by the adoptive transfer of NK cells (Fig. 7l, m). Indeed, we found that injection of NK cells prevented the accumulation of proliferating metastatic cells, while quiescent DTCs resisted NK-mediated immune surveillance (Fig. 7l–n). This pattern was altered upon the silencing of SOX9, which favored the hyperproliferative capacity of the metastatic cells resulting in an increased sensitivity to NK cytotoxicity, forming less metastatic foci.

## Discussion

Cancer dormancy results from the balancing between the intrinsic and extrinsic signals that constrains DTC’s proliferative potential, concomitantly with the immune-mediated clearance that limits their metastatic outbreak. Albeit their relevance, the cell-autonomous mechanisms that permit DTCs to adapt to these unfavorable conditions have been poorly defined. Here we present evidence indicating that the epigenetic rewiring of distal CREs contributes to DTCs phenotypic plasticity. We found that the metastatic-enriched CREs participate in sustaining transcriptional memory in response to atRA restimulation, leading to a quiescent state of DTCs that facilitate escape from NK-mediated immune surveillance. Given the pro-metastatic function of cancer dormancy, we propose that these distal CREs act as oncogenic enhancers by means of supporting transcriptional memory that increases the fitness of DTCs to eventually give rise to metastasis once favorable environmental conditions would permit their re-awakening.

By integrating chromatin accessibility profiling with chromatin loop mapping by HiChIP we found that memory genes are characterized by large iCDs, encompassing multiple, yet variable CREs interactions, which per se do not correlate with the steady state of gene expression. These findings could be explained considering that enhancer’s activity results from the cooperative action of multiple TFs, some of which are regulated by signaling cascades, which determine their spatiotemporal control on gene expression^29–31,35,36^. Graph-based network analyses predicted that the iCDs of memory genes support transcriptional memory by providing multiple chromatin conformations over time, thereby raising the frequency of E-P interactions that can increase transcription kinetics upon re-exposure to a certain stimulus. The extensive heterogeneity of E-P interactions^56,57^, combined with the stochastic nature of transcription bursting measured at single-cell level by smRNA-FISH, suggest that only a fraction of the primed DTCs activate a robust response upon restimulation with atRA. Nevertheless, the finding that the interactions of MD-enriched enhancers contribute to the iCDs, and their epigenetic silencing restrained the transcriptional memory suggests that distal CREs may act redundantly to increase transcriptional kinetics. We found that the activation of the metastatic-enriched enhancers is supported by RAR/RXR, whose activity depends on the RA-signaling, a developmental pathway that is reactivated in human metastasis^45,46,54^. Of importance, by investigating the contribution of this signaling to gene expression, we found that despite the presence of multiple binding motifs for RAR/RXR within the metastatic-enriched enhancers, atRA stimulation induced a modest increase in transcript levels, while it supported transcriptional memory. These results show that besides ensuring transcription fidelity and robustness, enhancer redundancy can support transcriptional memory by increasing transcription kinetics upon restimulation, in a subset of primed cells. Therefore, we postulate that the combinatorial action of redundant enhancers responsive to RA-signaling combined with the increased frequency of chromatin looping within iCDs determines a permissive 3D chromatin context that supports the establishment of transcriptional memory. However, we can not exclude that additional mechanisms are involved in ensuring an RA-mediated transcriptional memory in metastatic cells. For example, various sources of stresses that activate transcriptional memories have been associated with chromatin changes, including enhancer activation^58^, increased deposition of H3K4me2/3 mark at the promoters^50^, and cohesin-mediated chromatin looping^59^. In line with these observations, we observed a correlation between transcriptional memory and H3K4me3 broad peaks at the promoters, but to what extent and the mechanism by which this chromatin state ensures the propagation of transcriptional memory has not been clarified. Moreover, dissecting possible crosstalk between *cis*- and *trans*-factors to sustain self-propagating mechanisms to ensure a more rapid and robust transcriptional activation in response to re-exposure to certain stimuli warrants further investigations^60^.

Despite the molecular insights, functionally transcriptional memory represents an adaptive strategy that permits cells to learn from a previous signal-mediated experience by mounting a robust response once the same cue is encountered again^19^. This adaptive mechanism permits cells to cope with diverse environmental stressors that may represent potential hazards for the organism, thereby requiring the activation of a memory process for a prompt response in case of re-exposition to the same risk^50,61^. This concept is well exemplified by the establishment of the trained immunity in innate immune cells following exposure to pathogens, which leads to mounting an inflammatory memory^62^. More recently, the memory paradigm has also been expanded to long-lived somatic stem cells, which can remember the previous exposition to an inflammatory condition by activating a transcriptional memory to ensure tissue fitness and functionality^16–19^. We postulate that other threatening signals besides inflammation could also activate a transcriptional memory in cancer cells, thereby increasing their fitness. We focused on signaling pathways that could support the dormancy of DTCs as a pro-survival mechanism ensuring escape from NK-mediated immune surveillance. We identified RA-signaling among the most relevant regulators of signal-dependent TFs that sustained quiescence by modulating the activity of metastatic-enriched enhancers. Although the importance of RA-signaling in cancer dormancy has been previously described^11^, we provided mechanistic insights by showing that exposure to RA-primed metastatic cells to establish transcriptional memory, which supported quiescence through the activation of metastatic enhancers. Indeed, we showed that the RA-mediated transcriptional memory of the pro-metastatic TF SOX9 was sustained by the activation of its enhancers. These findings underline the pathological relevance of systemic, pulsatile signals in regulating the metastatic cascade, besides the microenvironment signals and the immune system control. However, mechanisms controlling the metastatic cell response to systemic signals are still poorly defined. Recent findings highlighted that circadian- or hormone-related pathways can influence the metastatic cascade, favoring circulating tumor cell dissemination and metastatic outgrowth^63–65^. The fluctuating nature of these signals, which also includes vitamins and nutrients whose availability is determined by dietary uptake, organ storage, and circulation^66^, implies that metastatic cells are repeatedly exposed to these signals, possibly sustaining a transcriptional memory^61^. Among these, retinoic acid cellular availability depends not only on the dietary intake of the precursor Vitamin A but also on the expression of RA-related metabolic genes which modulate the turnover of RA through the regulatory feedback loop^67^. In this respect, the RA autocrine signaling is established only in cells expressing the RA-related metabolic genes, ensuring an oscillatory exposure to this metabolite in responsive cells. By mimicking this physiological condition ex-vivo, we found that primed metastatic tumoroids established a transcriptional memory upon re-exposure to RA. Paralleling what has been observed occurring during the establishment of the inflammatory memory^19,68^, the RA-memory genes are enriched for modulators of the RA biosynthesis pathway and pro-dormancy signaling, indicating a conserved regulatory mechanism to tune the associated signaling cascades. Although the role of the RA pathway in supporting somatic stem cell quiescence has been already established^69,70^, further studies will be required to dissect the mechanism by which RA transcriptional memory reinforces regulatory networks driving cancer dormancy. Indeed, we found that quiescent cells are refractory to the NK-mediated cytotoxicity in part due to the downregulation of the NK ligands, which resulted in being regulated by RA-signaling through an undefined mechanism. Although these results are in line with previous findings^22,48^, it has not been determined whether the reduced expression of activating ligands is sufficient to explain the reduced sensitivity to NK-mediated cell killing. Indeed, the recognition and engagement of the NK cells is tightly modulated by the balancing of inhibitory and activating signals, which after multiple cellular contacts could lead to cancer cell apoptosis^55^. We found that reducing the pro-apoptotic threshold of cancer cells by treating them with BH3-mimetics increased the sensitivity of quiescent cells to NK-mediated cytotoxicity. These results suggest an alternative route towards increasing NK cell-based immunotherapy by eradicating the quiescent DTCs colonizing distal organs in non-symptomatic cancer patients before metastatic burden. Albeit its therapeutic relevance, further in vivo experiments would be required to confirm the increased sensitivity of quiescent DTCs to NK killing upon treatment with BH3-mimetics to determine the therapeutic window for these drugs and their synergism with NK-mediated immunosurveillance.

## Methods

### Cell lines and tumoroids

All experiments were performed in the following cell lines: hTERT-immortalized human mammary epithelial cells (IMEC) transduced with pMXs-c-Myc, PGK-H2B-mCherry, and pBabe-puro-HA-PIK3CA^H1047R^ (tIMEC)^27^, primary tumor xenograft derived (XD) and metastasis-derived (MD) tumoroids (this work); D-HEp3, were kindly provided by prof. Maria Soledad Sosa’s laboratory T47D, SUM159PT were retrieved from ATCC, and NK-92 cell lines were kindly provided by Dr. Federica Facciotti. Cells were tested for mycoplasma contamination and resulted negative.

### Isolation, derivation, and maintenance of tumoroids

Serial transplantations were performed by orthotopically injection of 2 × 10^6^ tIMEC, suspended in 30 µl of 1:6 Matrigel (BD Biosciences #354230), in the mammary gland of 5-week-old NOD/SCID mice from Charles River Laboratories as previously described^27^. To allow in vivo tracking of tumor and metastasis formation, cells were transduced with a lentiviral vector encoding for Luciferase (pTween-Luc-NOeGFP) and animals were monitored after Luciferin (VivoGlo Luciferin, Promega) injection by using the Photon IMAGER (Biospace Lab). Four weeks after orthotopic transplantation, primary tumors were surgically resected, mice were monitored by bioimaging for further four weeks, and scarified for the isolation of disseminated tumor cells (DTCs) and metastatic cells from the lung. Four independent primary tumors and colonized lungs were chopped into small pieces in sterile conditions, then incubated at 37 °C for 2 h in DMEM/F12 containing 2% bovine serum albumin, 300 U/ml collagenase III (Worthington #M3D14157) and 100 U/ml hyaluronidase (Worthington #P2E13472). Following digestion, tumor cell suspensions were pelleted and then suspended in 0.25% trypsin for 2 min. XD cells obtained from four independent primary tumors were re-injected in the mammary gland of secondary recipient mice. At the end of the experiments, mice were sacrificed according to IACUC guidelines, and tumors and metastasis collected for in vivo imaging, immunohistochemistry, RNA extraction, and cell isolation.

### Cell culture conditions

tIMEC, XD, and MD cells were maintained in suspension by seeding them at 20 × 0^3^ cells/mL and cultured at 37 °C and 5% CO2 in 1:1 DMEM/F12 medium (gibco #11320-074) supplemented with 100 U/mL Penicillin/Streptomycin (gibco # 15140122), insulin (DBA, #I6634), EGF (Clonetics, MEGM SingleQuots #CC-4136), hydrocortisone (Clonetics, MEGM SingleQuots #CC-4136), B-27 Supplement (Gibco # 17504044) and 20 ng/ml human FGF-basic (PeproTech #100-18B). D-HEp3 cells were cultured as previously described^11^ in DMEM cell growth medium (gibco #11965092) supplemented with 10% of fetal bovine serum (PS-FB1, Peak Serum), Sodium Pyruvate (gibco #11360070) and with 100 U/mL Penicillin/Streptomycin (gibco # 15140122). T47D cells were cultured in DMEM cell growth medium (gibco #11965092) supplemented with 10% FBS, Sodium Pyruvate (gibco #11360070) and with 100 U/mL Penicillin/Streptomycin (gibco # 15140122). SUM159PT cells were cultured in RPMI (Life Technologies #21875034) supplemented with 10% FBS, Sodium Pyruvate (gibco #11360070) and with 100 U/mL Penicillin/Streptomycin (gibco # 15140122). NK-92 cells were a gift from F. Facciotti and were cultured at 37 °C in RPMI (Life Technologies #21875034) supplemented with 10% FBS and with 1,000 U ml−1 IL-2 (DBA #200-02). For drug treatments, cells were treated with 1 µM RARα antagonist BMS614 (Tocris #3660), with 1 µM all-trans Retinoic acid (DBA #10-1138) or with ABT-263(Navitoclax) (Aurogene #1001) as indicated in the relative figure legends.

### Animal studies

To model metastatic colonization of breast cancer in immunocompromised mice, 1 × 10^6^ MD cells expressing H2B_mCherry/mVenus-p27K− reporters and transduced with shGFP or shSOX9 constructs, or carrying sgRNA sequences against a scramble sequence or MD-enriched enhancers were injected into the tail vein of 12-week-old female *FOXN1*^*nu*^ female mice in 100 μl of PBS. Mice were perfused after 3 weeks with PBS via the left ventricle and then with 4% PFA; lungs were immediately dissected, collected, and fixed overnight in 4% PFA, left in 30% sucrose for 48 h, and then included in OCT. To model metastatic colonization of breast cancer in immunodeficient mice, 6 weeks old female NOD.Cg-Prkdc^scid^ Il2rg^tm^¹^wjl^/SzJ (NSG) mice (Charles River Laboratories) were intravenously (i.v.) inoculated (day −1) with PBS (vehicle) or 1.5 × 10^6^ NK-92 cells, pretreated O.N. with 300U/ml of IL-2 (#200-02 Peprotech). IL-2 (75kU) was administered the same day of NK cell injection and given every other day for 7 days to promote NK survival. The next day (day 0), 4 × 10^5^ MD cells expressing the H2B_mCherry/mVenus-p27K− reporters and transduced with shGFP or shSOX9 constructs were i.v. inoculated in vehicle and NK-92 injected mice. Metastasis formation was tracked, after the injection of XenoLight D-Luciferin (150 mg/kg, PerkinElmer), on days 14 and 21 by using an IVIS Lumina III In vivo Imaging System (PerkinElmer). After 3 weeks, mice were perfused with 4% PFA and lungs were collected for metastasis and DTCs detection. The mice were maintained in animal room with 12 h light/12 h dark cycles, temperature (22–24 °C), and humidity (40–60%) at CIBIO (Università degli Studi di Trento). All studies on mice were conducted in strict accordance with the institutional guidelines for animal research and approved by the Italian Ministry of Health; Department of Public Health, Animal Health, Nutrition, and Food Safety in accordance to the law on animal experimentation (D.Lgs. 26/2014)., Italian Ministry of Health authorization (IACUC 373/2015-PR). The OPBA Committee of the Ospedale San Raffaele, Milano approved the animal study protocol. The maximal tumor size permitted by the institutional review board was of one centimeter, and we did not reach this limit for any of the studied conditions.

### Transient transfections and generation of stable cell lines

For RARα transient knockdown, MD cells were transiently transfected with 75pmol RARα siRNAs (#4392420 Life Technologies). Transfection was performed using Lipofectamine RNAiMAX transfection reagent (#13778075 Life Technologies) following manufacturer’s instructions. Cells were analyzed 48 h after transfection.

Cells expressing shRNAs against SOX9 or GFP were obtained transducing MD cells with the lentiviral vector pLKO.1 shSOX9 (Addgene #40644) or shGFP (Addgene #110419) at a multiplicity of infection (MOI) of 10.

Cells expressing inducible tetO SOX9 were obtained transducing tIMEC with the lentiviral vector FUW tetO SOX9 (Addgene #41080) at a multiplicity of infection (MOI) of 1.

D-Hep3 cells expressing H2B-mCherry were obtained transducing D-Hep3 cells with the lentivirial vector PGK-H2B-mCherry (Addgene #21217) at a multiplicity of infection (MOI) of 3. Positive cells were isolated through cell sorting with FACS Aria IIu (BD BiosciencE).

Cells expressing mVenus-p27K−were obtained by transfection of PiggyBac_mVenus-p27K^−^ plasmid with CMV_PBase plasmid^71^ in a 5:1 ratio with Lipofectamine 3000 (Life Technologies #L3000001) following manufacturer’s instructions. Positive cells were isolated through cell sorting with FACS Aria IIu (BD BiosciencE). To enrich the population for quiescent cells before sorting, cells were starved with nutrient-depriven medium for 24 h prior to sorting.

### Generation of lentiviruses

Lentiviruses were generated by co-transfection of sub-confluent HEK293T cells with 9 of VSVG, 12.5 μg packaging vector pMDL, 6.25 μg pREV, and 32 μg plasmid DNA of interest through CaCl_2_ transfection. HEK293T medium was changed to growth medium 12 h after transfection and lentivirus was collected 48 h later. Viral supernatants were filtered through a 0.22 mm PVDF syringe filter, concentrated by ultracentrifugation, and stored at −80 °C. Titration of viral particle concentration was evaluated by transduction of HEK293T cells with serial dilution of the viral preparation and assessment of the transduced cell percentage through acquisition at FACS Canto A. Viral titer was calculated as ((transduced cells N)*((positive cell %/100)* (virus dilution)))/ml of medium. Based on the transducing unit (TU)/mL calculated through viral titration, the viral volume used for infections was calculated based on the multiplicity of infection (MOI) as follows: TUtotal = (MOI × Cell Number)/Viral titer (TU/μL).

### Single-molecule RNA FISH and immunofluorescence

Single-molecule fluorescent in situ hybridization for RNA molecules (smRNA-FISH) protocol was adapted from Tsanov^72^. MD cells were seeded on coverslips coated with 0.1% gelatin (Sigma–Aldrich; G1393). Fixation, permeabilization, probe hybridization, and washes have been conducted as previously described^72^. Of note, we adopted an Alexa-647 Fluor 647-labeled secondary probe coupled with 5 unlabeled primary probes covering the first intron of SOX9 gene (Supplementary Data 10). Annealing between the primary and secondary probe set was performed with a ratio of 40 pmol: 35 pmol.

When Single-molecule RNA FISH was coupled with immunofluorescence, glasses were treated as follows: 4% paraformaldehyde for 10 min at room temperature, two washes in PBS 1×, Blocking Solution (0.1% Triton X-100, BSA 2% #126579 Millipore, PBS 1×) for 4–6 h at 4 °C, overnight incubation at 4 °C with primary antibody diluted in the Blocking Solution (BRD4, Abcam - ab128874, 1:200), three washes in cold PBS 1× and incubation with secondary antibody (goat-anti-rabbit coupled to Alexa-488, 1:500) and DAPI for 1 h at room temperature, three washes in PBS 1×. Glasses were mounted in ProLong Gold antifade mounting medium (Thermo Fisher). Images of smRNA-FISH were acquired with a Leica TCS SP8 Confocal microscope (Leica Microsystems) with HCX PL APO ×63/1.40 objective with an additional 3× zoom, using 0.3 μm *Z* stacks at randomly chosen fields. Images of smRNA-FISH coupled with immunofluorescence were acquired with Nikon Eclipse Ti2E N-SIM 3D system with 60 × 1.40-NA oil immersion objective, with an addition 1.5× zoom and ROI 512 × 512, using 0.170 μm Z stacks at randomly chosen fields. Imaging analysis (transcription sites size, percentage of colocalization RNA FISH-IF) were performed using NIS elements (AR 5.11) and the ImageJ’s plugin DiAna.

### Epigenome editing

For CRISPRi enhancer inactivation MD cells were first stably transduced with a lentiviral construct expressing 10xGCN4-dCas9-BlastR (kindly provided by Ron Vale laB), selected with 1 ug/mL blasticidin (InvivoGen) for 10 days and then were used for single-cell cloning in 96-well plate. Single-cell clones were then transduced with a lentiviral construct expressing scFv-KRAB-HaloTag. Single-guide RNA (sgRNA) pairs with distinct U6 promoters were cloned in tandem into the pKLV-U6gRNA(BbsI)-PGKpuro2A-BFP plasmid (kindly provided by Sakari Vanharanta laB) and transduced into cells (sequences provided in Supplementary Data 10). Efficiency of transduction was monitored assessing the expression of HaloTag and BFP markers by fluorescence-activated cell sorting (FACS). Control cells expressed a nontargeting sgRNA pair (scramble).

### Migration assay

2 × 10^3^ cells were seeded in growth factors-deprived medium and placed onto Matrigel-coated (BD Biosciences #354230) transwells of 8-µm pore size (Corning #3422). In the lower part of the transwell, complete medium was placed as a chemo-attractant. The number of migrated cells was calculated up to 72 h by microscope observation: briefly, the upper part of the transwell was cleaned with a cotton swab after 4% PFA fixation of the membrane; then, cells on the lower part of the transwell were stained for DAPI and acquired at SP8 Confocal Microscope with an HC PL APO 20×/0.75 CS2 objective and single Z. Quantification of migration capacity was calculated as the percentage of migrated cells over the number of seeded cells.

### Sphere-forming assay

Cells were seeded in 24 W ultralow attachment plates (CorninG) at a density of 2.5 × 10^3^ viable cells/ml in 2% Matrigel (BD Biosciences #354230). Formed spheres were analyzed after 72 h; images were acquired with ImageXpress Micro Confocal High-Content Imaging System (Molecular Devices) and analyzed using the MetaXpress 6 software (Molecular Devices). Objects were segmented using mCherry fluorescent signal and spheres were defined as round objects with an area '>500 µm^2^. Sphere-forming efficiency (SFE) was calculated as the ratio between the number of spheres per well and the number of seeded cells.

### Invasion assay

Cells were seeded in 24 W ultralow attachment plates (CorninG) at a density of 2.5 × 10^3^ viable cells/ml in 1:1 DMEM/F12 medium supplemented with insulin (Clonetics, MEGM SingleQuots #CC-4136), EGF (Clonetics, MEGM SingleQuots #CC-4136), hydrocortisone

(Clonetics, MEGM SingleQuots #CC-4136), B-27 Supplement (Gibco #17504044), 20 ng/ml human FGF-basic (PeproTech #100-18B) and 2% Matrigel (BD Biosciences #354230). After 72 h from seeding, spheroids were embedded in pepsinized collagen type I from bovine skin diluted in growth factors depleted culture medium (final concentration 1.6 mg ml−1, PureCol, Advanced BioMatrix). Fibrillar collagen matrices containing spheroids were polymerized at 37 °C for 1 h, and then covered with complete culture medium, as chemo-attractant. Invasion capacity of cells was evaluated 72 h after embedding of spheres, by staining with CellTrace™ Calcein Green, AM (Invitrogen #C34852) 1:2000 and acquiring six fields of view per cell line with 50–100 2.6 µm stacks at SP8 Confocal Microscope, HC PL FLUOTAR 10×/0.30 objective with 2× zoom using 20–30 1.5 μm Z stacks. Quantification of the spheroid area and perimeter, the number of disseminated cells, and migration distance was performed with ImageJ. Staining for Col-1 ¾ was performed by permeabilization and blocking of collagen-embedded spheroids with PBS/5% Goat serum (#11475055 Fisher ScientifiC)/1% BSA (#126579 MilliporE)/0.3% Triton X-100 (blocking solution) for one hour at room temperature, followed by incubation with Col-1 ¾ antibody (#0217-050 ImmunoGlobE) 1:100 overnight at 4 °C followed by incubation with a secondary antibody (Alexa-647 Invitrogen) 1:500 for 1 h at room temperature. Quantification of Col-1 ¾ signal was performed by calculating the mean FOV intensity of the average Z-stacks projection followed by normalization of the mean H2B_mCherry signal.

### Immunocytochemistry

Paraffin-embedded sections (5 μm-thick) of mouse lung tissues were exposed to antigen retrieval using heated retrieval solution (pH 6.0) and permeabilized for 10 min on ice with 0.1% Triton X-100 PBS. For immunohistochemistry analysis, sections were incubated overnight at 4 °C with anti-mitochondria antibody (ab92824, Abcam), anti-p27 antibody (#2552, Cell Signaling Technology) and anti-SOX9 antibody (Ab5535, Sigma–Aldrich) and subsequently exposed to biotin/streptavidin (LSAB 2 system-HRP, Agilent) and the 3-amino-9-ethyl carbazole (AEC, Agilent). Nuclei were stained with aqueous hematoxylin (Sigma–Aldrich). Images were obtained using a confocal microscope (Olympus BX60) and quantified with ImageJ - Color Inspector 3D.

### Immunofluorescence

Cells were fixed for 15 min at room temperature with 4% paraformaldehyde (Sigma–Aldrich #158127) and washed three times with PBS, and subsequently trypsinized to disaggregate spheroids. Staining was performed in 96 W U-bottom plates (Corning) according to the following conditions: permeabilization and blocking with PBS/5% Goat serum (#11475055 Fisher Scientific)/1% BSA (#126579 Millipore)/0.3% Triton X-100 (blocking solution) for one hour at room temperature, followed by incubation with primary antibody (Ki67, 1:1000; phospho-p38, 1 µg/mL; SOX9, 1:250; p27-KIP1, 1:800; BMI-1, 1:500; BRD4, 1:500; human Mitochondria, 1:1000; GFP, 1:250) in agitation overnight at 4 °C, three washes in PBS 1X and incubation with secondary antibodies (goat-anti-mouse or -rabbit coupled to Alexa-488 or −647 (Invitrogen), 1:500) and DAPI for 1 h at room temperature. Pelleted cells were then resuspended in 5 µL mounting medium (Fluormount G # 00-4958-02 Life Technologies), spotted on support glasses, and covered with coverslips.

OCT-embedded sections (50 μm-thick) were washed for 20 min in washing solution (0.3% Triton X-100 in PBS 1×), then incubated at room temperature with blocking solution (3% Goat Serum in washing solution) for 1 h. Slides were incubated with primary antibody overnight at 4 °C, washed for 40 min in agitation, incubated with secondary antibody and DAPI for 1 h at room temperature, washed again for 40 min, and finally covered with mounting medium (#PMT030, Histo-Line laboratories) and coverslip. Images were acquired through LAS AF Software 2.6.0 using a Leica SP8 confocal microscope with HCX PL APO 63×/1.40 objective with 2× or 3× additional zoom with single Z. For acquisition of immunostaining of mice tissues, HC PL FLUOTAR 10×/0.30 and HC PL APO 40×/1.30 Oil CS2 with 2× additional zoom were used using 1.5 and 0.5 μm Z stacks for acquisition of the whole 50μm thickness. In cases where image analysis was performed, image acquisition settings were kept constant. In cases where the percentage of signal-positive cells was calculated, this was retrieved through the manual establishment of a threshold on the mean fluorescence intensity bimodal distribution in order to clearly divide the two modes (positive and negative or high and low). Primary antibodies used in this study are reported in Supplementary Data 11.

### Imaging data analysis

Confocal imaging data analyses were performed using ImageJ software. For 2D analysis, DAPI DNA dye was used to identify the nucleus and define the region of interest. The fluorescence intensity and physical parameters were determined. The values of the fluorescence intensity were background subtracted. To quantify the nuclear mean intensity of each staining, LIF files were converted to TIFF multichannel images, and then the following macro for ImageJ was applied:

function DAnalyze(input, output, filename, lothresh, hithresH)

open(input + filenamE);

run(“Duplicate…”, “title = [TO MEASURE] duplicate”);

run(“Duplicate…”, “title = C_nuc duplicate channels = 1”);

run(“Median…”, “radius = 4”);

setAutoThreshold(“Default dark”);

run(“Threshold…”);

setThreshold(lothresh, hithresH);

setOption(“BlackBackground”, truE);

run(“Convert to Mask”);

run(“Fill Holes”);

run(“Set Measurements…”, “area mean integrated skewness area_fraction limit display redirect = [TO MEASURE])

(decimal = 2”);

run(“Analyze Particles…”, “size = size range display clear add”);

close(“Results”);

selectWindow(“TO MEASURE”);

roiManager(“Show None”);

roiManager(“Show All”);

Stack.setChannel(x);

roiManager(“Measure”);

saveAs(“Results”, output + filename + “.csv”);

close();

close();

close();

input = “PathInput/“;

output = “PathOutput/“;

lothresh = lower threshold;

hithresh = higher threshold;

setBatchMode(truE);

list = getFileList(input);

for (i = 0; i < list.length; i + +)

DAnalyze(input, output, list[i], lothresh, hithresH);

setBatchMode(falsE);

The hitresh, lotresh, and size range parameters were determined manually for each set of images.

### FACS analysis

Dye retention assays were performed with CellVue Maroon Cell Labeling Kit (Invitrogen #88-0870-16) on tIMEC, XD, MD, and MD shSOX9 cells following manufacturer’s instructions. After the staining, cells were plated as standard and eventually treated with 1 µM atRA (DBA #10-1138) as indicated in the relative figure legends and acquired at FACS Canto A after four or eight days. Gating for each experiment was performed classifying cells as quiescent, slow proliferating or proliferating based on the intensity of APC-A using FlowJo v10.0 software.

PrimeFlow™ RNA assay (Invitrogen #88-18005) was performed on MD cells following manufacturer’s instructions. Briefly, cells were fixed after dissociation with Fixation buffer 1 for 30 min at 4 °C. Cells were then permeabilized and fixed for 1 h at RT with Fixation buffer 2. Target probe hybridization was performed by incubating the cells for 2 h at 40 °C. Samples were stored overnight at 4 °C in the dark. The following day, pre-amplification and amplification of the signal were achieved by 2 consecutive incubations of 1.5 h at 40 °C with the pre-Amplification solution and the Amplification solution. Finally, cells were incubated with the label probe sets for an hour at 40 °C. Specific target probe sets for SOX9 (#VA1-10452) and GAPDH (#VA4-10641) were purchased from Thermo Fisher Scientific. Cells were acquired by flow cytometry on FACS Canto A and data was analyzed using FlowJo v10.0 software.

The ALDH activity was assessed by the Aldefluor assay kit (Stem Cell Technologies, Inc., Cambridge, MA, USA) according to the manufacturer’s instructions. Single-cell suspensions at a 5 × 10^5^ cells/ mL concentration were incubated with activated ALDEFLUOR reagent for 30 min at 37 °C. Control samples incubated with the inhibitor, DEAB, were used to ensure identification of ALDH^high^ subpopulation. Cells were acquired by flow cytometry on FACS Canto A and data was analyzed using FlowJo v10.0 software.

Assessment of mitochondrial apoptosis in MD mVenus-P27K- was performed by co-culturing MD mVenus-P27K- (target, T) with NK-92 cells (effector, E) in 1:0 or 1:2 T:E ratios for 4 h, followed by cell fixation in 2% PFA for 30 min at 4 °C. Cell permeabilization was performed by incubation in blocking solution (PBS 10% FBS + 1% Saponin) at 4 °C for 30 min, and staining was performed by incubation in blocking solution with primary antibodies for 30 min at 4 °C, three washes in blocking solution and incubation with secondary antibodies (APC-A) in blocking solution for 30 min at 4 °C. Cells were acquired by flow cytometry on FACS Canto A and data was analyzed using FlowJo v10.0 software. Target cells–cells were identified through H2B-mCherry positivity, and BAK/BAX positivity was assessed in quiescent (mVenus-p27K- positive) and proliferative (mVenus-p27K- negative) cell populations.

Staining for BCL-XL,BCL-2, BAK and BAX in MD mVenus-P27K- was performed by cell fixation in 4% PFA for 15 min, followed by cell permeabilization in blocking solution (PBS 10% FBS + 0.5% Triton X) and incubation in blocking solution with primary antibodies (BCL-XL, 1:800; BCL-2, 1:250; BAX, 1:250; BAK, 1:500) for 30 min at 4 °C, three washes in blocking solution and incubation with secondary antibodies (APC-A) in blocking solution for 30 min at 4 °C. Cells were acquired by flow cytometry on FACS Canto A and data was analyzed using FlowJo v10.0 software. BCL-2/BCL-XL positivity was assessed in quiescent (mVenus-p27K- positive) and proliferative (mVenus-p27K- negative) cell populations. Sequential gating/sorting strategies are included in Supplementary Fig. 10.

### Cell viability assay

For Navitoclax treatment cytotoxicity evaluation, MD cells were treated with a range of Navitoclax concentrations (0.01–100 µM) for 24, 16, 8 or 4 h. Cells were then incubated for 4 h at 37 °C after addition of CellTiter Blue reagent (Promega #G8080), and cell viability was analyzed at Varioskan LUX Multimode Microplate Reader (Thermo ScientifiC) through recording the fluorescence at 560(20)Ex/590(10)Em.

### Protein extraction and western blot analysis

Total protein extracts were obtained as follows. Cells were washed twice with cold PBS, harvested by scrapping in 1 ml cold PBS, and centrifuged for 5 min at 250 × *g*. Harvested cell pellets were lysed by the addition of RIPA buffer 30 min at 4 °C. Lysates were cleared by centrifugation for 10 min at 14,000 × *g* at 4 °C and supernatant was collected on ice. Protein concentration of lysates was determined using PierceTM BCA Protein Assay Kit 24 (Thermo Scientific, 574 #23227), according to the manufacturer’s instructions. The absorbance was measured at λ = 595 using Varioskan LUX Multimode Microplate Reader (Thermo ScientifiC). Values were compared to a standard curve obtained from the BSA dilution series.

For western blot analysis, 20 μg of protein samples were boiled and loaded onto 10% polyacrylamide gels and run in Tris-HCl-Glycine pH 8.3 running buffer. After electrophoresis, proteins were transferred to a nitrocellulose membrane. Membranes were blocked in PBS-T containing 5% milk (blocking buffer), for 1 h at RT with constant agitation and incubated with indicated primary antibody O/N at 4 °C with agitation. The membrane was then washed three times with PBS-T, each time for 5 min, followed by incubation with secondary antibody HRP-conjugated for 1 h at RT. ECL reagents (GE Healthcare #RPN2232) was used to initiate the chemiluminescence of HRP. The chemiluminescent signal was captured using the Image Lab 2.0.1 software on LAS3000 system (GE Healthcare).

### NK cell-mediated cytotoxicity assay

Human NK-92 cells were co-cultured with either MD cells transduced with H2B_mCherry/mVenus-p27K− reporters and expressing shCtrl or shSOX9 constructs or with D-Hep3 cells, at different effector:target ratios for 4 h at 37 °C. Following incubation, cells were stained with CellMask^TM^ DeepRed reagent (Life Technologies #C10046) following manufacturer’s instruction and acquired using the BD FACS Diva TM Software on a FACS Canto A flow cytometer and analyzed with FlowJo (BD Biosciences, v.10.5.3). Specific NK cell killing was measured for cycling and quiescent cancer cells as follows:

% specificlysis = % CellMask- (dead) targets − %spontaneous CellMask- targets

For NK cell-mediated cytotoxicity assay following Navitoclax treatment, target cells were treated with ABT-263(Navitoclax) (Aurogene #1001) for 16 h prior to co-culture with NK cells. For NK cell-mediated cytotoxicity assay following atRA treatment, target cells were treated with the indicated combinations of atRA and left to proliferate without treatments for 4 days prior to co-culture with NK cells. For NK cell-mediated cytotoxicity assay following atRA and CDK8i treatment, target cells were treated with the indicated combinations of atRA and 10 nM CDK8i (SEL120-34A, MedChemExpress) and left to proliferate without treatments for 4 days prior to co-culture with NK cells. Sequential gating/sorting strategies are included in Supplementary Fig. 10.

### ATAC sequencing

Four independent biological replicates for each cell population were considered. 50000 cells for each replicate were harvested and washed once with PBS; nuclei were isolated together with homogenized tissues collected from primary breast tumors (PT). Samples were resuspended in 50 μL Tn5 transposase mixture consisting of 1× Tagment DNA Buffer, 0.5 μL Tagment DNA Enzyme (Nextera DNA Library Preparation Kit, Illumina). Transposition reaction was incubated at 37 °C for 45 min, followed by DNA isolation using a Qiagen MinElute PCR purification kit (QIAGEN, Hilden, Germany). Libraries were amplified for 5 cycles using the NEBNext High-Fidelity 2× PCR Master Mix (New England Biolabs, MA, USA) and the dedicated primers. Libraries were purified using Agencourt AMPure XP beads and quantified using the 2100 Bioanalyzer (Agilent cat. #G2939BA) and the Qubit fluorometer (Thermo Fisher cat. #Q33226). Libraries were sequenced on an Illumina HiSeq2500 with SE 50 bp.

### RNA sequencing

Cells were seeded at maintenance density four days before collection. Three independent biological replicates per condition were collected. Cells were directly lysed on plates, and tissue collected from primary breast tumors (PT) was lysed with TRIzol (Thermo Fisher cat. #15596026), and total RNAs were extracted according to the manufacturer’s instructions. Contaminating genomic DNA was removed by DNase (Qiagen cat. #79254) digestion. RNA quality and concentration were assessed using the 2100 Bioanalyzer (Agilent cat. #G2939BA) and the Qubit fluorometer (Thermo Fisher cat. #Q33226), respectively. RNA-seq libraries were prepared by using the TruSeq® Stranded Total RNA (Illumina #20020596) supplemented with Illumina Ribo-Zero plus rRNA Depletion Kit (Illumina #20037135) starting from 500 ng of total RNA. Libraries were sequenced on an Illumina HiSeq2500 with SE 50 bp.

### HiChIP sequencing

HiChIP was performed as described (69), using H3K27ac antibody (Abcam #ab4729), starting from 1 × 10^6^ cells per condition. Digestion and ligation were performed overnight (NEB T4 Ligase, #M0202 instead of Invitrogen T4, 15224-041) and DNA was extracted with phenol-chloroform. After biotin pull-down, beads were resuspended in 25 μL of 2× TD Buffer, 0.5 μL of Tn5 (Illumina #1503861), and water to 50 μL. After transposition, beads were resuspended in 50 µL of Phusion HF 2× (New England Biosciences). Generally, 14 cycles were used for Tn5 Nextera PCR amplification (Illumina Nextera DNA UD Indexes Kit). PCR products were size-selected with 25 μl Ampure XP Beads and eluted in 30 μl of Elution buffer. Finally, libraries were sequenced on an Illumina NovaSeq6000 with PE100pb.

### EU-RNA sequencing

For the analysis of nascent RNA expression, cells retrieved from three independent biological replicates pulse-labeled with 0.5 mM 5-ethynyl uridine (5-EU) for 30 min at 37 °C. After pulse-labeling, total RNA was isolated using TRIzol, and purified RNA was further processed using the Click-iT^TM^ Nascent RNA Capture Kit (Invitrogen, C10365), as described^73^. In brief, 5-EU incorporated RNA was biotinylated, purified, and pulled down using Dynabeads™ MyOne™ Streptavidin T1 beads. Before the pull-down, biotinylated custom-made spike-in RNAs #1 (2.5e-5 ug) and #2 (2.5-e-4 ug) were added to 1 µg of biotinylated RNA. After washing the beads, cDNA was directly generated off the beads using the Universal Plus Total RNA-Seq with NuQuant kit (Tecan) with the following modifications: fragmentation time was increased to 10 min and after second strand synthesis, beads were collected on a magnet, and supernatant was transferred to a new tube for subsequent steps. After quality assessment, libraries were pooled and sequenced on an Illumina NovaSeq6000 with 150 bp PE.

### Calibrated CUT & RUN

For each IP, 2.5 × 10^5^ cells retrieved from three independent biological replicates were collected and mixed with 1 × 10^4^ NIH 3T3 cells for calibrating the CUT&RUN data. After capturing isolated cells on ConcavalinA-coated magnetic beads and digitonin permeabilization (0.025%), samples were incubated with the antibodies listed in Supplementary Data 11. Purified Protein-A-MNase was added to each sample and incubated 30 min at 37 °C, before collecting the supernatant to purify the released DNA by phenol-chloroform extraction. Sequencing libraries were produced using the NEBNext® Ultra™ II DNA Library Prep Kit for Illumina® (E7645) as described by the manufacturer. Libraries were pooled and sequenced on an Illumina NovaSeq6000 with 50 bp PE.

### Data analysis

#### Analysis of the ATAC-seq data

After ATAC-seq data pre-processing through Trimmomatic v0.39, BowTie2, samtools v1.10 and HOMER v4.11 and peak calling with callPeaks hg19 (–peak size 500 –distance 1000 –L 0 –C3), differential accessibility analysis was undertaken using the edgeR v3.20.9 and limma v3.34.9 software packages. Normalization factors were calculated with calcNormFactors using the TMM. Differential accessibility between all cell types was assessed using the quasi-likelihood (QL) framework of the edgeR package. *P*-values were adjusted for multiple testing using the Benjamini-Hochberg method. Peaks with a FDR below 0.1% and absolute fold change > 0.5 were defined as differentially accessible regions. Peaks were annotated using the assign ChromosomeRegion function in the ChiPpeakAnno (v 3.18.2’) package in R. Chromatin states were annotated using the ChIP-seq-defined ChromHMM states from the Roadmap Epigenomics project for the ‘E028 Breast variant Human Mammary Epithelial Cells’.

### Published data analysis

Using the umap function in the umap R package (v0.2.7.0), tIMEC, XD, and MD accessibility data were projected along with publicly available accessibility data on primary human tumors (Cancer Genome Atlas (TCGA)), and human primary non-cancerous tissues from different locations (GSE165659). In brief, we downloaded the TCGA_ATAC_PanCan_Raw_Counts file containing chromatin accessibility profiles of 410 tumor samples spanning 23 cancer types from The Cancer Genome Atlas as well as the pan-cancer peak calls list (TCGA_ATAC_PanCancer_PeakSet) containing the hg38 coordinates of a list of 562,709 transposase-accessible DNA elements from the GDC. GSE165659 accessibility datasets were downloaded from Gene Expression Omnibus (GEO) and were pre-processed as per above. Similarly, the sci-ATAC-seq data of human adult tissues^40^ were retrieved from GSE184462. The counts matrix was then normalized using the cpm function of the edgeR (v3.26.8) package followed by a quantile normalization using normalize.quantiles function in preprocessCore (1.46.0) package in R. For the tIMEC, XD, and MD the hg19 coordinates were first converted to hg38 using liftOver from liftOver package (v1.8.0) and the hg19ToHg38 liftOver chain in R. For measuring the distance or similarity of chromatin accessibility between the tIMEC, XD and MD samples and each of the other samples in the study we measured the Spearman Rank-order correlation between all samples. Samples were then ranked by their Spearman coefficients in descendant order such that each pairwise comparison was attributed a rank number where smaller the rank number the higher the similarity.

RNA-seq data of TCGA breast cancer samples was downloaded using the XenaBrowser and further sorted for different breast cancer subtypes (LUMINAL A, LUMINAL B, HER2, BASAL). Gene expression per BC subtype was ranked and plotted using R.

ATAC-seq data of TCGA cancer samples (BRCA, COAD, GBM, LGG; KIRC, KIRP)^39^ were downloaded from https://gdc.cancer.gov/about-data/publications/ATAC-seq-AWG. The signal across samples of the same cancer type and for breast cancer for the same cancer subtype was averaged using ‘mean’ of the wiggletools package^74^, converted from hg38 to hg19 using liftOver from the liftOver package (v1.8.0) and the hg38ToHg19 liftOver chain and converted to bedgraph for visualization. To visualize CTCF binding sites in triple-negative breast cancer cells, bigwig files from Petrovic et al., converted to bedgraph and peaks were called as described in the original paper using MACS2^43^. CTCF motifs (derived from IMAGE) were searched in the resulting peaks using FIMO and motifs with a *p*-value < 0.0001 were considered significant.

### RNA-seq analysis

After pre-processing through FastQCand reads alignments through Tophat2, RNA-seq analysis was performed using HOMER. In brief, Tag Directories were created for each of the three replicates per cell type and genes were annotated using analyzeRepeats.pl rna hg19 –rpkm. For differential expression analysis, analyzeRepeats.pl rna hg19 –raw was run to get the raw counts needed as an input for edgeR. Differential expression analyses were undertaken using the edgeR v3.20.9 and limma v3.34.9 software packages. Lowly expressed genes were filtered with the filterByExpr function from edgeR. Compositional differences between libraries were normalized using the trimmed mean of log expression ratios method (TMM), counts were transformed to log2-CPM, and differential expression was assessed. *P*-values were adjusted using the Benjamini and Hochberg method to control the FDR below 0.1%. Genes with an absolute fold change > 0.5 were considered differential. Gene ontology analysis was performed using Enrichr.

### scRNA-seq data analysis

Count matrix was downloaded from Gene Expression Omnibus (GSE158399) and imported into scanpy^75^. Only cells belonging to the primary tumor and to the lymph node positive for metastatic cells were considered. The count matrix was filtered according to Xu et al.^52^, normalized, and log-transformed. Cluster labels were defined according to marker genes’ expression as shown in Xu et al.2. Memory and responsive gene signatures were defined as the 150 genes with the highest expression after the second RA stimulation in the nascent RNA experiment. For each gene-signature of interest (memory genes, responsive genes, and dormancy) the median expression of genes in the set was computed for each cell: we considered this measure as signature enrichment. We then computed the Pearson correlation, to measure the correlation between memory and dormancy signature.

### HiChIP data pre-processing and loop identification

HiChIP sequencing reads were pre-processed, aligned, and filtered using HiC-Pro^76^. We specified the genome assembly (hg19) and the restriction fragment coordinates file for the enzyme used in the processing of the samples (DpnII), while all the other parameters were kept with default values. Filtered reads were then used as input to FitHiChIP^77^ for the identification of interacting regions. We used ATAC-seq ones as reference to improve accuracy of loop calling. Loops were identified at different resolutions (bin sizes of 10 and 25 kb) and we used both peak-to-peak and peak-to-nonpeak loops for background modeling together with the coverage bias regression to assess the significance of identified interactions.

### HiChIP differential analysis, bin hubs, and annotation

To identify changes in 3D conformation between the different samples, we used the script provided by FitHiChIP (DiffAnalysisHiChIP.r) to compare tIMEC vs XD, XD vs MD, and finally tIMEC vs MD. As for the identification step, we used ATAC-seq reads as reference and all parameters were kept with default values. Significant loops (FDR ≤ 0.01) were compared to evaluate the number of loops identified among samples. To this end, we used Unix shell scripting to compare and count the interactions either specific to each sample or identified in two or all three of them. To evaluate the presence of bin hubs in our three samples, we took all bins included in the loops individually and counted the number of times each was included into a significant interaction. To assess whether the significant loops were bringing into special proximity promoter regions with regulatory elements, we annotated each interacting bin. Promoter regions were defined as 2.5 kb upstream up to 100 bp downstream of the transcription start site (TSS), which were obtained from Ensembl (GRCh37 v. 103). Regulatory elements were defined as ATAC peaks that do not overlap a promoter region within a window of 1.5 kb. Bin annotation was then performed using the intersect1D and intersect functions from pgltools.

### CREs-linked gene expression analysis

Starting from HiChIP data of MD cells, a list of chromatin regions was retrieved from the intersection between HiChIP bins and ATAC peaks enriched in MD cells, using bedtools. Each gene whose promoter was comprised in such chromatin regions and their paired bins were selected. The obtained gene list was used to filter the RNA-seq count matrix. Z-scores of filtered counts were plotted with ComplexHeatmap (v.2.10.0)^78^, dividing genes in 3 groups with a consensus k-means clustering approach, using 10 initialization (row_km = 3, row_km_repeats = 10).

### Contact domain boundaries identification

Thanks to the utility function sparseToDense.py implemented in HiC-Pro a dense matrix has been obtained for each HiChIP sample, starting from the relative 10 kb HiC-Pro.ma files, with default parameters. Continuous contact domain boundaries in tIMEC, XD, and MD cells were identified using insulation score (v.1.0.0)^79^, using the parametrization described in Petrovic et al.^43^. Overlap of boundaries between tIMEC, XD, and MD cells were obtained using the R package ChIPpeakAnno (v.4.2), while overlap between tIMEC, XD, and MD cells domain boundaries with MB157 and HCC1599 cells domain boundaries in Petrovic et al.^43^ were defined by bedtools ‘intersect’ command.

### HiChIP degree distribution

The degree of a node represents the number of connections of the node with its neighbors. The degree distribution is the probability distribution of such degrees over the whole network. tIMEC, XD and MD cells have been described through 3 different network representations:

- Promoter-Promoter (P-P) networks: where each node overlap a promoter region
- Enhancer-Enhancer (E-E) networks: in which each node overlaps a H3K27ac peak
- Promoter-Enhancer (P-E) networks: here, each edge connects a Promoter node with an Enhancer node

We compared such distributions using two non parametric statistical tests: the Kruskal–Wallis test was used to assess if samples originate from the same distribution; while, the Mann–Whitney *U* test was used to compare one sample against the other.

### Integration of RNA-seq and HiChIP

A measure of connectivity was retrieved for each gene, whose promoter fell into an HiChIP bin, by adding the amounts of time the promoter bin has been sequenced in a HiChIP pair with any of its neighbors. After that, 10 subsets of genes were identified, corresponding to the 10th−100th percentile of connectivity. After associating genes with their RNA-seq read counts, we computed the probability that a gene of a specific connectivity percentile (perc) was associated with a transcript count higher than the population mean (count>mean), using Bayes theorem:

$$P({count}\, > \, {mean}\,{{{{{\rm{|}}}}}}\,{perc})=P({perc}{{{{{\rm{|}}}}}}{count}\, > \,{mean})P({count}\, > \,{mean})P({perc})$$

### HiChIP network and iCDs generation

Starting from.bed files of HiChIP significant interactions, their network representations were obtained using graph-tool (v.2.45): each HiChIP bin was considered a network node; an edge was placed between two nodes if their relative chromatin regions were involved in a HiChIP significant interaction. The same approach was followed to generate iCD networks by selecting only bins comprised in interconnected chromatin regions.

### Integration of MD-enriched CREs and HiChIP

First, a network representing the interactome has been generated. Second, HiChIP nodes containing at least one MD CRE have been selected, counted, and removed from the generated network. Third, the average network degree was computed with graph-tool.

A permutation approach has been followed to assess if the average network degree was significantly reduced by the removal of bins containing MD-enriched CREs: this consisted in a two-step approach, repeated 1.000 times:

- The same number of nodes containing at least one MD-enriched CREs was randomly selected and removed from the generated network
- The average network degree was then computed and stored

The *p*-value for the average degree obtained without MD-enriched CREs nodes consists in the ratio between the number of permutations, reaching an average degree lower than the average degree of interest over the total number of permutations.

### EU-RNA-seq cluster analysis

To define RA-memory genes, we performed cluster analyses of EU-RNA-seq using a log2fold change > 0.5 of the spike-in normalized FPKM as cut-off (Supplementary Data 6). Starting from the MD cells HiChIP network, an iCD was built for each identified memory promoter. Clusters were generated, computing the nested stochastic block model on the obtained iCDs, providing two layers of information (graph-tool): iCDs connections and sequential genomic order of iCDs’ bins. To filter clusters, we defined thresholds based on HiChIP data. The 10th, to the 90th percentiles were computed for the measures of connectivity: each HiChIP pair is characterized by a value of connectivity, which stands for the number of times that a specific pair has been sequenced. Each of the obtained thresholds was used to filter each cluster: connectivity thresholds acted on edge removal.

### Transcriptional memory network annotation

Only one specific chromatin state was assigned to each bin of each generated network, according to the following hierarchy of annotation:

- SuperEnhancers^80^
- Broad peaks^81^
- Active Promoter
- Active enhancer
- Weak promoter
- Weak enhancer
- Inactive/poised Promoter
- Insulator
- polycomb-repressed
- Heterochromatin
- Others

Annotations from 3 to 11 were retrieved from HMEC ChromHMM annotations, GSE38163.

### Chromatin states enrichment in Memory and Responsive associated genes

To evaluate the enrichment of chromatin states in memory genes compared to responsive-only genes, we computed the fraction of nodes annotated with each chromatin mark for each node of each cluster. We also performed this procedure for clusters filtered using the 90th percentile threshold for connectivity. By the ratio between chromatin marks fraction in filtered clusters and in normal clusters, we evaluated the resilience of each chromatin marks in both genes’ subsets.

In an erosion-like fashion, we computed the fraction of promoters that were not discarded by an increasing connectivity percentile threshold for both memory and responsive gene subset: in particular, we directly counted how many promoters lasted after each filtering threshold, compared to the original number of promoters.

### CUT & RUN data analysis

The function ‘plotHeatmap’ of DeepTools was used to generate heatmaps of CUT&RUN signals centered on differential ATAC-seq peaks. Raw counts of CUT&RUN experiments identified ATAC-seq peaks on differential ATAC-seq peaks were retrieved using HOMER v4.11 and further processed using limma and edgeR to normalize for log2-counts-per-million values for each library (v3.34.9). To identify broad H3K4me3 domains in the MD cells, H3K4me3 peaks were called using MACS2 with the following command: macs2 callpeak -t H3K4me3.bed -c IgG.bed --broad -g hs -p 0.1, using the corresponding IgG sample as control. Resulting peaks were then overlapped with the annotated TSS of the RefSeq-transcript model and merged with the expression data of the corresponding nascent RNA-seq experiment to identify differences in H3K4me3 between different clusters of RA-response genes.

### Transcription factor network analysis - IMAGE

To assess transcription factor activity and identify gene regulatory networks, IMAGE was used^45^. In brief, rpkm-normalized total RNA-seq reads of the three cell lines (tIMEC, XD, and MD) and normalized ATAC-seq reads at either all distal peaks (# 47016) or identified differential peaks (# 7966) were used as input for the analysis. Further analyses were performed using R. Transcription factors were filtered for being expressed in MD, having a higher motif activity in MD compared to XD and tIMEC, and are considered causal with an FDR < 0.1. To construct regulatory networks, target genes of transcription factors selected using the abovementioned criteria of both approaches were filtered to be considered a true target (*P*-value < 0.005), resulting lists were merged and networks were plotted.

### Visualization of the data and plots

Plots were generated using the ggplot2 (v 3.3.3) package in R. For heatmaps, the ComplexHeatmap (v 2.6.2) package in R was used. Venn diagrams and UpSetR plots were generated using the R packages VennDiagram (v 1.7.1) and UpSetR (v 1.4.0). RNA-seq and ATAC-seq coverage plots and bed and bedpe files for HiChIP visualization were plotted using the R package Sushi (v 1.28.0). Multidimensional scaling (MDS) plots were constructed using the plotMDS function in the limma v3.40.6 on the filtered and normalized log2-counts-per-million values for each library^82^. Network degree distributions and connectivity plots as well as heatmaps were generated using Seaborn (v.0.11.2) (Waskom 2021) and matplotlib (v.3.5.2). Networks representations were obtained using graph-tool (v.2.45).

### Enrichment of GWAS loci

GREGOR v1.4.0 was used for enrichment analysis of disease-trait-associated SNPs in the ATAC-seq peaks. GREGOR calculates enrichment relative to MAF, TSS-distance and number of LD neighbor-matched null SNP sets using the GREGOR parameters: *r*^2^ threshold = 0.7, LD window size = 1 Mb, and minimum neighbor number = 500. The GWAS SNP set used for analysis was derived from the NHGRI GWAS catalog. We selected 4329 and 1160 unique SNP IDs associated with cancer and breast cancer, respectively.

### Reporting summary

Further information on research design is available in the Nature Portfolio Reporting Summary linked to this article.

### Supplementary information

Supplementary Information

Peer Review File

Description of Additional Supplementary Files

Supplementary Data 1

Supplementary Data 2

Supplementary Data 3

Supplementary Data 4

Supplementary Data 5

Supplementary Data 6

Supplementary Data 7

Supplementary Data 8

Supplementary Data 9

Supplementary Data 10

Supplementary Data 11

Reporting Summary

### Source data

Source Data

## Data Availability

The RNA sequencing, ATAC-seq, CUT&RUN and HiChIP raw data have been deposited in the Gene Expression Omnibus database under the accession GSE211610. The scRNA-seq datasets were retrieved from the Gene Expression Omnibus database under the accession GSE158399. HiChIP datasets from HCC1599 and MB157 cell lines were retrieved from GSE116876. Accessibility datasets for the different tumor and healthy tissues were retrieved from GSE165659. The remaining data are available within the Article, Supplementary Information or Source Data file. Source data are provided with this paper.

## Code availability

The custom-made code for annotating HiChIP interaction has been deposited in the public repository GitHub: (https://github.com/zippolab/Michelatti_et_al/blob/main/scripts/HiChIP_data_analysis/1_contact_annotations.sh). The custom-made code for integrating MD-enriched CREs and HiChIP has been deposited in the public repository GitHub: (https://github.com/zippolab/Michelatti_et_al/blob/main/scripts/HiChIP_data_analysis/2_MD_CRE_weigth.py). The custom-made code for generating networks from HiChIP data has been deposited in the public repository GitHub: (https://github.com/zippolab/Michelatti_et_al/blob/main/scripts/HiChIP_data_analysis/3_network_generation.py). The custom-made code to perform cluster analysis of HiChIP networks has been deposited in the public repository GitHub: (https://github.com/zippolab/Michelatti_et_al/blob/main/scripts/HiChIP_data_analysis/4_cluster_analysis.py). The custom-made code for the chromatin states enrichment analysis in Memory and Responsive associated genes has been deposited in the public repository GitHub: (https://github.com/zippolab/Michelatti_et_al/blob/main/scripts/HiChIP_data_analysis/5_chromatin_state_analysis.py & https://github.com/zippolab/Michelatti_et_al/blob/main/scripts/HiChIP_data_analysis/6_nascent_promoters_erosion.py). The custom-made code for integrating nascentRNA-seq and HiChIP has been deposited in the public repository GitHub: (https://github.com/zippolab/Michelatti_et_al/blob/main/scripts/HiChIP_data_analysis/7_RNAseq_HiChIP_integration.py). The custom-made code for analyzing scRNA-seq datasets has been deposited in the public repository GitHub: (https://github.com/zippolab/Michelatti_et_al/tree/main/scripts/scRNAseq_data_analysis). All codes can be cited by using 10.5281/zenodo.10593104.
