# Supplementary material for: Oncogenic enhancers prime quiescent metastatic cells to escape NK immune surveillance by eliciting transcriptional memory
Source: Nat Commun. 2024 Mar 19;15:2198. doi: 10.1038/s41467-024-46524-0 (PMC10951355; doi:10.1038/s41467-024-46524-0)

Supplementary Figures

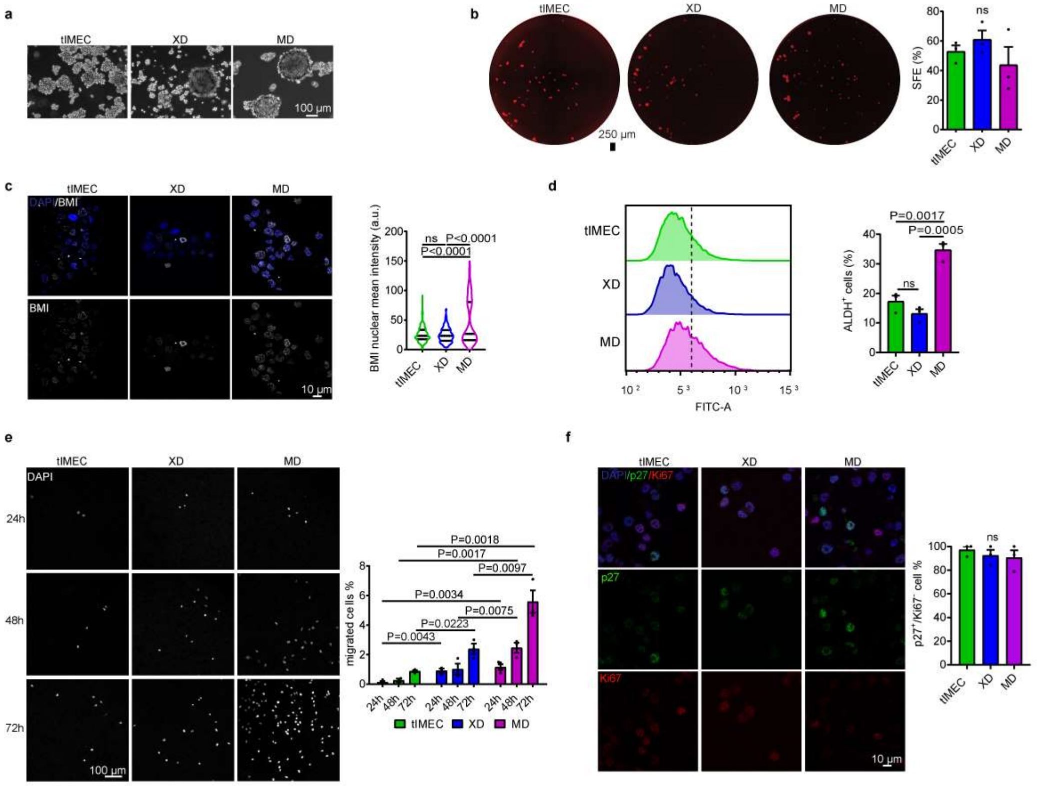

Supplementary Fig. 1: Recapitulating metastatic progression of TNBC ex-vivo

(**a**) Representative brightfield images of tIMEC, XD and MD tumoroids. Scale bar: 100 µm. (**b**) Representative images of tIMEC, XD, and MD tumoroids expressing mCherry-H2B; scale bar: 250 µm. Barplots of sphere-forming efficiency (SFE). (c) Representative images and quantification of BMI1 nuclear mean intensity in tIMEC, XD, and MD cells. BMI1, white; DAPI, blue; scale bar = 10 µm. (d) Representative histograms and quantification of ALDH activity in tIMEC, XD, and MD cells. (e) Representative images and quantification of migration assay in tIMEC, XD, and MD cells at the indicated time points; scale bar: 50 µm. (**f**) Representative images and quantification of p27+/Ki67- cell percentage in tIMEC, XD, and MD cells. p27, green; Ki67, red; DAPI, blue; scale bar = 10 µm. The barplots in b-c, e-f are means of 3 independent biological replicates +/- S.E.M. The violin plots in d indicate median values (middle lines), first and third quartiles (dashed lines) retrieved from three independent biological replicates. Statistical significance was determined by one-tailed unpaired student's t-test.

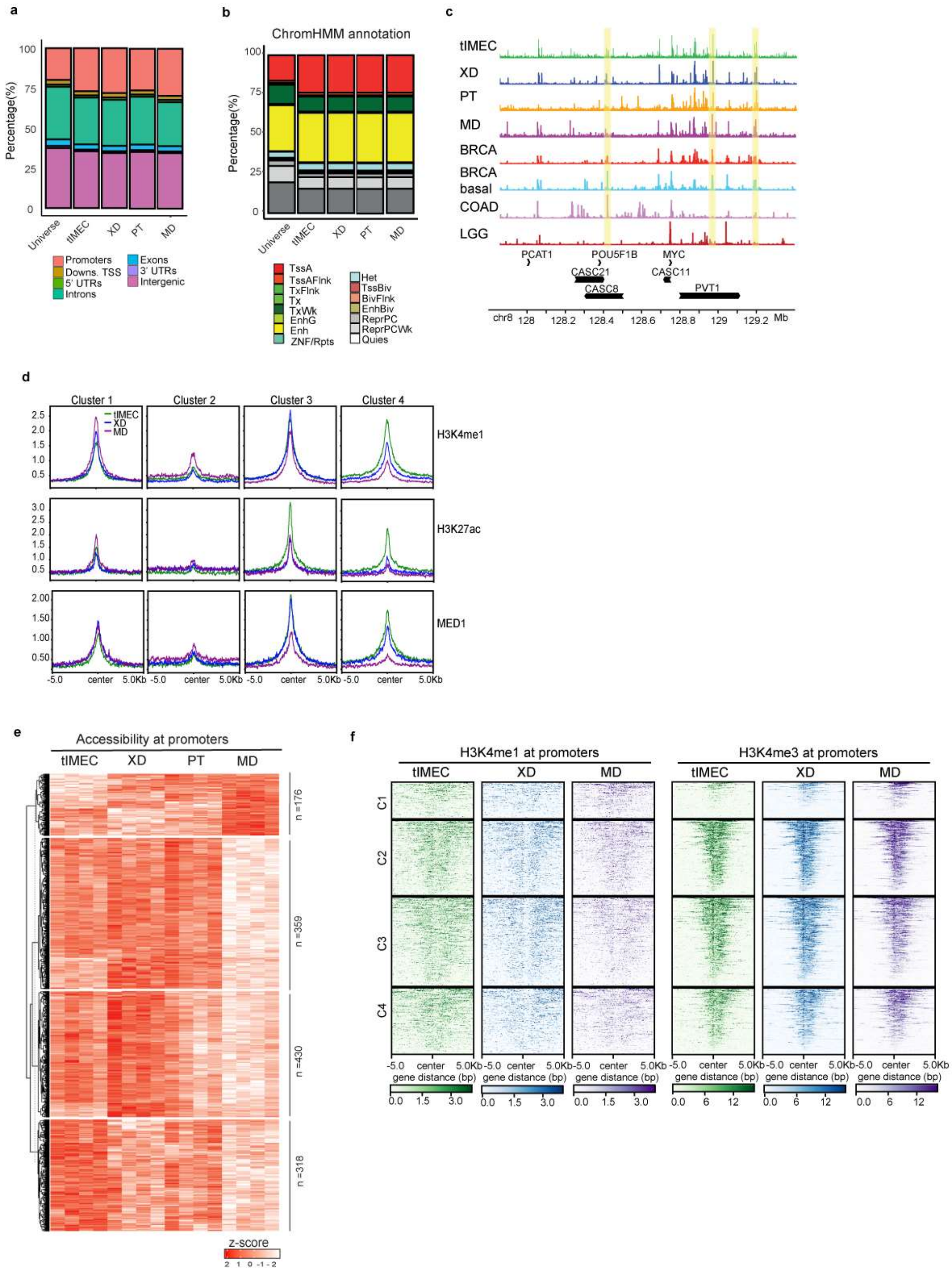

a
Percentage(%)
Universe
tIMEC
XD
PT
MD
Promoters
Downs. TSS
5' UTRs
Introns
Exons
3' UTRs
Intergenic
b
ChromHMM annotation
Percentage(%)
TssA
TssAFlnk
TxFlnk
Tx
TxWk
EnhG
Enh
ZNF/Rpts
Het
TssBiv
BivFlnk
EnhBiv
ReprPC
ReprPCWk
Quies
c
tIMEC
XD
PT
MD
BRCA
BRCA basal
COAD
LGG
PCAT1
POU5F1B
MYC
CASC21
CASC11
CASC8
PVT1
chr8
Mb
d
Cluster 1
Cluster 2
Cluster 3
Cluster 4
H3K4me1
H3K27ac
MED1
-5.0
center
5.0Kb
e
Accessibility at promoters
n =176
n =359
n =430
n =318
z-score
f
H3K4me1 at promoters
H3K4me3 at promoters
C1
C2
C3
C4
gene distance (bp)

Supplementary Fig. 2: Metastatic onset is associated with epigenomic rewiring

(**a**) Barplot showing the distribution of genomic features across peaks identified in tIMEC, XD, MD and PT. (**b**) Barplot showing the distribution of chromHMM annotations across peaks called in tIMEC, XD, MD, and PT. ChromHMM annotation abbreviations are as follows: TssA – active TSS, TssBiv – bivalent/poised TSS, EnhBiv – bivalent enhancer, ReprPC –repressed polycomb, ReprPCWk – weak repressed polycomb, Quies – quiescent/low, TssAFlnk – flanking active TSS, TxFlnk –transcription at gene 5' and 3', Tx – strong transcription, TxWk – weak transcription, EnhG – genic enhancers, Enh – enhancers, ZNF/Rpts – ZNF genes and repeats, Het - heterochromatin. (**c**) Genome browser view showing the ATAC-seq signal for tIMEC, XD, PT, MD, and TCGA breast cancer (BRCA), basal breast cancer (BRCA basal), colorectal adenocarcinoma (COAD) and Low-Grade Glioma (LGG) at the *MYC* locus. Breast cancer-specific peaks are highlighted by vertical yellow bars. (**d**) Cumulative plots of H3K4me1, H3K27ac and MED1 CUT&RUN signals in the four clusters identified by ATAC-seq in tIMEC, XD and MD cells. (**e**) Heatmap showing chromatin accessibility of promoters for the MD vs. tIMEC, MD vs. XD, and tIMEC vs. XD and PT vs XD comparisons (Log2 transformed counts per million, log2CPM) in four biological replicates. 1283 promoters are represented and subdivided in the annotated clusters. Differential accessibility was defined using a log2fold change > 1 and a FDR < 0.1. (**f**) Density plots of H3K4me1 and H3K4me3 CUT&RUN signals at the TSS of the four clusters identified by ATAC-seq in tIMEC, XD and MD cells. Window size 10kb, centered on TSS.

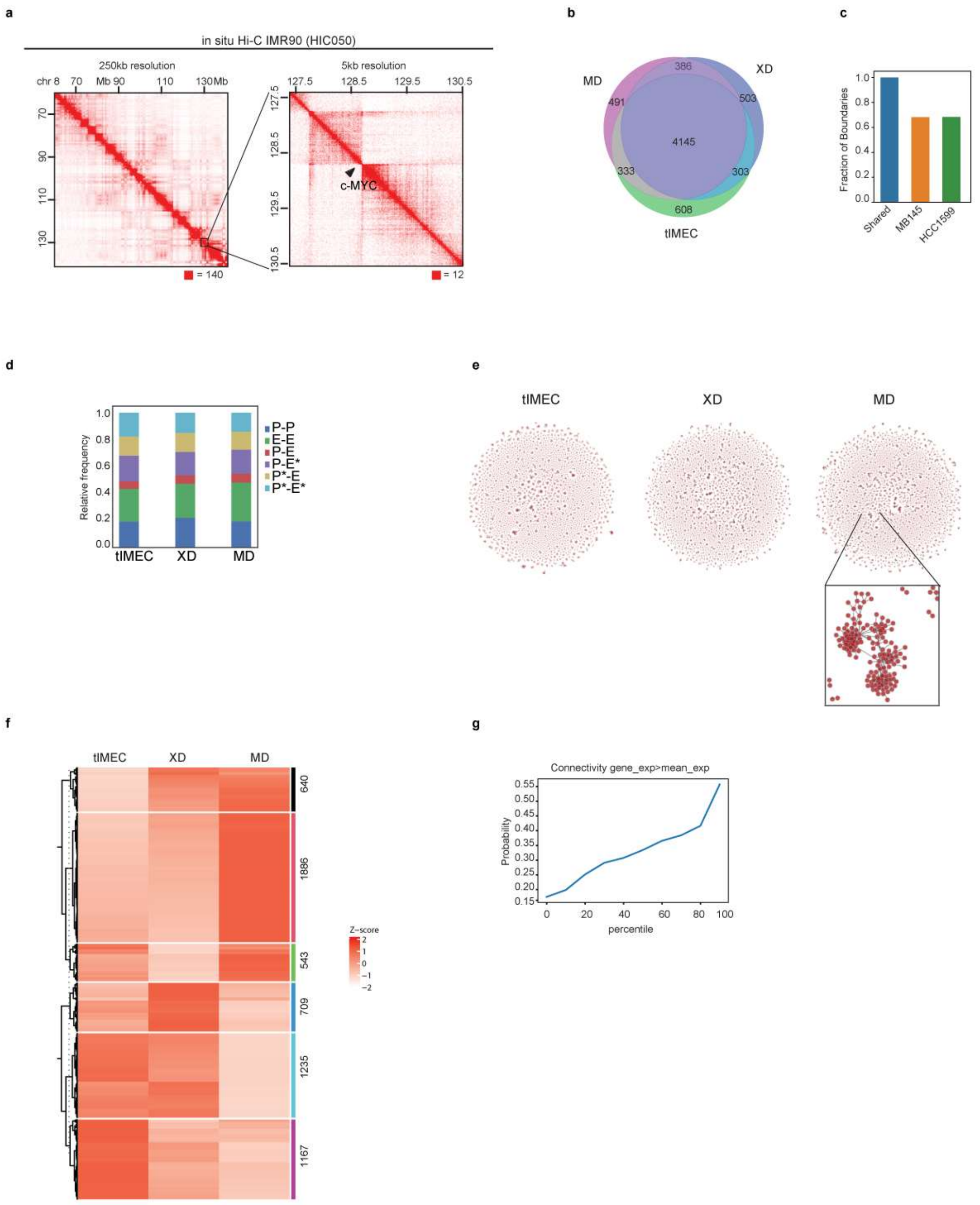

a
in situ Hi-C IMR90 (HIC050)
250kb resolution
5kb resolution
chr 8
70
Mb 90
110
130Mb
127.5
128.5
129.5
130.5
c-MYC
= 140
= 12
b
MD
XD
tIMEC
386
491
503
4145
333
303
608
c
Fraction of Boundaries
Shared
MB145
HCC1599
d
Relative frequency
P-P
E-E
P-E
P-E*
P*-E
P*-E*
tIMEC
XD
MD
e
tIMEC
XD
MD
f
tIMEC
XD
MD
640
1886
543
709
1235
1167
Z-score
g
Connectivity gene_exp>mean_exp
Probability
percentile

Supplementary Fig. 3: Long-range interactions affect chromatin looping during tumour progression

(**a**) In situ Hi-C interaction maps of *MYC* locus in IMR90 cells. 250kb to 5kb resolution (left to right). *MYC* locus (window = 80Mb left, 3 Mb right). Numbers below the interaction maps correspond to the maximum signal in the matrix. (**b**) Visual representation of shared and unique chromatin boundaries. (**c**) Fraction of chromatin boundaries shared between tIMEC, XD and MD cells (schared) and MB145 or HCC1599 cell lines. (**d**) Comparison between tIMEC, XD and MD cells in terms of relative abundance of type of interactions: Promoter-Promoter (P-P), Enhancer-Enhancer (E-E), Promoter-Enhancer (P-E), Promoter-multiple Enhancers (P-E*), multiple Promoters-Enhancers (P*-E), multiple Promoters-multiple Enhancers (P*-E*). (**e**) Network representation of the total HiChIP interactome of tIMEC (left), XD (center), and MD (right) cells. (**f**) Heatmap of RNA-seq transcripts z-scores associated with MD-enriched CREs. (**g**) Distribution plot of the Probability that a gene shows an expression higher than the population mean, given its membership to a connectivity percentile.

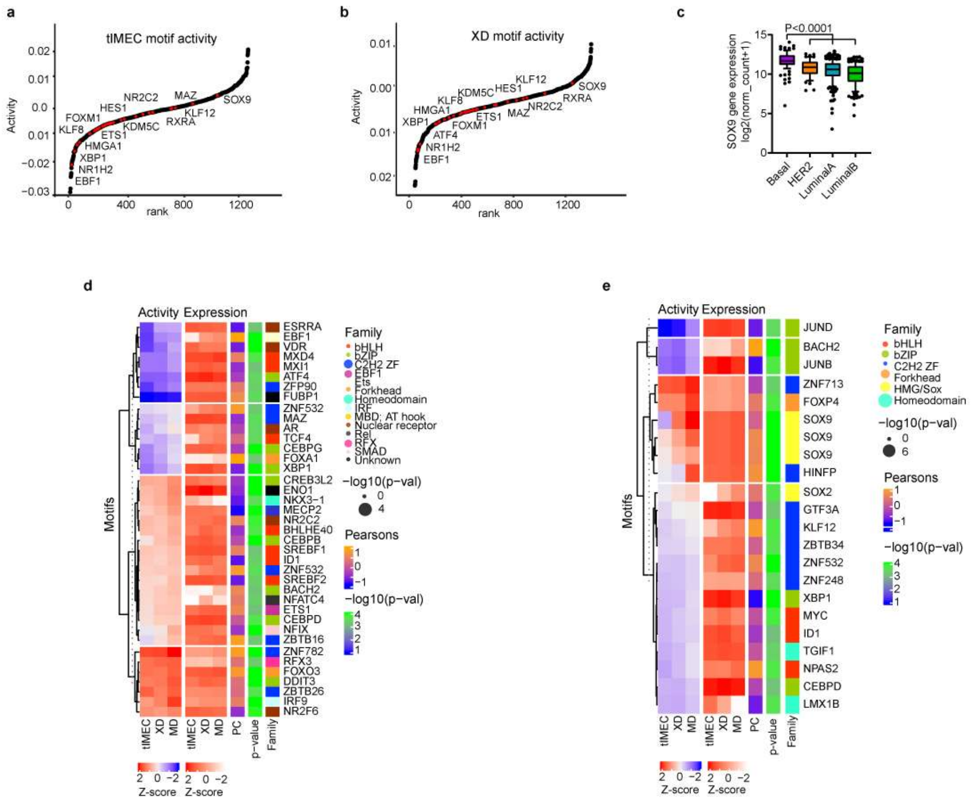

a
tIMEC motif activity
Activity
rank
SOX9
MAZ
NR2C2
HES1
FOXM1
KLF8
ETS1
KDM5C
KLF12
RXRA
HMGA1
XBP1
NR1H2
EBF1
b
XD motif activity
Activity
rank
KLF12
HES1
KDM5C
KLF8
HMGA1
XBP1
ATF4
NR1H2
EBF1
FOXM1
ETS1
MAZ
NR2C2
RXRA
SOX9
c
SOX9 gene expression log2(norm_count+1)
P<0.0001
Basal
HER2
LuminalA
LuminalB
d
Activity
Expression
Motifs
ESRRA
EBF1
VDR
MXD4
MXI1
ATF4
ZFP90
FUBP1
ZNF532
MAZ
AR
TCF4
CEBPG
FOXA1
XBP1
CREB3L2
ENO1
NKX3−1
MECP2
NR2C2
BHLHE40
CEBPB
SREBF1
ID1
ZNF532
SREBF2
BACH2
NFATC4
ETS1
CEBPD
NFIX
ZBTB16
ZNF782
RFX3
FOXO3
DDIT3
ZBTB26
IRF9
NR2F6
tIMEC
XD
MD
PC
p−value
Family
Z-score
Z-score
Family
bHLH
bZIP
C2H2 ZF
EBF1
Ets
Forkhead
Homeodomain
IRF
MBD; AT hook
Nuclear receptor
Rel
RFX
SMAD
Unknown
−log10(p−val)
Pearsons
−log10(p−val)
e
Activity
Expression
Motifs
JUND
BACH2
JUNB
ZNF713
FOXP4
SOX9
SOX9
SOX9
HINFP
SOX2
GTF3A
KLF12
ZBTB34
ZNF532
ZNF248
XBP1
MYC
ID1
TGIF1
NPAS2
CEBPD
LMX1B
tIMEC
XD
MD
PC
p−value
Family
Z-score
Z-score
Family
bHLH
bZIP
C2H2 ZF
Forkhead
HMG/Sox
Homeodomain
−log10(p−val)
Pearsons
−log10(p−val)

Supplementary Fig. 4: TFs contribute to the activation of Metastatic-specific enhancer

Distribution plot showing the rank of the TFs identified as drivers of MD enhancer activity among all TFs considered for the analysis in tIMEC cells (**a**) or in XD cells (**b**). (**c**) Boxplot of gene expression of SOX9 in different breast cancer subtypes (Basal-like, Her2-positive, luminal A and luminal B) derived from TCGA datasets. (**d, e**) Clustering of the top MD-associated motifs in all peaks (**d**) or in the peaks associated with the iCDs identified by HiChIP (**e**), identified in tIMEC, XD and MD cells and retrieved from IMAGE. TF motifs were filtered for a motif activity in MD > tIMEC and XD and further filtered for causality in gene regulation with an FDR < 0.01. Additional columns show z-score normalized expression of motif-associated TF, Pearson correlation coefficient between motif activity and gene expression of associated TF and the associated TF family. Dot size for the TF family indicates enrichment in the unfiltered list by hypergeometric t-test. Dot size is proportional to the log10(p-val). The box plots in c indicate median values (middle lines), first and third quartiles (box edges) and 10th and 90th percentiles (error bars). Statistical significance was determined by one-tailed unpaired student's t-test.

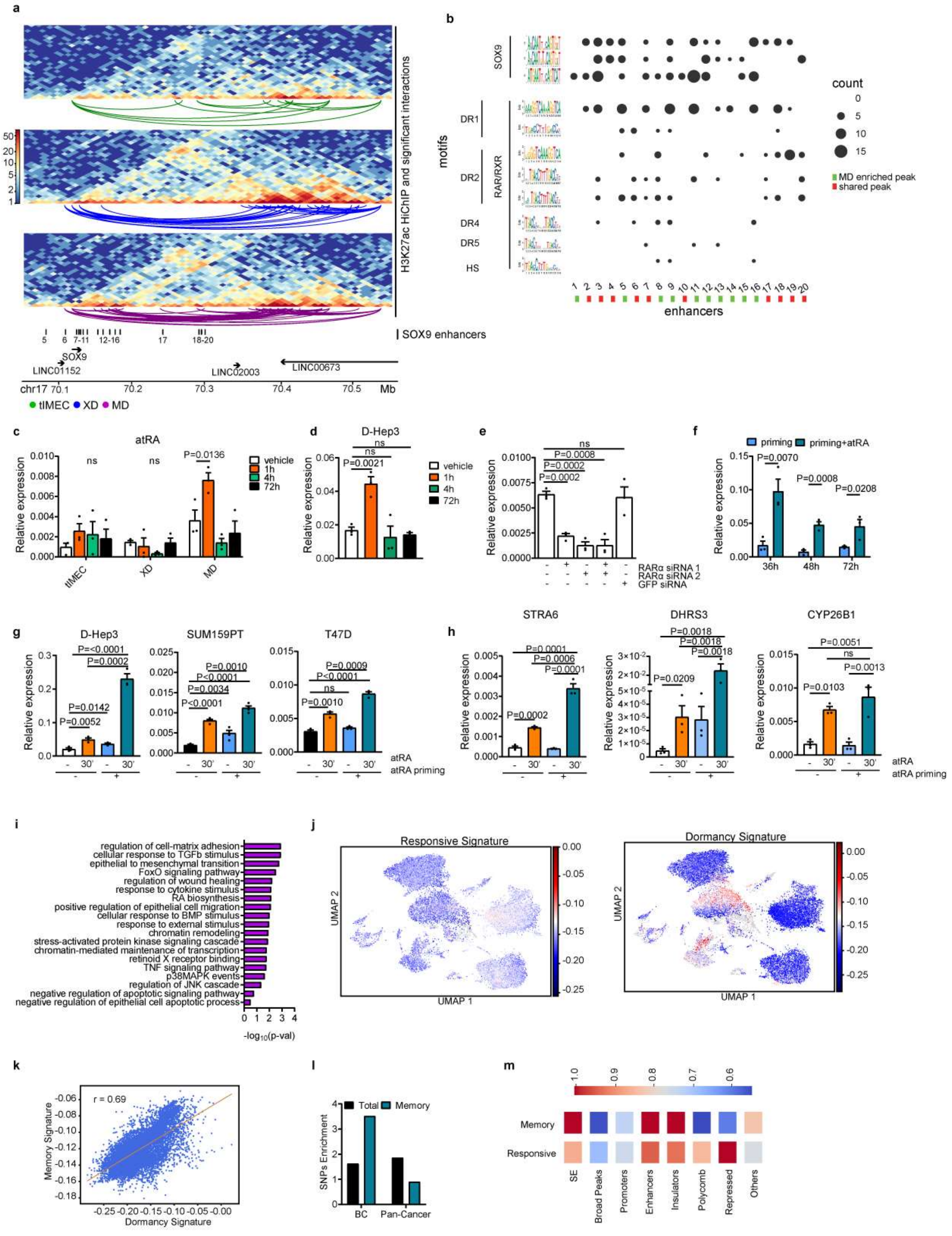

a
H3K27ac HiChIP and significant interactions
SOX9 enhancers
SOX9
LINC01152
LINC02003
LINC00673
chr17
Mb
tIMEC XD MD
b
SOX9
DR1
DR2
DR4
DR5
HS
RAR/RXR
motifs
enhancers
count
MD enriched peak
shared peak
c
atRA
Relative expression
vehicle
1h
4h
72h
tIMEC
XD
MD
P=0.0136
ns
d
D-Hep3
P=0.0021
e
RARα siRNA 1
RARα siRNA 2
GFP siRNA
P=0.0002
P=0.0008
f
priming
priming+atRA
P=0.0070
P=0.0008
P=0.0208
36h
48h
72h
g
D-Hep3
SUM159PT
T47D
atRA
atRA priming
h
STRA6
DHRS3
CYP26B1
i
regulation of cell-matrix adhesion
cellular response to TGFb stimulus
epithelial to mesenchymal transition
FoxO signaling pathway
regulation of wound healing
response to cytokine stimulus
RA biosynthesis
positive regulation of epithelial cell migration
cellular response to BMP stimulus
response to external stimulus
chromatin remodeling
stress-activated protein kinase signaling cascade
chromatin-mediated maintenance of transcription
retinoid X receptor binding
TNF signaling pathway
p38MAPK events
regulation of JNK cascade
negative regulation of apoptotic signaling pathway
negative regulation of epithelial cell apoptotic process
-log10(p-val)
j
Responsive Signature
Dormancy Signature
UMAP 1
UMAP 2
k
r = 0.69
Memory Signature
Dormancy Signature
l
Total
Memory
SNPs Enrichment
BC
Pan-Cancer
m
Memory
Responsive
SE
Broad Peaks
Promoters
Enhancers
Insulators
Polycomb
Repressed
Others

Supplementary Fig. 5: Enhancer redundancy specifies SOX9 transcriptional memory

(**a**) SOX9 locus HiChIP contact matrices generated with pyGenomeTracks of tIMEC, XD and MD cells (from top to bottom) with gene annotation-oriented significant (FDR ≤ 0.01) loops. ATACseq peaks are reported as 'SOX9 enhancers'. Window = 700kb. 10 kb resolution. Color range 1-50 (blue-red) (**b**) Dotplot showing the abundance of RAR/RXR- and SOX9-associated motifs at the identified SOX9 enhancers (1-20). Dot size is proportional to the motif instances found per enhancer. Color blocks under enhancer number indicate whether the ATAC-seq peak is enriched in the MD cells or shared among tIMEC, XD and MD. (**c**) Barplots of SOX9 relative expression level in naïve tIMEC, XD, and MD cells treated for 1, 4, or 72h with atRA. (**d**) Barplots of SOX9 relative expression levels of naïveD-Hep3 cells treated for 1/4/72h with atRA. (**e**) Barplots of SOX9 relative expression levels in MD cells transiently transfected with siRNAs RARa-targeting or with control siRNA. (**f**) Barplots of SOX9 relative expression levels in primed MD cells re-stimulated with atRA after 36, 48 or 72h from the initial atRA treatment. (**g**) Barplots of SOX9 relative expression levels of naïve or primed D-Hep3, SUM159PT or T47D cells after 30 minutes treatment with vehicle or atRA. (**h**) Barplots of STRA6, DHRS3 and CYP26B1 relative expression levels in naïve or primed MD cells after 30 minutes treatment with vehicle or atRA. (**i**) Barplot showing the GO terms associated with genes enriched in the memory cluster and corresponding -log10(p-value). (j) Uniform manifold approximation and projection (UMAP) embedding showing individual cells labeled according to their cell type, highlighting the enrichment of responsive (left) and dormancy signature (right). (k) Correlation analysis between the memory and the dormancy gene expression signatures. (l) Barplot showing SNPs enrichment in distal CREs linked to total or to the memory genes in breast or pan-cancers. (m) Fraction of chromatin annotations associated with memory

(top) and responsive (bottom) clusters. The barplots in c-h are means of 3 biological replicates +/- S.E.M. Statistical significance was determined by one-tailed unpaired student's t-test.

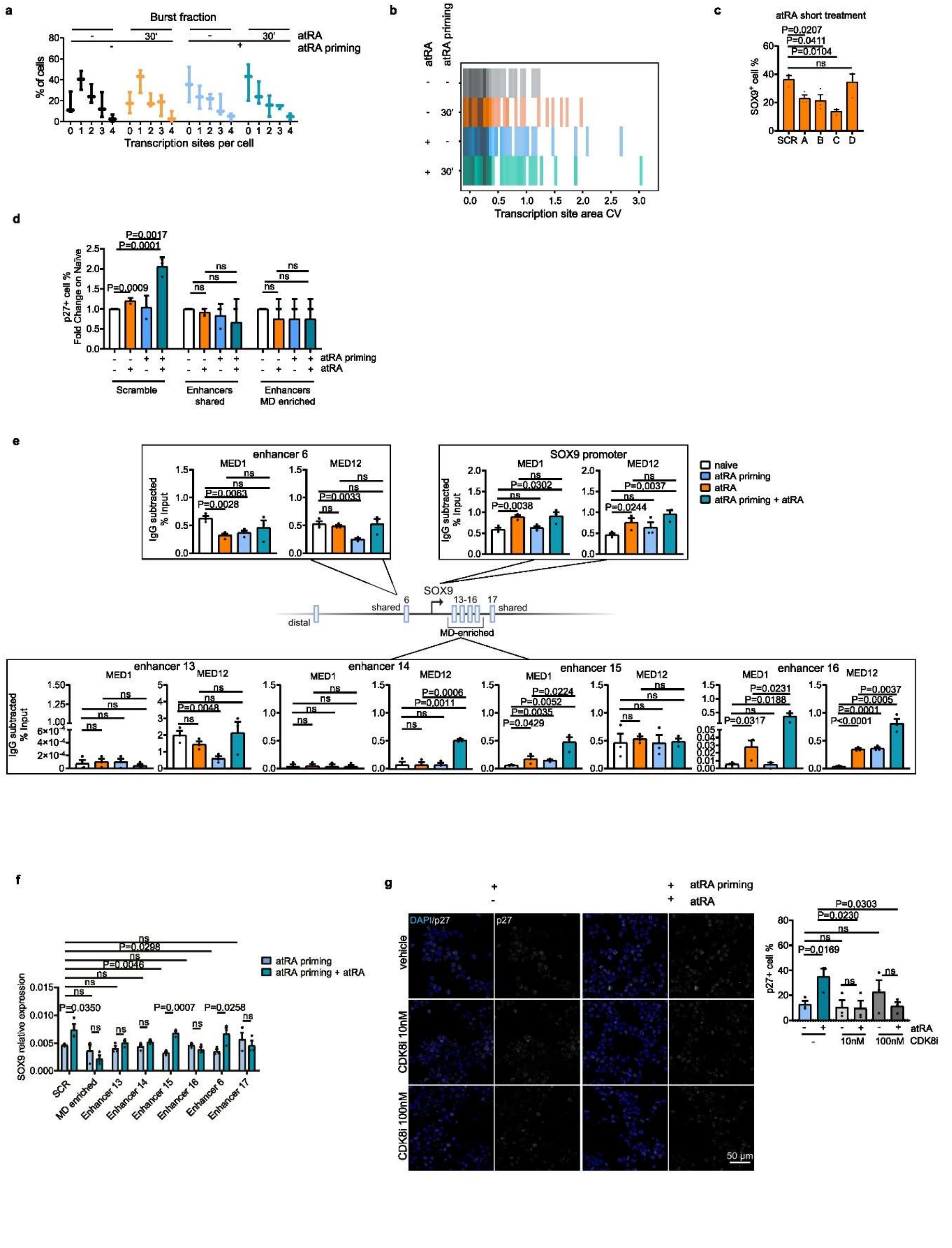

Supplementary Fig. 6: RA-mediated transcriptional memory relies on enhancer activity

(**a**) Boxplot of the percentage of cells showing different numbers of transcription sites for *SOX9* in naïve and primed MD cells, after 30 minutes treatment with vehicle or atRA. (**b**) Heatmap of variation coefficient of SOX9 nascent RNA foci area in naïve and primed MD cells after 30 minute treatment with vehicle or atRA. (**c**) Barplots of the percentage of SOX9 expressing cells determined by RNA PrimeFlow Assay in naïve MD cells treated with atRA and carrying sgRNAs targeting a scramble sequence (SCR), the SOX9 promoter (A), the MD-enriched enhancers (B), the shared enhancers (C), and a distal, unrelated region (D). (**d**) Representative images of RNA-smFISH for SOX9 nascent transcript in naïve and primed MD cells, after short treatment with vehicle or atRA and expressing sgRNAs targeting a scramble sequence (SCR), the MD-enriched enhancers (B) and the hared enhancers (C). H2B_mCherry, yellow; KRAB, green; gRNA_BFP: gray; SOX9 nascent transcript, red; scale bar = 5 µm. (e) Barplots showing relative enrichment of MED1 and MED12 binding in SOX9 promoter or indicated enhancer regions in the SOX9 locus, comparing naive and primed cells after short atRA or vehicle treatment. (f) Barplot of SOX9 relative expression in primed MD cells carring sgRNAs targeting a scramble region (SCR), the cluster of MD-enriched enhancers (MD enriched), single MD-enriched enhancers (Enhancers #13, #14, #15 and #16) or single shared enhancers (Enhancers #6 and #17) after vehicle or atRA treatment. (g) Representative images and quantification of p27 immunostaining in primed MD cells, after treatment with vehicle, CDK8i 10 or 100nM and subsequent treatment with atRA or vehicle. Barplot of p27 positive cell percentage. DAPI, blue; p27, white. scale bar = 50 µm. The barplots in (c)-(g) are means of three independent biological replicates +/- S.E.M. The box plots in (a) indicate median

values (middle lines), first and third quartiles (box edges) and 10th and 90th percentiles (error bars) retrieved from three independent biological replicates. The heatmap in (b) indicates data retrieved from three independent biological replicates. Statistical significance was determined by one-tailed unpaired student's t-test.

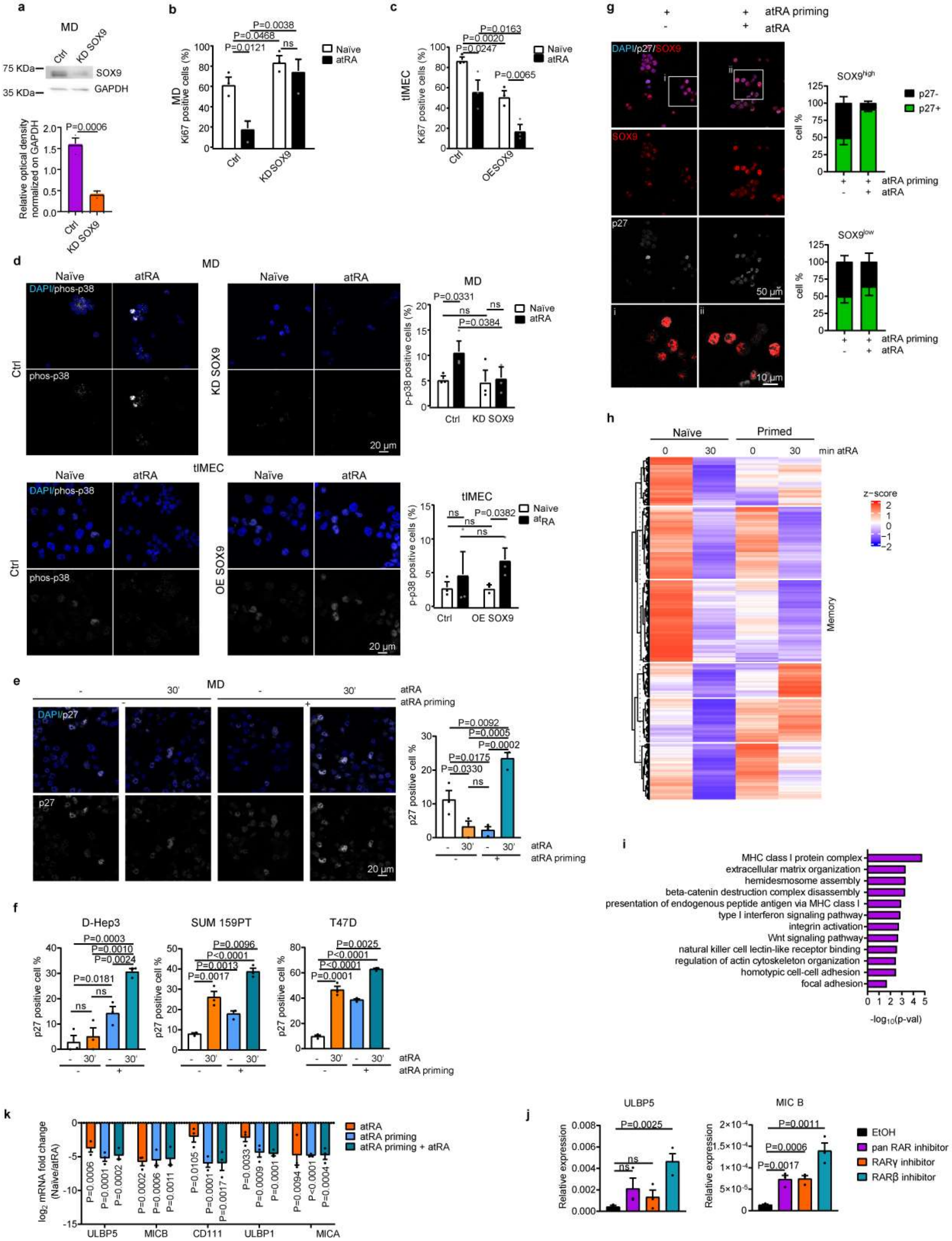

a
MD
Ctrl
KD SOX9
75 KDa
35 KDa
SOX9
GAPDH
Relative optical density normalized on GAPDH
P=0.0006
b
P=0.0038
P=0.0468
P=0.0121
ns
Naïve
atRA
MD Ki67 positive cells (%)
KD SOX9
c
P=0.0163
P=0.0020
P=0.0247
P=0.0065
tIMEC Ki67 positive cells (%)
OE SOX9
g
atRA priming
atRA
DAPI/p27/SOX9
SOX9
p27
50 µm
10 µm
SOX9high
SOX9low
p27-
p27+
cell %
d
MD
Naïve
atRA
DAPI/phos-p38
phos-p38
20 µm
P=0.0331
P=0.0384
p-p38 positive cells (%)
tIMEC
P=0.0382
e
MD
DAPI/p27
p27
P=0.0092
P=0.0005
P=0.0002
P=0.0175
P=0.0330
p27 positive cell %
h
Naïve
Primed
min atRA
z-score
Memory
i
MHC class I protein complex
extracellular matrix organization
hemidesmosome assembly
beta-catenin destruction complex disassembly
presentation of endogenous peptide antigen via MHC class I
type I interferon signaling pathway
integrin activation
Wnt signaling pathway
natural killer cell lectin-like receptor binding
regulation of actin cytoskeleton organization
homotypic cell-cell adhesion
focal adhesion
-log10(p-val)
f
D-Hep3
SUM 159PT
T47D
P=0.0003
P=0.0010
P=0.0024
P=0.0181
P=0.0096
P<0.0001
P=0.0013
P=0.0017
P=0.0025
P<0.0001
P<0.0001
P=0.0001
k
log2 mRNA fold change (Naïve/atRA)
atRA
atRA priming
atRA priming + atRA
ULBP5
MICB
CD111
ULBP1
MICA
j
ULBP5
MIC B
P=0.0025
P=0.0011
P=0.0006
P=0.0017
Relative expression
EtOH
pan RAR inhibitor
RARγ inhibitor
RARβ inhibitor

Supplementary Fig. 7: SOX9 drives metastatic-specific cell dormancy through RA response

(**a**) Western blot of SOX9 in MD shGFP (Ctrl) and MD shSOX9 (KD SOX9); GAPDH was used as loading control. Barplot of signal quantification obtained from three biological replicates is reported. (**b**) Barplot of the percentage of Ki67 positive cells in naïve Ctrl or KD SOX9 MD samples, after treatment with vehicle or atRA for 72h. (**c**) Barplot of the percentage of Ki67 positive cells in naïve tIMEC with or without overexpression of SOX9 (OE SOX9) after treatment with vehicle or atRA for 72h. (**d**) Representative images and quantification of phospho-p38 immunostaining positive cells in naïve Ctrl or KD SOX9 MD cells (upper panels) and tIMEC with or without overexpression of SOX9 (lower panels), after treatment with vehicle or atRA for 72h. phospho-p38, white; DAPI, blue; scale bar = 20 µm. (**e**) Representative images and quantification of p27 positive cells in naïve and primed MD samples, after 30 minutes treatment with vehicle or atRA. p27, white; DAPI, blue; scale bar = 20 µm. (**f**) Barplots of p27 positive cells in naïve or primed D-Hep3, SUM159PT or T47D cells after 30 minutes treatment with vehicle or atRA. (**g**) Representative images and quantification of SOX9 immunostaining in MD p27-Venus primed cells, after treatment with atRA or vehicle. Barplot of p27 positive and negative cells in SOX9 high/low cell populations. SOX9, red; DAPI, blue; p27, white. scale bar = 50 µm (upper panels); scale bar = 10 µm (zoom). (h) Heatmap showing the changes in expression level (z-score of spike-in normalized FPKM values) of genes filtered for a log2fold change > 0.5 between the naïve and the short-treated samples identified by EU RNA-seq in three biological replicates. Cluster analyses highlighted repressed memory genes. (i) Barplot showing GO terms associated with repressed memory genes and corresponding -log10(p-value). (k) MIC-A, CD111, ULBP1, MIC-B and ULBP5 relative log2

fold change expression levels in primed MD cells after treatment with vehicle or atRA, with respect to the naïve condition. (j) Relative expression levels of ULBP5 (left) and MIC-B (right) in MD cells after 72h treatment with etOH or 1µM RARγ, RARβ or pan-RAR. The barplots in (a-g) and (k-j) are means of three independent biological replicates +/- S.E.M. Statistical significance was determined by one-tailed unpaired student's t-test.

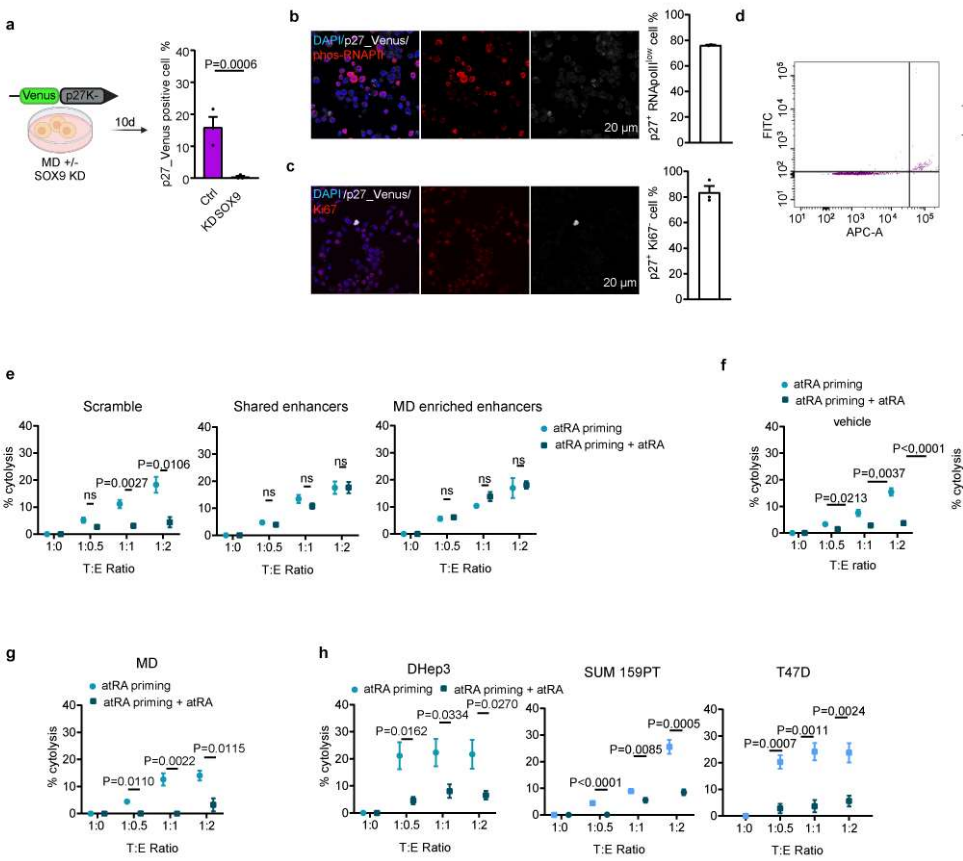
a
Venus
p27K-
10d
MD +/-
SOX9 KD
p27_Venus positive cell %
P=0.0006
Ctrl
KDSOX9
b
DAPI/p27_Venus/
phos-RNAPII
20 µm
p27+ RNApolII low cell %
c
DAPI/p27_Venus/
Ki67
20 µm
p27+ Ki67- cell %
d
FITC
APC-A
CVM+/p27+
e
Scramble
Shared enhancers
MD enriched enhancers
atRA priming
atRA priming + atRA
% cytolysis
T:E Ratio
P=0.0027
P=0.0106
ns
f
atRA priming
atRA priming + atRA
vehicle
CDK8i
P=0.0213
P=0.0037
P<0.0001
T:E ratio
g
MD
atRA priming
atRA priming + atRA
P=0.0110
P=0.0022
P=0.0115
T:E Ratio
h
DHep3
SUM 159PT
T47D
atRA priming
atRA priming + atRA
P=0.0162
P=0.0334
P=0.0270
P<0.0001
P=0.0085
P=0.0005
P=0.0007
P=0.0011
P=0.0024
T:E Ratio

Supplementary Fig. 8: RA-mediated quiescence induction allows metastatic cells to escape NK-mediated immuno-surveillance

(**a**) Schematic representation of the establishment of mVenus-p27K⁻ reporter systems (left) (created with BioRender.com) to detect quiescent cells and barplot showing FACS analysis quantification of the percentages of p27 positive MD cells expressing shGFP (Ctrl) or shSOX9 (KD SOX9). (**b**) Representative images of Ser2ph-RNAP immunostaining in mVenus-p27K⁻ MD cells. mVenus-p27K⁻, white; RNA POL II, red; DAPI, blue; scale bar = 20 μm. Barplots of p27positive/Ser2ph-RNAP^{low} cells. (**c**) Representative images of Ki67 immunostaining in mVenus-p27K MD cells. p27-Venus, white; Ki67, red; DAPI, blue; scale bar = 20 μm. Barplots of p27+/Ki67- cell percentages. (**d**) Representative distribution of dye retaining cells and quantification of dye retaining cells (CMV) and of p27ositive cell obtained from FACS analysis of CellVue Maroon (CVM) staining for dye retention assay (APC-A), combined with p27 evaluation (FITC) in mVenus-p27K MD cells. (e) Average percentage of NK-mediated cytotoxicity of primed MD cells carrying sgRNAs targeting a scramble sequence (Scramble), the MD-enriched enhancers and the shared enhancers, for different effector:target (E:T) ratios and treated with vehicle or atRA. (f) Average percentage of NK-mediated cytotoxicity of primed MD cells after vehicle or 10nM CDK8i treatment and subsequent vehicle or atRA treatment for different effector:target (E:T). (g-**h**) Barplots of average percentage of NK-mediated cytotoxicity of primed MD (g) or D-Hep3, SUM159PT and T47D (h) cells for different effector:target (E:T) ratios and treated with vehicle or atRA. The barplots in (a-d) are means of three independent biological replicates +/- S.E.M. Mean values shown in (e and g-h) are retrieved from four independent experimental groups. Mean values shown in (f) are retrieved from five

independent experimental groups. Statistical significance was determined by one-tailed unpaired student's t-test.

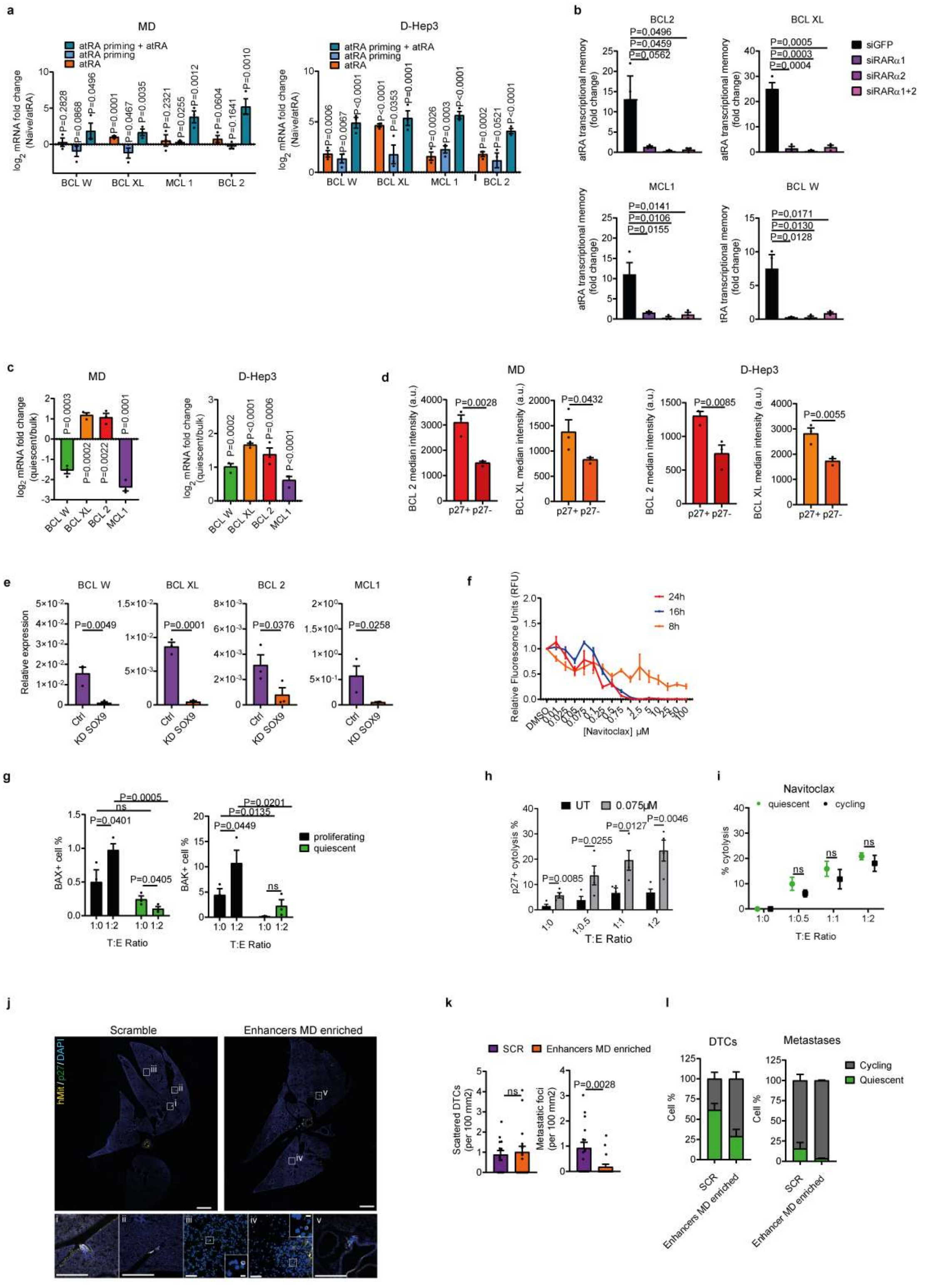

Supplementary Fig. 9: Quiescent metastatic cells are characterized by a higher mitochondrial apoptotic threshold

(**a**) BCL-W, BCL-XL, MCL1 and BCL-2 relative log2 fold change expression levels in primed MD cells (left) and D-Hep3 cells (right) after treatment with vehicle or atRA, with respect to the naïve condition.(**b**) Barplots of BCL2, BCL XL, MCL1 and BCL W relative expression levels in primed MD cells transiently transfected with RARα-targeting siRNAs or with control siRNA and treated with vehicle or atRA for 30 minuted. Effect of RARα knock-down is represented as fold change of expression of the target amplicon in primed cells after 30 minutes atRA treatment with respect to vehicle-treated MD cells. (**c**) Relative expression levels of BCL-W, BCL-XI, BCL 2 and MCL1 in quiescent MD cells (left) and D-Hep3 cells (right) with respect to the bulk population, and represented as log2 fold change. (**d**) BCL 2 and BCL-XL protein median intensity in quiescent (p27+) or proliferative (p27-) MD (left) and D-Hep3 (right) as detected by FACS analysis. (**e**) Relative expression levels of BCL-W, BCL-XI, BCL 2 and MCL1 in MD cells with or without SOX9 knock-down. (**f**) Diagram showing MD cell viability after treatment with vehicle or [0.01-100uM] Navitoclax for 8, 16 or 24h. (**g**) Barplots of quiescent and proliferating BAX and BAK positive cell percentages after FACS analysis of MD p27_Venus cells (target) following 4 hours co-culture with NK92 cells (effector) in two different target:effector ratios. (**h**) Barplots of average percentage of NK-mediated cytotoxicity of p27 positive MD cells for different effector:target (E:T) ratios after 24h treatment with vehicle or 0.075uM Navitoclax. (**i**) Average percentage of NK-mediated cytotoxicity of proliferating and quiescent MD cells for different effector:target (E:T) ratios, upon treatment with Navitoclax. Merge of three independent biological replicates are shown. (**j**)DTCs and metastatic foci distribution within the lungs of nude mice injected with MD cells carrying

sgRNAs targeting a scramble sequence (SCR) or the MD-enriched enhancers. Top, tile scans of entire lung sections; scale bar: 1mm. Bottom, representative images of metastatic foci (corresponding to i, ii and v from top image; scale bar = 1mm) and quiescent DTCs (corresponding to iii and iv from top image; scale bar = 50μm; further zoom scale bar = 10μm). Human mitochondria: yellow; p27: green; DAPI: blue. (**k**) Barplots of scattered DTCs and metastatic foci in lungs of mice injected with MD cells carrying sgRNAs targeting a scramble sequence (Scramble) or the MD-enriched enhancers, normalized to the lung area analyzed (n = 24; data combined from three experimental groups). (**l**) Barplots of the percentages of cycling or quiescent cells retrieved as scattered DTCs or lung metastases in nude mice injected with MD cells carrying sgRNAs targeting a scramble sequence (Scramble) or the MD-enriched enhancers (n = 24; data combined from three experimental groups). The barplots in (a-e) and (g) are means of three independent biological replicates +/- S.E.M. Mean values shown in (h) are retrieved from four independent experimental groups. Mean values shown in (ii) are retrieved from three independent experimental groups. Statistical significance was determined by one-tailed unpaired student's t-test.

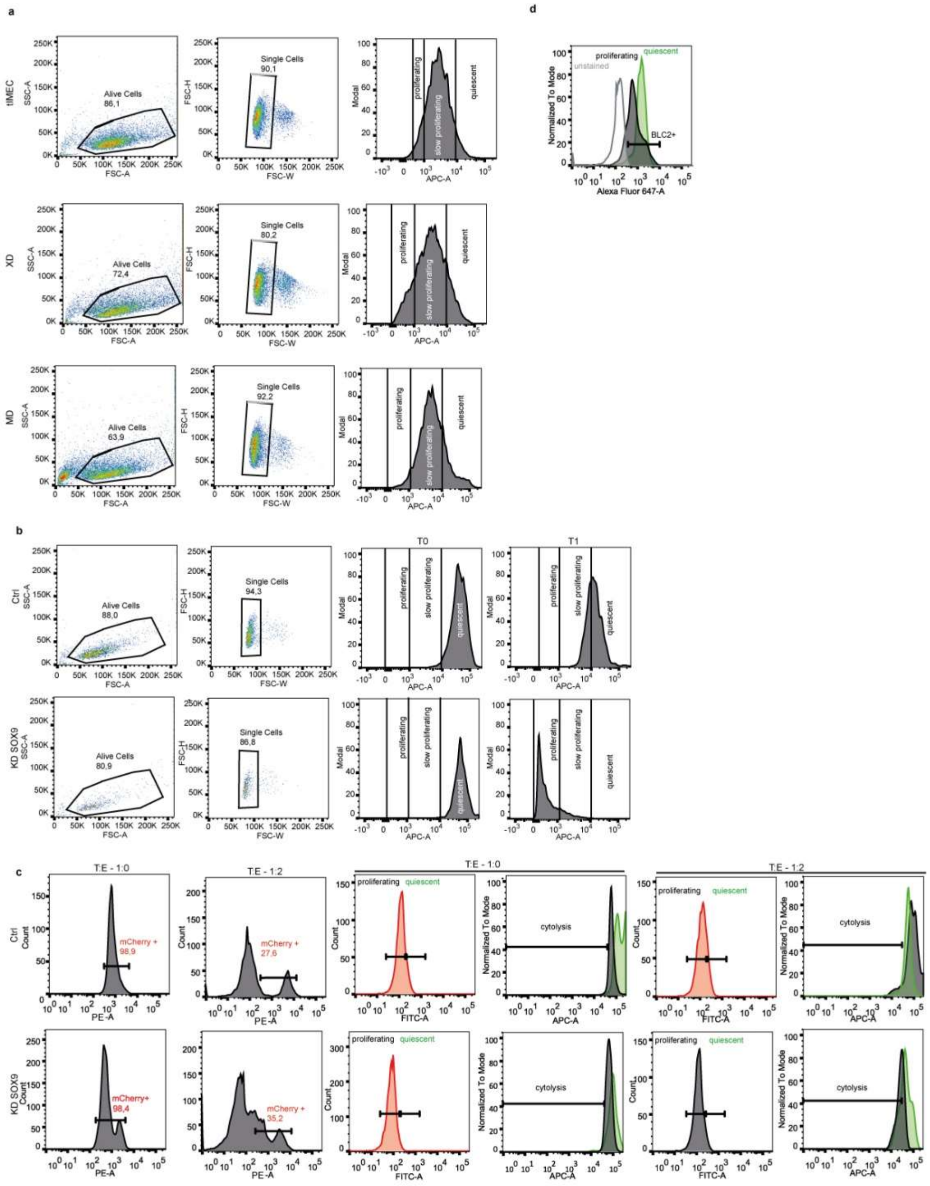
a
tMEC
XD
MD
SSC-A
FSC-A
FSC-H
FSC-W
Alive Cells
86,1
72,4
63,9
Single Cells
90,1
80,2
92,2
Modal
APC-A
proliferating
slow proliferating
quiescent
b
Ctrl
KD SOX9
Alive Cells
88,0
80,9
Single Cells
94,3
86,8
T0
T1
c
T:E - 1:0
T:E - 1:2
Count
PE-A
mCherry +
98,9
27,6
mCherry+
98,4
mCherry +
35,2
FITC-A
Normalized To Mode
cytolysis
d
unstained
BLC2+
Alexa Fluor 647-A

Supplementary Figure 10: Gating strategies for FACS analysis

(**a**) Gating strategy to analyze (left to right): alive cells (FSC-A vs SSC-A), singlets (FSC-W vs FSC-H) and APC-A intensity in tIMEC, XD and MD cells. This strategy was used for the analysis shown in Fig. 1 a. (**b**) Gating strategy to analyze (left to right): alive cells (FSC-A vs SSC-A), singlets (FSC-W vs FSC-H) and APC-A intensity in naïve Ctrl MD cells or MD cells after SOX9 knock-down, at the time points reported in Fig. 7b. The same gating strategy was applied in all other conditions shown in Fig. 7b, and the same gating strategy for morphological features (viability and singlets) was applied in Fig. 7d, f. (**c**) Gating strategy applied on morphologically selected cells (shown in (b)) to analyze H2B_mCherry positive cells (PE-A), which distinguish MD cells from NK92 cells, quiescent and proliferating cells on the basis of the p27_Venus reporter (FITC-A) and cells subjected to cytolysis, based on the CellMask DeepRed staining signal (APC-A); representative gating strategies are shown for T:E ratios 1:0 and 1:2. The same gating strategy was applied for all other T:E ratios shown in Fig. 7d, as well as for analysis in Fig. 7f. Morphologically selected cells (as shown in (a)) were analyzed with the same gating strategy for FITC-A positivity as in (c) in Supplementary Fig. 1d and Supplementary Fig. 8 a, for FITC-A and APC-A positivity in Supplementary Fig. 8d, for PE-A positivity, FITC-A positivity and APC-A positivity in NK-induced cytotoxicity experiments shown in Supplementary Fig. 8e, f, g, h and Supplementary Fig. 9 g, h and i. (**d**) Gating strategy applied in Supplementary Fig. 9 d to evaluate BCL2 and BCL-XL positivity on morphologically selected cells, further gated based on FITC-A intensity as shown in (c). Representative gates are shown for MD cells stained for BCL-2.

Source Data for Supplementary Figure 7

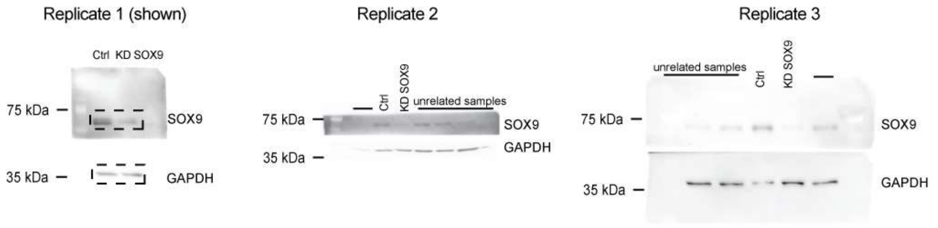

Supplement: Supplementary file 1 — Supplementary Information [file 41467_2024_46524_MOESM1_ESM.pdf]
