# Supplementary material for: Oncogenic enhancers prime quiescent metastatic cells to escape NK immune surveillance by eliciting transcriptional memory
Source: Nat Commun. 2024 Mar 19;15:2198. doi: 10.1038/s41467-024-46524-0 (PMC10951355; doi:10.1038/s41467-024-46524-0)
Supplement: Supplementary file 3 — Description of Additional Supplementary Files [file 41467_2024_46524_MOESM3_ESM.pdf]

Supplementary Data Legends

Supplementary Data_1:

List of differentially accessible distal peaks with coordinates and cluster information

Supplementary Data_2:

Results of integration of ATAC-seq and RNA-seq for all differential distal peaks.

Supplementary Table_3:

Results of integration of ATAC-seq and RNA-seq for all differential distal peaks. P-value was calculated through one-sided Student's t-test within the IMAGE algorithm and to define the false discovery rate (FDR) multiple test corrections via the Benjamini-Hochberg procedure was applied.

Supplementary Data_4:

Results of integration of ATAC-seq and RNA-seq for all distal peaks. P-value was calculated through one-sided Student's t-test within the IMAGE algorithm and to define the false discovery rate (FDR) multiple test corrections via the Benjamini-Hochberg procedure was applied.

Supplementary Data_5:

Results of integration of ATAC-seq and RNA-seq for all peaks linked to cliques identified by H3K27ac HiChIP. P-value was calculated through one-sided Student's t-test within the IMAGE algorithm and to define the false discovery rate (FDR) multiple test corrections via the Benjamini-Hochberg procedure was applied.

Supplementary Data_6:

Genes associated with an increase in expression in pulse vs. naïve cells and corresponding clusters of expression trajectories

Supplementary Data_7:

GO terms associated with responsive genes (cluster 4). P-value was calculated through one-sided Student's t-test version of Fisher's exact test within EnrichR.

Supplementary Data_8:

Genes associated with a decrease in expression in pulse vs. naïve cells and corresponding clusters of expression trajectories

Supplementary Data_9:

GO terms associated with memory genes showing a lower expression in pulse vs. naïve (cluster 3). P-value was calculated through one-sided Student's t-test version of Fisher's exact test within EnrichR.

Supplementary Data_10:

List of oligonucleotides used in this work for epigenome editing, gene expression analysis and smFISH.

Supplementary Data_11:

List of antibodies used in this work.
