# Supplementary material for: Oncogenic enhancers prime quiescent metastatic cells to escape NK immune surveillance by eliciting transcriptional memory
Source: Nat Commun. 2024 Mar 19;15:2198. doi: 10.1038/s41467-024-46524-0 (PMC10951355; doi:10.1038/s41467-024-46524-0)
Supplement: Supplementary file 15 — Reporting Summary [file 41467_2024_46524_MOESM15_ESM.pdf]

nature portfolio

Corresponding author(s): Alessio Zippo

Last updated by author(s): 31/01/2024

Reporting Summary

Nature Portfolio wishes to improve the reproducibility of the work that we publish. This form provides structure for consistency and transparency in reporting. For further information on Nature Portfolio policies, see our Editorial Policies and the Editorial Policy Checklist.

Statistics

For all statistical analyses, confirm that the following items are present in the figure legend, table legend, main text, or Methods section.

n/a	Confirmed	
☐	☒	The exact sample size (*n*) for each experimental group/condition, given as a discrete number and unit of measurement
☐	☒	A statement on whether measurements were taken from distinct samples or whether the same sample was measured repeatedly
☐	☒	The statistical test(s) used AND whether they are one- or two-sided<br>*Only common tests should be described solely by name; describe more complex techniques in the Methods section.*
☒	☐	A description of all covariates tested
☒	☐	A description of any assumptions or corrections, such as tests of normality and adjustment for multiple comparisons
☐	☒	A full description of the statistical parameters including central tendency (e.g. means) or other basic estimates (e.g. regression coefficient) AND variation (e.g. standard deviation) or associated estimates of uncertainty (e.g. confidence intervals)
☐	☒	For null hypothesis testing, the test statistic (e.g. *F*, *t*, *r*) with confidence intervals, effect sizes, degrees of freedom and *P* value noted<br>*Give P values as exact values whenever suitable.*
☒	☐	For Bayesian analysis, information on the choice of priors and Markov chain Monte Carlo settings
☐	☒	For hierarchical and complex designs, identification of the appropriate level for tests and full reporting of outcomes
☐	☒	Estimates of effect sizes (e.g. Cohen's *d*, Pearson's *r*), indicating how they were calculated

Our web collection on statistics for biologists contains articles on many of the points above.

Software and code

Policy information about availability of computer code

Data collection	LAS AF Software 2.6.0 was used for confocal imaging; BD FACSDivaTM Software was used for acquisition of flow cytometry experiments; Image lab 2.0.1 software was used for acquisition of western blot images.<br>NIS elements (AR 5.11) was used for RNA FISH combined with immunofluorescence acquisition and SIM microscopy.
Data analysis	Fiji (1.52) was used for immunofluorescence data analysis. FlowJo X 10.0.7r2 was used for FACS analysis.For Genome-wide analysis (ATAC-seq, RNA-seq, HiChIP, EU-RNA-seq and Cut&Run), the following tools were used:<br><br>ATAC-seq:Trimmomatic v0.39, BowTie2, samtools v1.10, HOMER v4.11, edgeR v3.20.9 and limma v3.34.9.RNA-seq: FastQC, Tophat2, EnrichR HiChIP: HiC-Pro, FitHiChIP, pgltools, graphtool (v.2.45) EU-RNA-seq: Deeptools<br><br>v3.1.2 Cut&Run: MACS2 Visualization: ggplot2 (v 3.3.3), ComplexHeatmap (v 2.6.2) VennDiagram (v 1.7.1), UpSetR (v 1.4.0), Sushi (v 1.28.0), seaborn (v.0.11.2), matplotlib (v.3.5.2).<br>Custom-made code for the analysis and integration of HiChIP data is available at the GitHub repository (https://github.com/zippolab/Michelatti_et_al)

For manuscripts utilizing custom algorithms or software that are central to the research but not yet described in published literature, software must be made available to editors and reviewers. We strongly encourage code deposition in a community repository (e.g. GitHub). See the Nature Portfolio guidelines for submitting code & software for further information.

Data

Policy information about availability of data

All manuscripts must include a data availability statement. This statement should provide the following information, where applicable:

- Accession codes, unique identifiers, or web links for publicly available datasets
- A description of any restrictions on data availability
- For clinical datasets or third party data, please ensure that the statement adheres to our policy

The RNA sequencing, ATAC-seq, CUT&RUN and HiChIP raw data have been deposited in the Gene Expression Omnibus database under the accession GSE211610 (Token: ozwfemqmjfsddex). The scRNA-seq datasets were retrieved from the Gene Expression Omnibus database under the accession GSE158399. HiChIP datasets from HCC1599 and MB157 cell lines were retrieved from GSE116876. Accessibility datasets for the different tumour and healthy tissues were retrieved from GSE165659.

Research involving human participants, their data, or biological material

Policy information about studies with human participants or human data. See also policy information about sex, gender (identity/presentation), and sexual orientation and race, ethnicity and racism.

Reporting on sex and gender	N/A
Reporting on race, ethnicity, or other socially relevant groupings	N/A
Population characteristics	N/A
Recruitment	N/A
Ethics oversight	N/A

Note that full information on the approval of the study protocol must also be provided in the manuscript.

Field-specific reporting

Please select the one below that is the best fit for your research. If you are not sure, read the appropriate sections before making your selection.

[x] Life sciences [ ] Behavioural & social sciences [ ] Ecological, evolutionary & environmental sciences

For a reference copy of the document with all sections, see nature.com/documents/nr-reporting-summary-flat.pdf

Life sciences study design

All studies must disclose on these points even when the disclosure is negative.

Sample size	No sample size calculations were performed. The sample size was determined by the number of biological replicates required for ensuring statistical significance, and the sample sizes were chosen to support meaningful conclusions. The number of biological replicates is reported in the relevant figure legends in the manuscript.
Data exclusions	No data were excluded.
Replication	For each experiment, the number of biological independent sample is reported in the figure legend.
Randomization	Randomization is not relevant because we did not use different experimental groups.
Blinding	All the experiments were conducted using the same experimental conditions, both in vitro (cell culture) and in vivo (mice models). For this reason data collection and analyses of all studies were conducted not blinding.

Reporting for specific materials, systems and methods

We require information from authors about some types of materials, experimental systems and methods used in many studies. Here, indicate whether each material, system or method listed is relevant to your study. If you are not sure if a list item applies to your research, read the appropriate section before selecting a response.

Materials & experimental systems

n/a	Involved in the study
	x Antibodies
	x Eukaryotic cell lines
x	Palaeontology and archaeology
	x Animals and other organisms
x	Clinical data
x	Dual use research of concern
x	Plants

Methods

n/a	Involved in the study
	x ChIP-seq
	x Flow cytometry
x	MRI-based neuroimaging

Antibodies

Antibodies used

Target | Company | Catalog number | Application
Ki67 | Leica Microsystems | KI67-MM1-L-CE | IF
phospho-p38 | ThermoFisher | 44-684G | IF
SOX9 | Millipore | AB5535 | IF/WB/Cut&Run
p27-KIP1 | Cell Signalling | 3686S |IF/FACS
BMI-1 | Millipore | 05-637 | IF
BRD4 | Abcam | ab128874 | smFISH+IF
human Mitochondria | Abcam | ab92824 | IF on tissues
GFP | Cell Signalling | 2956 | IF on tissues
H3K27ac | abcam | ab4729 | Cut&Run, HiChIP
H3K4me1 | abcam | ab8895 | Cut&Run
MED1 | abcam | ab64965 | Cut&Run
H3K4me3 | Millipore | 07-473 | Cut&Run
IgG | Millipore | PP64B | Cut&Run
GAPDH (6C5)| Santa Cruz | sc-32233 | WB
BCL-2 | Abcam | ab182858 | FACS
BCL-XL | Cell Signalling 2764 | FACS
BAX | Santa Cruz | sc-23959 | FACS
BAK | GeneTex | GTX10808 | FACS
Med1 | Abcam | ab64965 | ChIP
Med12 | Bethyl | A300-774A | ChIP
IgG |Proteintech | SA00001-2 | ChIP

Validation

Target | Company | Catalog number | Validation
Ki67 | Leica Microsystems | KI67-MM1-L-CE | This antibody has been validated for immunohistochemistry (DOI: 10.1242/dev.133843); reactivity: African green monkey, human, mouse, Rhesus monkey.
phospho-p38 | ThermoFisher | 44-684G | This antibody has been validated for Western Blot, Immunohistochemistry, Immunofluorescence, Flow Cytometry and IP (DOI: 10.1038/s41598-020-69810-5; DOI: 10.1136/gut.2006.104372; DOI: 10.4161/auto.29456; DOI: 10.1073/pnas.1017340108); reactivity: Human, Rat.
SOX9 | Millipore | AB5535 | This antibody has been validated for Immunohistochemistry, Western Blot; ChIP; Immunofluorescence (DOI: 10.1016/j.celrep.2015.06.013; DOI: 10.1371/journal.pone.0022616; DOI: 10.1371/journal.pone.0035136; DOI: 10.1158/0008-5472.CAN-11-3660); reactivity: Human, Mouse, Rabbit, Chicken.
p27-KIP1 | Cell Signalling | 3686S | This antibody has been validated for Western Blot, Immunoprecipitation, Immunofluorescence, Flow Cytometry; reactivity: Human, Rabbit Monkey.
BMI-1 | Millipore | 05-637 | This antibody has been validated for western blotting, immunocitochemistry and immunoprecipitation (doi: 10.1152/ajpgi.00299.2012, doi: 10.1016/j.celrep.2015.05.027); reactivity: Human, Mouse, Rat, Rabbit.
BRD4 | Abcam | ab128874 | This antibody has been validated for western blotting, immunohistochemistry, immunofluorescence and immunoprecipitation (doi: 10.1126/science.aar3958); reactivity: Human, Mouse, Rat.
human Mitochondria | Abcam | ab92824 | This antibody has been validated for Western Blot, Immunohistochemistry, Immunofluorescence, Flow Cytometry (DOI: 10.1111/cas.14890; DOI: 10.1038/s41419-020-2432-1; DOI: 10.1038/s41388-020-1335-z); reactivity: human.
GFP | Cell Signalling | 2956 | This antibody has been validated for Western Blot and Immunohistochemistry (DOI: 10.7554/eLife.79128; DOI: 10.1038/s41389-021-00372-5); reactivity: all species.
H3K27ac | abcam | ab4729 | This antibody has been validated for western blotting, immunoprecipitation and immunocitochemistry; reactivity: Human, Mouse, Rat.
H3K4me1 | abcam | ab8895 | This antibody has been validated for western blotting,

immunoprecipitation and immunocitochemistry (DOI: 10.1371/journal.pone.0225180); reactivity: Human, Mouse.
MED1 | abcam | ab64965 | This antibody has been validated for western blotting and immunohistochemistry (doi: 10.1126/ science.aar3958); reactivity: Human, Mouse.
H3K4me3 | Millipore | 07-473 | This antibody has been validated for Immunocytochemistry; reactivity: Human, Mouse.
IgG | Millipore | PP64B | This antibody has been validated for ELISA.
GAPDH (6C5)| Santa Cruz | sc-32233 | This antibody has been validated for Western Blot, Immunoprecipitation and Immunofluorescence (DOI: 10.1038/s41467-022-29120-y; DOI: 10.1038/
BCL-2 | Abcam | ab182858 | FACS | This antibody was validated for FACS analysis by the manufacturer. Reactivity: Mouse, Human
BCL-XL | Cell Signalling | 2764 | FACS | This antibody was validated for FACS (DOI: 10.1038/ s41418-021-00816-w). Reactivity: Human, Mouse, Rat, Monkey
BAX | Santa Cruz | sc-23959 | FACS | This antibody was validated for FACS (https://doi.org/10.1038/ ncomms7891). Reactivity: human, mouse,and rat
BAK | GeneTex | GTX10808 | FACS| This antibody was validated for FACS (https://doi.org/10.1038/ ncomms7891). Reactivity: Human
Med1 | Abcam | ab64965 | ChIP | This antibody has been validated for ChIP (10.1016/j.molcel. 2019.08.016) Reactivity: Mouse, Human
Med12 | Bethyl | A300-774A | ChIP | This antibody has been validated for IP by the manufacturer. Reactivity: Human, Mouse
IgG | Proteintech | SA00001-2 | ChIP | This antibody has been validated for ChIP (DOI: 10.1158/0008-5472.CAN-17-2356) Reactivity: Rabbit

Eukaryotic cell lines

Policy information about cell lines and Sex and Gender in Research

Cell line source(s)	hTERT-immortalized human mammary epithelial cells (IMEC) were a kind gift from Dr. Michael Cole; NK-92 cell line was a kind gift from Dr. Federica Facciotti. Primary tumour derived cells (XD) and metastasis derived cells (MD) were retrieved from female NOD/SCID mice. T47D and SUM159PT were retrieved from ATCC. D-Hep3 were a kind gift from Dr. Maria Soledad Sosa.
Authentication	None of the cell lines used have been authenticated
Mycoplasma contamination	All cell lines resulted negative for mycoplasma contamination
Commonly misidentified lines (See ICLAC register)	No misidentified cell lines were used

Animals and other research organisms

Policy information about studies involving animals; ARRIVE guidelines recommended for reporting animal research, and Sex and Gender in Research

Laboratory animals	12 weeks female FOXN1nu mice and 4-6 weeks female NOD.Cg-Prkdcscid Il2rgtmWjl/SzJ (NSG) mice from Charles River Laboratories were used in this study.
Wild animals	The study did not involve wild animals.
Reporting on sex	All studies on mice were conducted on female mice to consider the higher incidence of Breast Cancer in females than in males.
Field-collected samples	The study did not involve field-collected samples.
Ethics oversight	All studies on mice were conducted in strict accordance with the institutional guidelines for animal research and approved by the Italian Ministry of Health; Department of Public Health, Animal Health, Nutrition, and Food Safety in accordance to the law on animal experimentation (D.Lgs. 26/2014), Italian Ministry of Health authorization (IACUC 373/2015-PR).

Note that full information on the approval of the study protocol must also be provided in the manuscript.

Plants

Seed stocks	*Report on the source of all seed stocks or other plant material used. If applicable, state the seed stock centre and catalogue number. If plant specimens were collected from the field, describe the collection location, date and sampling procedures.*
Novel plant genotypes	*Describe the methods by which all novel plant genotypes were produced. This includes those generated by transgenic approaches, gene editing, chemical/radiation-based mutagenesis and hybridization. For transgenic lines, describe the transformation method, the number of independent lines analyzed and the generation upon which experiments were performed. For gene-edited lines, describe*

the editor used, the endogenous sequence targeted for editing, the targeting guide RNA sequence (if applicable) and how the editor was applied.

Authentication

Describe any authentication procedures for each seed stock used or novel genotype generated. Describe any experiments used to assess the effect of a mutation and, where applicable, how potential secondary effects (e.g. second site T-DNA insertions, mosiacism, off-target gene editing) were examined.

ChIP-seq

Data deposition

- [x] Confirm that both raw and final processed data have been deposited in a public database such as GEO.
- [ ] Confirm that you have deposited or provided access to graph files (e.g. BED files) for the called peaks.

Data access links
May remain private before publication.

Cut&Run datasets generated in this study have been deposited in the Gene Expression Omnibus under accession number GSE211610

Files in database submission

processed data
tIMEC_H3K4me3.bw
tIMEC_H3K4me1.bw
tIMEC_H3K27ac.bw
tIMEC_MED1.bw
tIMEC_SOX9.bw
tIMEC_IgG.bw
XD5_H3K4me3.bw
XD5_H3K4me1.bw
XD5_H3K27ac.bw
XD5_MED1.bw
XD5_SOX9.bw
XD5_IgG.bw
MD6_H3K4me3.bw
MD6_H3K4me1.bw
MD6_H3K27ac.bw
MD6_MED1.bw
MD6_SOX9.bw
MD6_IgG.bw
raw data
tIMEC_H3K4me3_R1.fastq.gz | tIMEC_H3K4me3_R2.fastq.gz
tIMEC_H3K4me1_R1.fastq.gz | tIMEC_H3K4me1_R2.fastq.gz
tIMEC_H3K27ac_R1.fastq.gz | tIMEC_H3K27ac_R2.fastq.gz
tIMEC_MED1_R1.fastq.gz | tIMEC_MED1_R2.fastq.gz
tIMEC_SOX9_R1.fastq.gz | tIMEC_SOX9_R2.fastq.gz
tIMEC_IgG_R1.fastq.gz | tIMEC_IgG_R2.fastq.gz
XD5_H3K4me3_R1.fastq.gz | XD5_H3K4me3_R2.fastq.gz
XD5_H3K4me1_R1.fastq.gz | XD5_H3K4me1_R2.fastq.gz
XD5_H3K27ac_R1.fastq.gz | XD5_H3K27ac_R2.fastq.gz
XD5_MED1_R1.fastq.gz | XD5_MED1_R2.fastq.gz
XD5_SOX9_R1.fastq.gz | XD5_SOX9_R2.fastq.gz
XD5_IgG_R1.fastq.gz | XD5_IgG_R2.fastq.gz
MD6_H3K4me3_R1.fastq.gz | MD6_H3K4me3_R2.fastq.gz
MD6_H3K4me1_R1.fastq.gz | MD6_H3K4me1_R2.fastq.gz

Genome browser session
(e.g. UCSC)

None

Methodology

Replicates

1

Sequencing depth

8 million reads/sample

Antibodies

H3K27ac|abcam|ab4729|
H3K4me1|abcam|ab8895|
MED1|abcam|ab64965|
H3K4me3|Millipore|07-473|
IgG|Millipore|PP64B|
SOX9|Millipore|AB5535|

Peak calling parameters

Not performed.

Data quality | Quality of raw reads was assessed using FastQC. Reads were trimmed using Trimmomatic to remove low-quality bases and adapter content.
Fraction of reads aligned to human genome (hg19) 75 - 80%, aligned to spike-in mouse (mm10)

Software | DeepTools, HOMER v4.11, limma, edgeR, MACS2

Flow Cytometry

Plots

Confirm that:

- [x] The axis labels state the marker and fluorochrome used (e.g. CD4-FITC).
- [x] The axis scales are clearly visible. Include numbers along axes only for bottom left plot of group (a 'group' is an analysis of identical markers).
- [x] All plots are contour plots with outliers or pseudocolor plots.
- [x] A numerical value for number of cells or percentage (with statistics) is provided.

Methodology

Sample preparation | Tumoroids were retrieved from cell culture; single cell suspensions were obtained through trypsinization, followed by inactivation through FBS. Cell suspensions were then fixed in 1% PFA and immediately anayzed at the FACS instrument.

Instrument | FACS Canto A (BD Bioscience)

Software | FlowJo X 10.0.7r2

Cell population abundance | Cells positive for mVenus-p27K– expression were isolated through cell sorting with FACS Aria IIu (BD Bioscience). The percentage of positive cells prior to sorting was 12.4%. In order to enrich the population for positive cells before sorting, cells were starved with nutrient-depriven medium for 24h prior to sorting.

Gating strategy | Stringent gatings were always used, leaving a significant gap in between negative/positive or low/high populations.

- [x] Tick this box to confirm that a figure exemplifying the gating strategy is provided in the Supplementary Information.
