# Supplementary material for: Oncogenic enhancers prime quiescent metastatic cells to escape NK immune surveillance by eliciting transcriptional memory
Source: Nat Commun. 2024 Mar 19;15:2198. doi: 10.1038/s41467-024-46524-0 (PMC10951355; doi:10.1038/s41467-024-46524-0)
Supplement: Supplementary file 2 — Peer Review File [file 41467_2024_46524_MOESM2_ESM.pdf]

nature portfolio

Peer Review File

Oncogenic enhancers prime quiescent metastatic cells to escape NK immune surveillance by eliciting transcriptional memory

Open Access This file is licensed under a Creative Commons Attribution 4.0 International License, which permits use, sharing, adaptation, distribution and reproduction in any medium or format, as long as you give appropriate credit to the original author(s) and the source, provide a link to the Creative Commons license, and indicate if changes were made. In the cases where the authors are anonymous, such as is the case for the reports of anonymous peer reviewers, author attribution should be to 'Anonymous Referee' followed by a clear attribution to the source work. The images or other third party material in this file are included in the article's Creative Commons license, unless indicated otherwise in a credit line to the material. If material is not included in the article's Creative Commons license and your intended use is not permitted by statutory regulation or exceeds the permitted use, you will need to obtain permission directly from the copyright holder. To view a copy of this license, visit http://creativecommons.org/licenses/by/4.0/.

Editorial Note: This manuscript has been previously reviewed at another journal that is not operating a transparent peer review scheme. This document only contains reviewer comments and rebuttal letters for versions considered at *Nature Communications*.

REVIEWERS' COMMENTS

Reviewer #3 (Remarks to the Author):

The authors have now addressed or provided direct answers to the previously raised questions. The clarifications, data extension to other models, and new analyses are relevant and improved the overall manuscript. The data is interesting and it may benefit the field and broad audience.

Università di Trento,
Dipartimento CIBIO
Via Sommarive 9, POVO

Trento, 2nd of February 2024

Point-by-point rebuttal letter to Reviewers' comments:

Reviewer #3 (Remarks to the Author):
The authors have now addressed or provided direct answers to the previously raised questions. The clarifications, data extension to other models, and new analyses are relevant and improved the overall manuscript. The data is interesting and it may benefit the field and broad audience.

We are pleased that we have correctly addressed all the Reviewer's last comments and that He/She appreciate the relevance and broadness of our findings.

Best regards,

Alessio

Prof. Alessio Zippo
